# First direct evidence of lion hunting and the early use of a lion pelt by Neanderthals

**DOI:** 10.1038/s41598-023-42764-0

**Published:** 2023-10-12

**Authors:** Gabriele Russo, Annemieke Milks, Dirk Leder, Tim Koddenberg, Britt M. Starkovich, M. Duval, J.-X. Zhao, Robert Darga, Wilfried Rosendahl, Thomas Terberger

**Affiliations:** 1 Paleoanthropology, Senckenberg Centre for Human Evolution and Palaeoenvironment, Eberhard Karls University of Tübingen, 72070 Tübingen, Germany; 2 Lower Saxony State Office for Cultural Heritage, Niedersächsisches Landesamt Für Denkmalpflege, 30175 Hanover, Germany; 3 Department of Archaeology, University of Reading, Reading, RG6 6DW UK; 4 Department of Wood Biology and Wood Products, University of Göttingen, 37077 Göttingen, Germany; 5 Senckenberg Centre for Human Evolution and Palaeoenvironment, University of Tübingen, 72070 Tübingen, Germany; 6 Institute for Archaeological Sciences, University of Tübingen, Tübingen, Germany; 7 Centro Nacional de Investigación sobre la Evolución Humana (CENIEH), 09002 Burgos, Spain; 8 Australian Research Centre for Human Evolution (ARCHE), Griffith University, Nathan, QLD 4111 Australia; 9 Palaeoscience Labs, Department Archaeology and History, La Trobe University, Melbourne Campus, Bundoora, VIC 3086 Australia; 10 Radiogenic Isotope Facility, School of Earth and Environmental Sciences, The University of Queensland, Brisbane, QLD 4072 Australia; 11 Südostbayerisches Naturkunde- Und Mammut-Museum, Siegsdorf, Germany; 12 Reiss-Engelhorn-Museen, Zeughaus C5, 68159 Manssnheim, Germany; 13 Curt-Engelhorn-Center of Archaeometrie, C4,8, 68159 Mannheim, Germany; 14 Seminar of Prehistoric Archaeology, University of Göttingen, 37073 Göttingen, Germany

**Keywords:** Archaeology, Cultural evolution

## Abstract

During the Upper Paleolithic, lions become an important theme in Paleolithic art and are more frequent in anthropogenic faunal assemblages. However, the relationship between hominins and lions in earlier periods is poorly known and primarily interpreted as interspecies competition. Here we present new evidence for Neanderthal-cave lion interactions during the Middle Paleolithic. We report new evidence of hunting lesions on the 48,000 years old cave lion skeleton found at Siegsdorf (Germany) that attest to the earliest direct instance of a large predator kill in human history. A comparative analysis of a partial puncture to a rib suggests that the fatal stab was delivered with a wooden thrusting spear. We also present the discovery of distal lion phalanges of at least 190,000 years old from Einhornhöhle (Germany), representing the earliest example of the use of cave lion skin by Neanderthals in Central Europe. Our study provides novel evidence on a new dimension of Neanderthal behavioral complexity.

## Introduction

The relationship between carnivores and hominids has shaped our lineage's evolutionary pathway and behavior since its inception (see^1–5^ among others). Over the millennia, landscape sharing and resource competition have resulted in fatal encounters for hominins^6–10^ but also in increased occasional and systematic large carnivore exploitation^11–18^. Furthermore, large carnivores influenced the cultural behavior of Paleolithic humans, who used their body parts as ornaments, and also depicted them in Paleolithic art (e.g.,^19–21^). Unraveling earlier foundations to these complex relationships is therefore fundamental to the study of human past. Here we report new evidence of interactions between Neanderthals and cave lions from the site of Siegsdorf and Einhornhöhle, both in Germany, and contextualize the new findings with previous archeological and ethnographic studies on human-lion interaction to make inferences about the role of this large predator on human behavior and culture during the Middle Paleolithic.

### Pleistocene lions and humans

Among all the large predators we have encountered during our evolutionary journey, the lion is arguably one of the most charismatic. To this day, it continues to be an icon of popular culture in many traditions worldwide. The story of the lion's dispersal shares some parallels with that of our own. The lion lineage originated in eastern Africa, with the earliest fossils of lion-like *Panthera* dated between 3.8 and 3.6 Ma at Laeotoli, Tanzania^22^. A remarkably rapid dispersal occurred during the Middle Pleistocene, as evidenced by the presence of lion (*P. fossilis*) remains in Western Europe (e.g.,^13,23^). By the Late Pleistocene, the Eurasian cave lion (*Panthera spelaea* Goldfuss, 1810) occupied the key ecological role of apex predator in the Mammoth Steppe trophic chain^24,25^ until its extinction by the end of the Pleistocene^26^, with the youngest fossils dated to ca. 12.5 ka in Central Europe^27^.

Hominins have been interacting with lions since their arrival in Europe, and possibly even earlier^13^. The big cat held perceptible significance for Upper Paleolithic *Homo sapiens* groups in Europe (cf.^28^). This is well illustrated in the Aurignacian by the cave lion depictions in caves of south-eastern France^20,29^, ivory sculptures including the famous *Löwenmensch* (Lion man) and figurines from the Swabian Jura's deposits^30–32^, and perforated cave lion canines worn as personal ornaments^19,33,34^. During the subsequent Gravettian period, cut marked bones prove the exploitation of lions at sites in the Swabian Jura and Moravia^8,17,35^. The discovery of nine distal phalanges with cut marks in later Magdalenian levels from La Garma, in northern Spain, documents the exploitation of a lion pelt by *H. sapiens*^21^. Finally, by the end of the last glaciation, three cave lion silhouettes were engraved on a bone found at La Vache Cave in southwestern France^36^.

Despite the archaeological record demonstrating the importance the cave lion to our species, it remains unclear how other human species interacted with this apex predator, beyond interspecies competition. The sole evidence for hominin-lion interaction during the Lower Paleolithic consists of the butchered remains of a *P. fossilis* at Gran Dolina (level TD10-1), Sierra de Atapuerca, in northern Spain, dated to Marine Isotope Stage (MIS) 9. Although chronologically isolated, this unique find represents the earliest example of exploitation and possible consumption of a large predator by early hominins, including evidence for skinning^13^.

Neanderthals were effective hunters at the top of the food chain and competed with cave lions for prey taxa^37^. They hunted ursids and other carnivores^12,14,15,38,39^, and exploited animal resources not only for subsistence but also for non-utilitarian purposes^40–45^. Nevertheless, during the Middle Paleolithic, evidence for Neanderthal-lion interaction is scarce. Two cut marked lion fibula from level IV of Bolomor Cave (MIS 5), eastern Iberia, attest to the butchery of lion^46^. Cut marked lion bones come also from the Quina Mousterian deposits of Chez Pinaud, Jonzac (MIS 4), in south-western France, that are interpreted as evidence for skinning^47,48^. A cave lion radius with potential cut marks is reported from the Mousterian level F2 of West Cave at Le Portel (MIS 3), in southern France^49^. Late Pleistocene Neanderthals possibly skinned a cave lion (*P. spelaea*) at Caverna delle Fate in Finale Ligure (end of MIS 5 to MIS 3), northern Italy^50^. A partial cave lion skeleton with cutmarks unearthed at Siegsdorf (MIS 3), in south-eastern Germany dates to the end of the Middle Paleolithic, and was originally interpreted as a carcass butchery event (see^51^ and SI 3.1) due to the presence of cutmarks on several skeletal elements.

**Figure 1:**
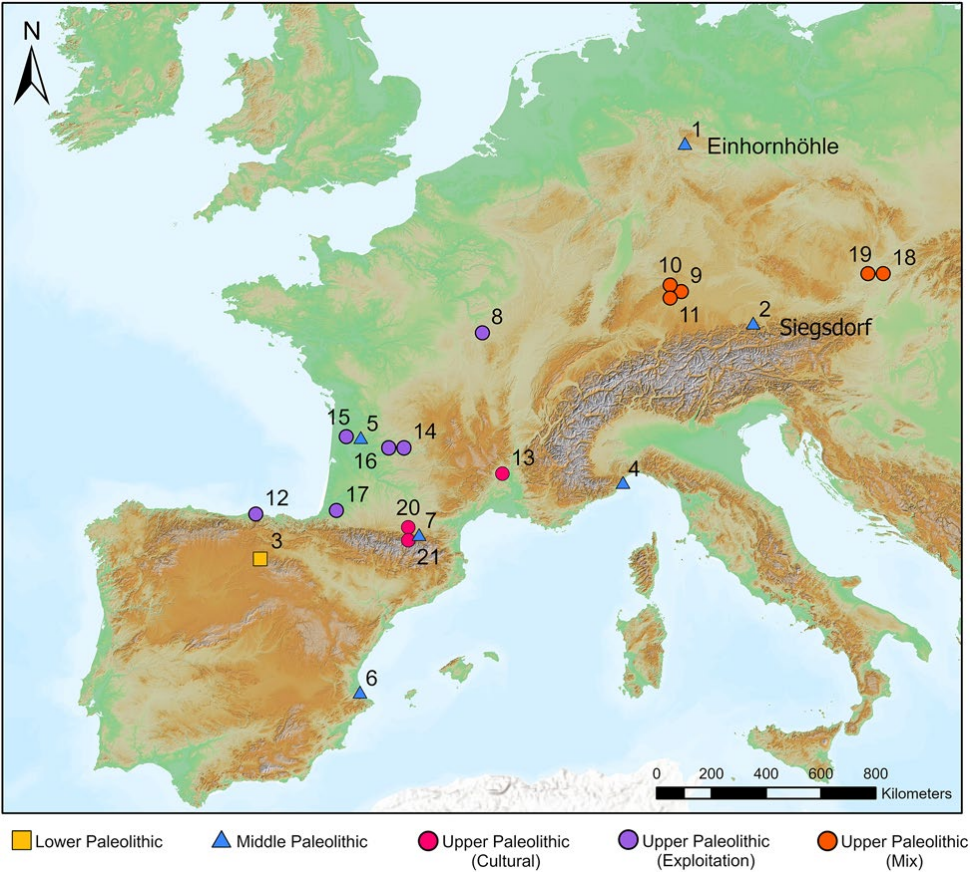
Paleolithic sites with direct evidence of human-cave lion interaction in form of subsistence activity, culture (i.e., rock or mobile art), or both. 1 Einhornhöhle, 2 Siegsdorf, 3 Gran Dolina, 4 Caverna delle Fate, 5 Chez-Pinaud (Jonzac), 6 Cueva de Bolomir, 7 Le Portel, 8 Grotte du Renne, 9 Hohlenstein-Stadel, 10 Vogelherd, 11 Hohle Fels, 12 La Garma, 13 Chauvet, 14 Peyrat (Saint-Rabier), 15 Pair-non-Pair, 16 La Gravette, 17 Grotte Duruthy, 18 Pavlov I, 19 Dolní Věstonice I, 20 Trois-Frères, 21 Grotte de la Vache (Ariège). Map was created with ArchGIS Pro version 3.0.5 and raster files were obtained from The Digital Elevation Model over Europe (EU-DEM). Available online: https://www.eea.europa.eu/data-and-maps/data/copernicus-land-monitoring-service-eu-dem (accessed on 20 February 2023).

This paper presents the taphonomic re-evaluation of the lion skeleton from Siegsdorf and the new evidence from Einhornhöhle (EHH) (MIS 6 to 7) in central Germany. The taphonomic and forensic analysis indicates that the Siegsdorf lion was killed and then butchered by Neanderthals. The cave lion phalanges from Einhornhöhle provide earliest insights into the potential use of lion pelt by Middle Paleolithic hominins, adding to the existing body of evidence that suggests the exploitation of large predators, including previous observations of skinning cut marks on lion phalanges, during the Middle Paleolithic. These new finds represent important additions to the limited Middle Paleolithic record of human-lion interaction and provide new information regarding the behavioral complexity of Neanderthals (Fig. 1).

## Results

### Cave lion from Siegsdorf: killing and carcass butchery

**Figure 2:**
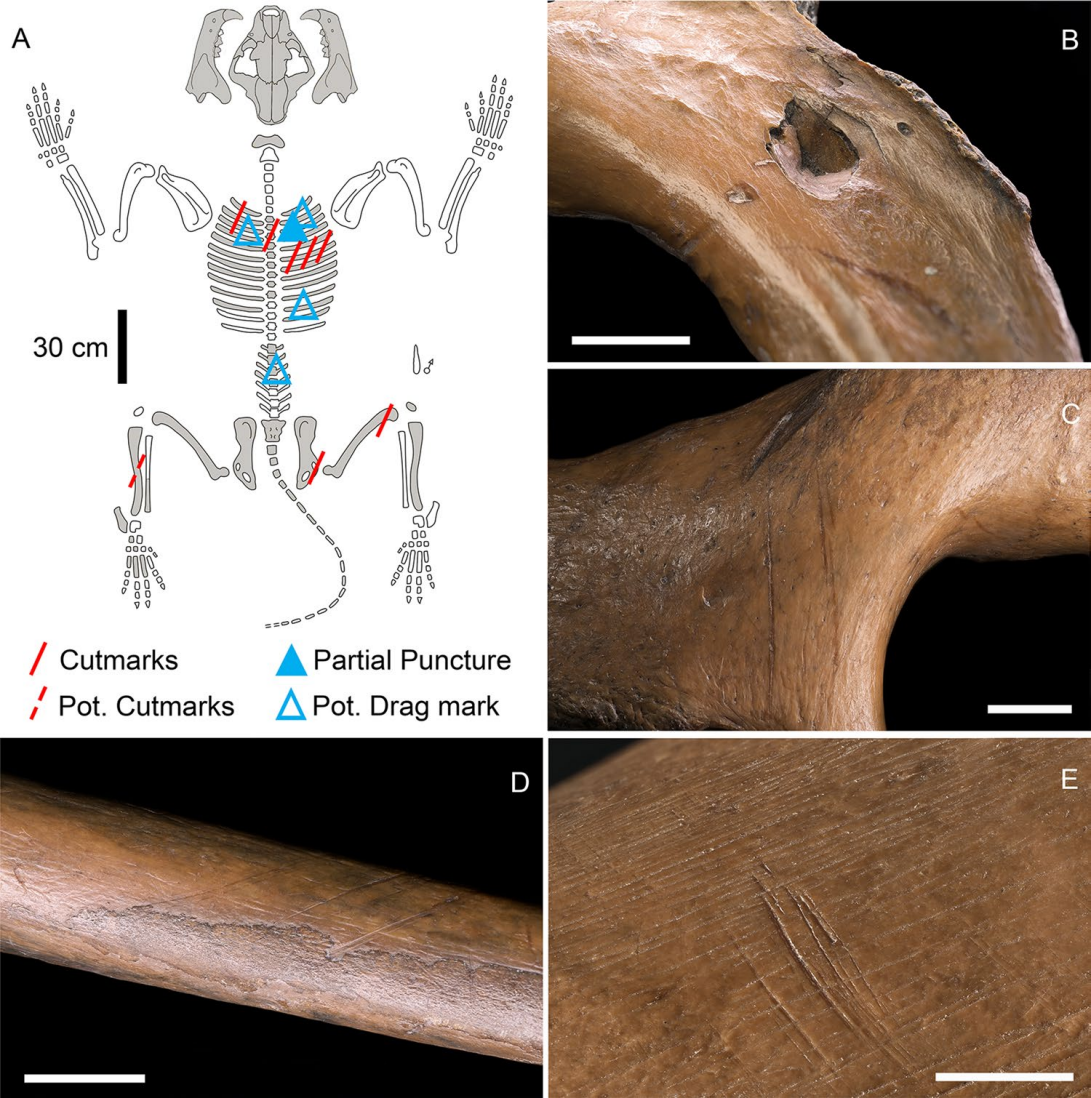
Anthropogenic modifications on the Siegsdorf lion skeleton. (**A)**. Siegsdorf lion skeleton with distribution of observed anthropogenic modifications. Elements highlighted in gray represent those that were originally unearthed. (**B)**. Rib III right ventral view with partial puncture; (**C)**. Right pubic bone with cutmarks; (**D)**. Rib VI right ventral view with cutmarks; (**E)**. Right distal femur caudal view with cutmarks. Scale 1 cm.

The skeletal remains of a cave lion found at Siegsdorf were recovered from a silt deposit associated with a lacustrine environment dating to ca. 48 ka cal BP^52^. Fifty-four of the 62 previously described elements of the partial skeleton (Fig. 2A) (see^51^) were available and re-evaluated for detailed taphonomic analysis. The specimen corresponds to a single male cave lion of medium size and old age, as evidenced by extreme tooth wear and advanced bone remodelling of the extremities and mandible (SI 3.1). The majority of the skeletal elements analyzed are complete and well preserved. None of the bones show any evidence of weathering. The skeleton exhibited three distinct types of surface modification, namely trampling damage, carnivore gnawing, and anthropogenic modifications. Trampling damage is represented by narrow and shallow-bottomed scores that are randomly oriented along the long bones, while carnivore gnawing was localized to only two small areas on the epiphyses of the long bones, specifically the distal left femur and proximal left tibia (Fig. S5). The anthropogenic modifications were further classified into two categories, hunting lesions and butchery marks.

**Figure 3:**
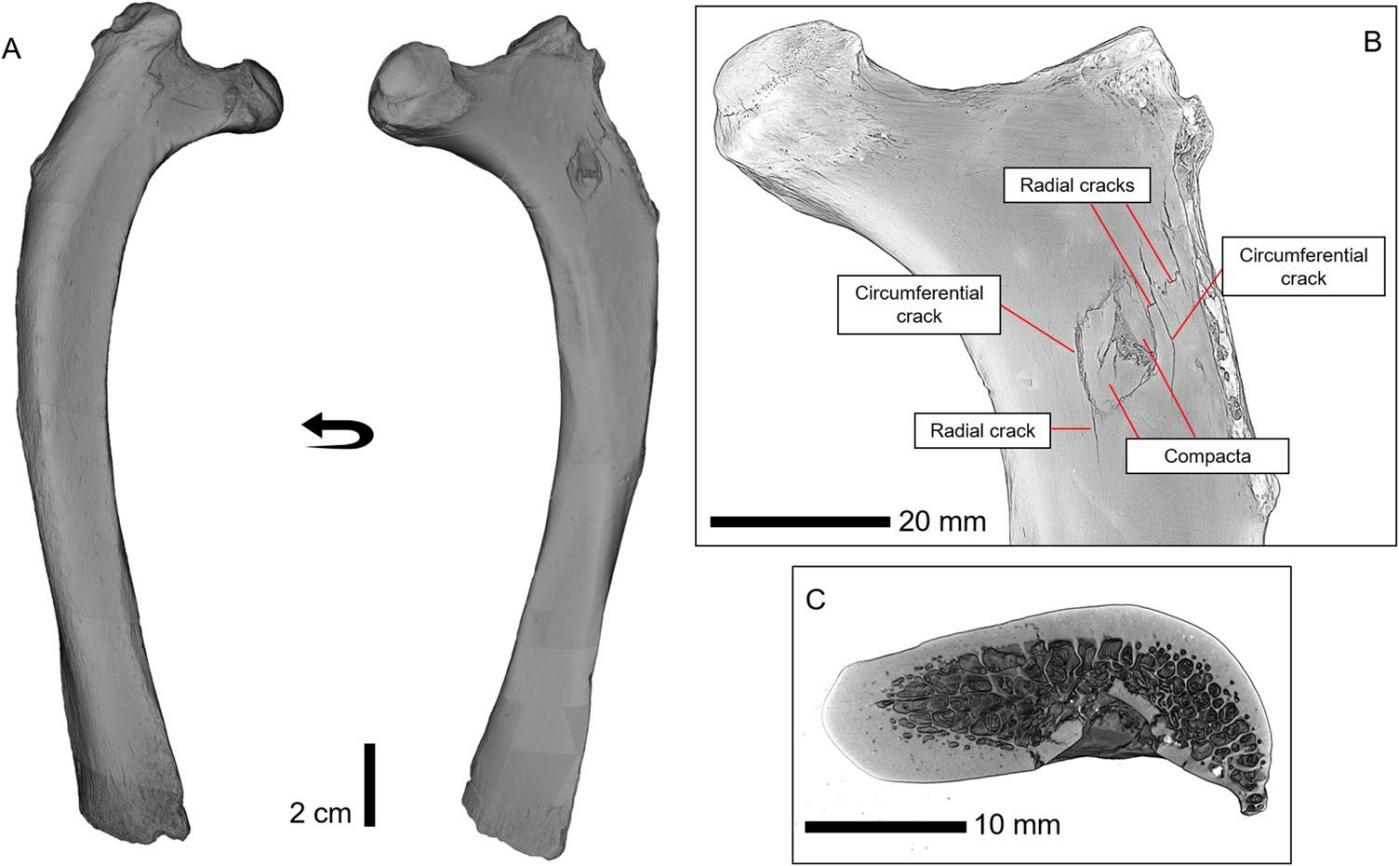
Details of the puncture on Siegdorf's cave lion rib. (**A)**. CT-scan of the right rib III of Siegsdorf cave lion; (**B)**. Close-up of the hunting lesion; (**C)**. Transversal section of the partial puncture.

The hunting lesions include a partial puncture (Fig. 2B) and possible drag marks. According to Duches et al.^53^, a partial puncture occurs when a weapon tip impacts "through bone wall but not all the way through the bone". The partial puncture was observed on the caudal side of the *collum costae* of rib III ID NKM527/7 (Fig. 3). It is oval-shaped in outline, exhibits circumferential and radial cracking on the impact (caudal) side, and lacks an exit wound. The puncture measures 12.6 mm in maximum length and 9.23 mm in maximum breadth at the bone surface, has a volume of ca. 80 mm^3^, and a maximum depth of 4.2 mm. Eccentric outlines of lesions can result from impacts at angles of less than 90º, which is further supported by the ballistic reconstruction (see below;^54^ supplementary information). Further evidence of an angled impact comes from the orientation of the compacted bone fragments in the conical wound channel, which can be seen both in plan and cross-sectional views (Fig. 3).

On the distal part of the lesion, bone fragments (compacta) are compressed on the entry face and into the wound channel, and in its proximal part, the bone surface is also crushed. Overall, these features are characteristic of surface modifications that occurred when the bone tissue was still fresh^55^. Because the puncture was created when the bone was fresh but shows no evidence of tissue healing, the impact must have occurred perimortem. Radial and circumferential cracking and crushing, and bone fragments (compacta) are secondary traits that are demonstrated in the forensic literature^56–58^, and subsequently recognised in trauma on archaeological bone remains to be typical of impact (e.g.,^53,54,59–61^). The primary and secondary traits, as well as the size of the Siegsdorf puncture compare favourably with similar lesions on a cervid vertebra and pelvis from the Neanderthal site Neumark Nord 1, as well as experimental lesions created with wooden spears^54,62^.

A metric analysis comparing this puncture with large carnivore tooth pits and punctures demonstrates that it is larger than widths and breadths of tooth marks (Fig. S13). The Siegsdorf puncture is also noticeably deeper than the tooth damage caused by modern lions or hyenas on large mammal bone elements (Table S16). Additionally, the morphology of the Siegsdorf puncture is not consistent with tooth marks left by large carnivores, which usually produce depressions of the bone surface into the pit^55^. What is more, pits and perforations formed as a result of the pressure of carnivores' teeth on the bone surface often leave bite marks impressions on both sides of the damaged skeletal element. However, there no impression on the rib of the Siegsdorf lion matching the partial puncture. Thus, it is unlikely that a carnivore was the causal agent of the partial puncture. Damage from dynamic loading via hammerstone percussion to access marrow is also ruled out for several reasons. First such damage would be expected to have further similar pits and/or associated or isolated microstriations on the same element and/or other rib or long bone elements^63,64^, but these are absent on the skeleton. Second, experimentally generated hammerstone pits on long bones are substantially smaller in size than this puncture^64^. We therefore tested the hypothesis of an anthropogenic cause of the partial puncture.

We conducted a metric comparison and a linear discriminant analysis (LDA) to better understand how the puncture compares to different types of archaeological and experimental hunting lesions in the literature (see SI 2.3). Metrically, the partial puncture fits well within the groups of lesions produced by wood-tipped weapons and those identified in the archaeological record (Fig. S14). The LDA supports this observation (Fig. S15) classifying the Siegsdorf's case as being produced by a wooden-tipped weapon (Jackknifed classification 45%) (Table S17). The qualitative analysis further supports this hypothesis. Experimental and archaeological data show that stone-tipped weapons often create lenticular or leaf-shaped punctures or perforations^65^, while oval- or circular-shaped punctures, as observed in the Siegsdorf specimen, are consistent with the morphology of wooden spears (e.g.,^54^).

**Figure 4:**
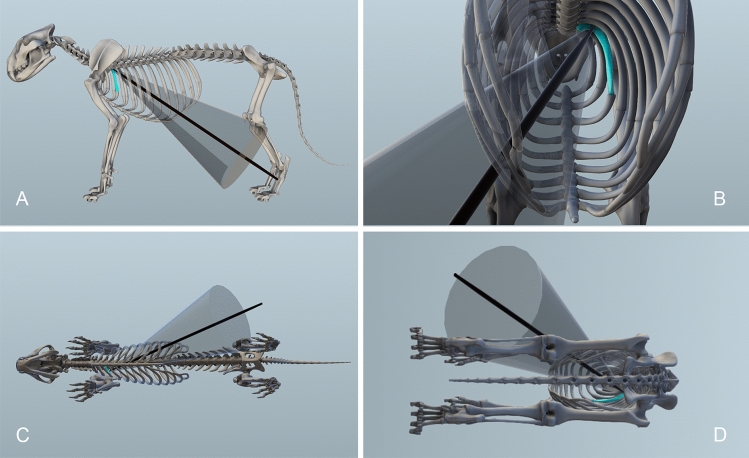
Digital ballistic reconstruction of the Siegsdorf lion spear thrust. (**A)**. standing, lateral view; (**B)**. standing, posterior view of rib cage; (**C)**. lying on right side ventral view; (**D)**. lying, posterior view. 3D digital illustration created with Autodesk Maya 2022.

We tested the plausibility of an impact by a wooden-tipped weapon on the caudal side of the rib by reconstructing the ballistics, including the direction, impact angle (IA) and depth of penetration (DoP) (SI Section. 2.3). The estimated IA in relation to the horizontal plane in a standing position is 29º, while the estimated IA in a recumbent position is -20º (Fig. 4A,D). A negative IA is consistent with projectile impacts and downward thrusts. The standing impact is therefore unlikely because such an angle would be ballistically impossible for a projectile, while thrusting with a spear would require the human to have been thrusting from both beneath and behind the lion. The estimated distance from the ground to the point of entry is ~ 120 cm, which would require a spear that is shorter than known ethnographic spear lengths^62,66^. With the lion in a recumbent position, the IA could have occurred from either a thrust or thrown weapon (Fig. 4C). The DoP is estimated as ~ 66 cm, which is significantly deeper than required for a lethal wound (SI Section.  2.3). Spear thrusting involves significantly higher energies than projected weapons, and more likely to result in such a significant DoP^67^. Wooden weapons are capable of deeper penetration depths compared with stone-tipped weapons^68,69^ and cause hunting lesions on multiple elements, including ribs^54,62,70^. The spear would have entered from the left abdomen through the lion's costal cartilage. Once through the pelt and muscle tissues, the thrust continued through the thoracic cavity, damaging vital organs including the lungs. Organ tissues are significantly lower in density to muscle and bone tissues, and therefore present much less resistance to weapon damage^71^. The spear was then stopped from exiting the lion by impact with the third rib. This would have been a lethal wound for the animal.

**Figure 5:**
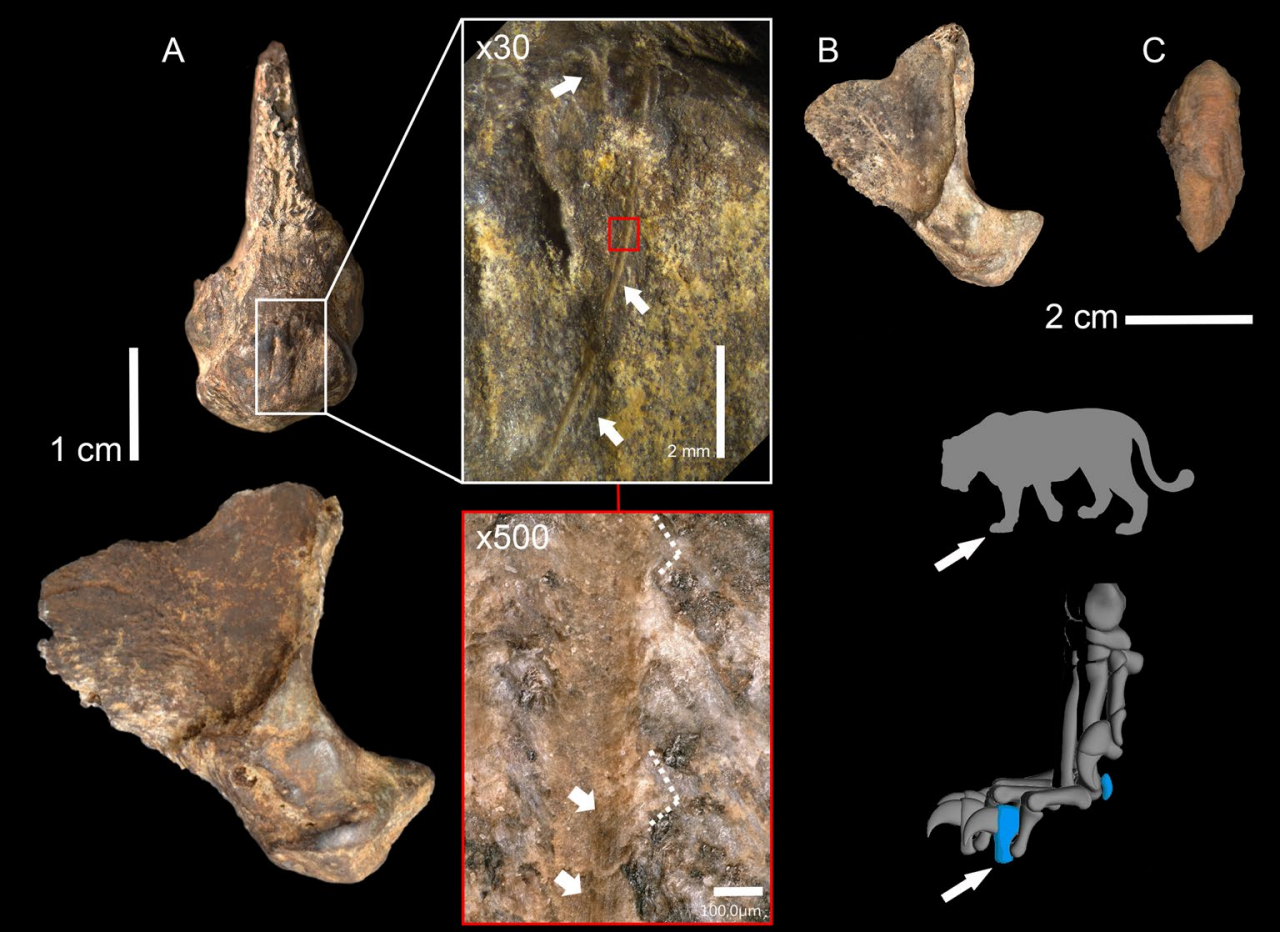
Cave lion remains from Einhornhöhle Area 1. (**A)**. Phalanx III ID 46999448_1384 and close-up view × 30 and × 500 magnifications of the cutmarks; (**B)**. Unmodified Phalanx III; (**C)**. Sesamoid bone. On the right, Illustrations showing the position of the cutmarks (white arrows) and the potential location of the bone elements within the paw of a big cat.

Ethnographically, throwing and thrusting weapons are often used side by side in spear hunting (e.g.,^72^) and it remains possible that potential drag marks may indicate that spears were also thrown at the lion. Drag marks are a type of hunting lesion recorded from the use of stone-tipped weapons and are typically located on ribs and vertebrae (e.g.,^53^ Fig. 5,^59^).

Potential drag marks were observed on three ribs (Fig. S12a, b, S17) and a lumbar vertebra (Fig. S11). The potential drag marks detected here differ from the wide and flat-bottomed drag marks caused by stone-tipped weapons (e.g.,^53^), the morphology of which, however, can considerably vary even if produced by the same type of projectile (e.g.,^53^ Figs. 6, 7;^73^ Fig. 3;^74^ Fig. S5). Only a few studies have investigated damage to rib caused by wooden spears^62,70^. Therefore, the potential for and morphology of drag marks on these elements due to wooden spear impacts remains unclear, and more experimental references would be needed for comparison. Interestingly, the 3D CT-scan of the dragmark on the rib reveals that both cortical and trabecular tissue are bent internally in the damaged area. The tissue fracture exhibits a degree of elasticity of the trabeculae suggesting that the damage occurred peri-mortem, supporting the possibility that the damage is a hunting lesion or a consequence of the butchering process. (Fig. S17).

The butchery marks consist of cutmarks that were observed on the ventral aspect of at least two ribs, a thoracic vertebra, right pubic bone, right femur, and left tibia (Fig. 2A) (S4B). The linear marks are clearly visible at macroscopic level. Overall, the grooves share similar morphology and orientation on the same element. The 3D microscopy analysis of cutmarks on the left pubic bone and the right femur display comparable morphology and size, suggesting that they may have been created by the same tool. The groove on rib III left (ID NKM527/21) shows a congruous morphology with the other cutmarks but with finer measurements as if the lithic implement incised the bone more lightly (SI Section "Significance of the lion skin").

### Cave lion from Einhornhöhle: hide exploitation

Einhornhöhle is a cave site located in the Harz Mountains at the northern fringe of the Central German Uplands. Excavation works deep inside the entrance gallery (Jacob-Friesen Gallery, Area 1) uncovered six well-stratified layers with evidence of hominin occupation during the Middle Paleolithic from at least MIS 6 to MIS 3^44^. The lion remains come from layer H, which is strongly dominated by large carnivore remains that, together with the numerous stone artifacts, probably represent a palimpsest of hominin-carnivore occupations. Based on newly obtained radiocarbon, U-series and combined U-series/ESR age results, layer E2b may be dated to ca. 190 ka. These dating results provide a minimum age constraint for layer H that is stratigraphically located below E2b. Additionally, these new age estimates are stratigraphically consistent with previous radiocarbon and ESR dates obtained from higher layers (Table S2). Besides the lithic assemblage, further evidence of hominin presence at this depth of the cave are the cutmarked bones and a combustion feature^75^. Overall the majority of osseous remains (NISP = 277) display an excellent state of preservation. Weathering and carnivore damage are both rare (see Table S19).

The cave lion assemblage of Area 1 consists of two distal phalanges and a sesamoid that are almost complete and show no significant signs of post-depositional damage (Fig. 5) Both distal phalanges share the same darker discoloration, likely due to manganese in the sediment and were found very close to each other. The sesamoid displays a discoloration of a brighter, almost orange color and is possibly not related to the other two remains. Neither phalanges do not preserve the unguicular hood where the cornified claw sheath was attached. The distinct morphology and size variations in the articular facets strongly indicate different digit positions for the phalanges. In modern lions, each paw typically consists of five phalanx III, and upon closer examination, noticeable differences are observed in the appearance of phalanx III across digits. Therefore, the phalanges from EHH could come from a different digit within the same paw or even from a different paw. The specimens are qualitatively similar in size to modern adult lions.

Two specimens do not exhibit any anthropogenic or carnivore modifications, whereas one distal phalanx (specimen 46999448_1384), exhibits cutmarks on the central-distal and anterior aspect of the flexor tubercle near the tendon attachment (Fig. 5). The longer score measures 4.6 mm in length, has a breadth at the surface of 185 µm, and an average depth of 30 µm. At low magnification, this modification shows a straight trajectory, shouldering at its distal extremity and tapering at both ends (Fig. 5A). It exhibits an asymmetrical cross-section with a morphology ranging from a \_/-shape to a sharp V-shape along the length (Fig. S7). Microscopically, the cutmark is characterized by the occurrence of microsteps and microstriations on the bottom, together with the presence of Hertzian cones at the edge of the groove (Fig. 5A). Two smaller parallel grooves, forming an X-shape striation, were observed near the anterior end of the larger one, on the anterior ridge of the tubercle. These shorter scores are about 0.5 mm in length, with a width of 389 µm and an average depth of 43 µm. Cross-sectional analysis showed that these grooves match the macro- and micromorphological pattern of the larger incision (Fig. S7, S8) although they are somewhat larger in breadth and deeper.

The cut marks on the phalanx are consistent with those generated during skinning of large and medium animals and show distinctive morphology, size, and microfeatures that strongly suggest they were produced by retouched stone tools (see SI 3.1). Moreover, the spatial proximity of the remains, the location of anthropogenic modifications on the elements, and the absence of other types of modifications strongly indicate that the remains were brought into the cave while still embedded in the fur.

## Discussion

### Neanderthal lion hunting

The new finds from Siegsdorf contribute a unique snapshot into the lives of late Middle Paleolithic foragers, and the hunting lesion provides the earliest direct evidence of humans killing and butchering a lion. Ethno-historical records illustrate how modern lions (*P. leo*) can easily kill and prey upon humans (e.g.,^76^ and references therein), and Late Pleistocene lions in their prime were likely similarly difficult to approach. However, present-day African lions spend a significant part of daylight hours resting on their sides or backs, and older male lions are typically nomadic and solitary^77^. Hunters could have taken advantage of the lion in such a resting position, approaching the animal more safely from behind and stabbing it in the lower abdomen while it was lying on its right side. This strategic approach bears resemblance to the tactics employed by Upper Paleolithic hunters during the hunt for cave bears in hibernation. The trajectory of a lithic weapon point embedded in a lumbar vertebra from the Gravettian layers of Hohle Fels clearly illustrates this similarity^11^. Alternatively, the lethal thrust could have occurred after the lion was disadvantaged in some way, potentially by being injured from previous impacts, a strategy also observed amongst contemporary spear hunters^78^.

Wooden spears come from archaeological sites dated from MIS 11 through the Holocene, attributed to different hominin species, including Neanderthals and *H. sapiens* (e.g.,^66,79,80^). Ballistics studies demonstrate that these were likely multifunctional tools that were utilized as both thrusting and throwing spears, as well as for self-defense (e.g.,^54,81^). Ethnographic evidence of recent hunter-gatherers supports the possibility of hunting dangerous terrestrial animals using wooden spears (see^82^). Of particular relevance are accounts of Indigenous South American foragers using wooden thrusting spears for hunting jaguars (*Panthera onca*)^83–85^. Therefore, killing big cats with wooden thrusting spears is within the range of documented human behavior. Today, lion hunting with metal spears still has great importance in Maasai communities in East Africa^86,87^. Although, many human-lion conflicts can be due to acts of retaliation for livestock depredation^86,88^; in the Maasai social structure, cultural/ritual group hunts of lions (*olamayio*) are still practiced to acquire social prestige in the society^87^.

While evidence of the consumption of large carnivores by hominins is archaeologically uncommon compared to ungulate exploitation, we can highlight examples for this practice from the Middle and Late Pleistocene including of bears (e.g.,^11,12,38^) and lions^13,89^. Evidence of bear hunting often indicate that hominins strategically took advantage of hibernation behaviors^11,12^. Large felids such as lions do not hibernate, therefore, hunting them might prove more difficult and dangerous. Obtaining rare or high-risk prey can be understood as a nonverbal signal of the hunters' ability to bear the costs of the hunt^90–93^. As for the Maasai lion hunting, the risks of failure are compensated by the social status achieved^93^. Costly signaling can explain some of the evidence of lion exploitation in the archaeological record (e.g.,^94^), and it should not be ruled out also in the case of the lion from Siegsdorf. However, considering that hunting and gathering is in part opportunistic, the most parsimonious explanation is that the Siegsdorf *P. spelaea* specimen was killed opportunistically and butchered for consumption by Neanderthals. The lion was old and might have been less capable of an effective defense or escape. Interestingly, the butchery pattern indicating consumption fits with the example from the older site of Gran Dolina, Atapuerca (see^13^), supporting the idea that hominids were accustomed to butchering large carnivores, for a remarkably long time. In addition to further evidence of consumption, our analysis demonstrates for the first time that Neanderthals were capable of actively hunting cave lions using simple wooden spears.

### Significance of the lion skin

The absence of polish, wear, perforations, or any distinctive features associated with pendants or clothing components in the lion claws from Einhornhöhle sets them apart from examples found in the archaeological and ethnographic record (e.g.,^95,96^), suggesting their unlikely use as such. In contrast, we propose that the lion phalanges found in the interior of Einhornhöhle offer the earliest evidence of the utilization of a cave lion pelt. The fact that the three lion elements were found in isolation is unlikely to be the outcome of taphonomic processes, such as density-mediated attrition, as a cave bear skeleton that is comparable in size and bone density to that of cave lion is completely represented in the same layer (Fig. S6). Although the potential presence of additional remains in the un-excavated sediment may further inform the findings, the most parsimonious interpretation of the current skeletal representation suggests selective transport into the cave by hominins. The evidence indicates that the carcass was likely skinned elsewhere, and then only the fur, or at least the paws, with the remaining elements left inside (i.e., terminal phalanges with claws) was transported to the site presumably to be used and eventually abandoned^34^. The cutmarks on the distal phalanx result from the tendon slicing process for separating the claw from the second phalanx and the rest of the limb, representing intentional processing of the fur and an advanced anatomical knowledge of the claw. The absence of other cutmarks, including on the other phalanx, may be due to the lack of preservation of the unguicular hood. This anatomical portion of the phalanx can also display anthropogenic modifications related to the claw detachment from the rest of the carcass (e.g.,^21^). Given the absence of other diagnostic remains, definitively determining whether the EHH lion was deliberately killed or found dead by foragers is challenging. While scavenging remains a plausible hypothesis, we argue that the pelt being collected from a fresh kill is more likely. The lack of additional evidence limits our ability to make a conclusive determination. However, considering the sporadic evidence of large carnivore exploitation, including skinning, from earlier periods, intentional killings of such animals were not uncommon (e.g.,^13,16^). Additionally, modern skin processing literature supports the notion that skinning should be conducted while the carcass is still fresh to prevent issues related to carcass deterioration^97,98^.

The exploitation of animal pelts as an environmental buffer has its roots in the Lower Palaeolithic^99,100^, and the archaeological record provides good evidence of the ability of Neanderthals to process hides (e.g.,^101–103^). The furs of large carnivores have excellent thermal insulating properties, making them very useful for coping with colder climates even when used as simple clothing or bedding^100^. However, the evidence of large felids skin removal represented by cutmarks on limb and paw bones are sporadic throughout the Paleolithic (e.g.,^13,17,18,47,50,89,104^), and most of them can only really suggest that the animal was skinned during the butchery process. The only other archaeological example of a cave lion pelt usage, in addition to EHH, is by *H. sapiens*, from the Magdalenian site of La Garma^21^. The distal phalanges of *P. spelaea* from EHH provide the evidence of lion hide exploitation by Neanderthals as early as at least 190 ka. This rare evidence shows that Middle Paleolithic hominins could carefully process large carnivore furs to leave aesthetic elements such as claws in place. Modern taxidermists usually reserve this handling only for valuable skins used as rugs or mounts, thus being considered special trophies^97^.

Evidence of Neanderthals' engagement with other non-human predators is becoming increasingly frequent in the archaeological record (see^43^ and references therein), suggesting that these hominins engaged with animals beyond the typical prey spectrum of ungulates. The presence of two leopard (*Panthera pardus)* paws near articulated skeletal remains of Neanderthals from Sima de las Palomas (MIS 3), in Spain^105^, might be a hint of the cultural role of big cats in their society. It is likely that lions were also culturally meaningful for Middle Paleolithic foragers. The cultural value of the modern lion varies among indigenous communities in Africa, but many groups acknowledge the figure of the large felid as part of their culture^86^. Within the realm of South Africa's traditional medicine (*muthi*), lion parts are highly valued, with the claws being particularly sought-after among healers and traders^106^. In South African Zulu folklore, the big cat represents a metaphor for royalty^107^, while hunter-gatherers of the Kalahari Desert associated the lion with the afterlife^108^. In several indigenous traditions around the world the body elements of large felids such as pelts, claws and canines are worn as symbols of power^109^. Perforated lion canines used by Upper Paleolithic foragers might have played a similar role as a social signaling device. With intact claws, the cave lion pelt from EHH could potentially suggest a connection to the site's use as a residential space. It is worth considering that Middle Pleistocene Neanderthals may have utilized 'persistent places' by adapting and structuring natural spaces with cultural material^110^. Alternatively, the pelt could have been worn for practical purposes, such as warmth or for cultural reasons, while also potentially carrying other social implications linked with the rare acquisition of a large predator fur. Like modern and Upper Paleolithic foragers, Neanderthals probably had variable cultural norms in their interrelationships with lions, and the display of the skin of an important and charismatic species with whom they shared the landscape might have evoked special attention in social and cultural contexts to its owners. We argue that the careful processing and use of EHH cave lion claws represents further evidence of the capacity of Neanderthals to engage with large predators.

## Conclusion

Direct evidence of large predator kills is exceptionally scarce in the archaeological record. Hunting lesions provide some of the clearest evidence that an animal was actively killed by humans, rather than being accessed shortly after a natural death or acquired through confrontational scavenging. The new evidence from Siegsdorf presented here is the earliest instance of cave lion hunting with wooden spears. The continued use of wooden spears whilst Neanderthals were also likely using stone-tipped weaponry is evidenced at sites such as Neumark-Nord^82^ and Lehringen^80^, and therefore their use at Siegsdorf is unsurprising. The cutmarks on several bone elements of the Siegsdorf specimen suggest that the lion was processed at the kill site. After the acquisition of meat and viscera, the carcass was abandoned. The Siegsdorf Neanderthals likely killed a lion in poor condition and exploited the meat for consumption.

More recent archaeological deposits at EHH have already contributed significant evidence for our understanding of Neanderthal cultural behavior^44^. The earliest evidence of lion fur exploitation is the newest addition from the site. The lion remains from inside the gallery testify to the ability to the careful handling of this animal's skin by hominins as early as at least 190 ka. The hide was brought by Neanderthals into the cave potentially for physical comfort, socio-cultural display, or both. Regardless of function, the treatment of lion fur is evidence of this animal's significance to Neanderthal societies.

The remains from Siegsdorf and EHH provide new data on the behavioral repertoire of Middle Paleolithic foragers, adding to the complexity of Neanderthal culture. We conclude that Neanderthals were capable of engaging with non-human predators such as lions not only economically but also culturally – as *Homo sapiens* also is evidenced to have done later in time.

## Material and methods

### Materials

This study includes 54 cave lion skeletal elements from Siegsdorf and three specimens of cave lion (*P. spelaea* Goldfuss, 1810) remains from Einhornhöhle.

The cave lion from Siegsdorf, Bavaria, Germany, was excavated in 1985 by laymen under the directorship of the Bavarian State Collection for Paleontology and Geology. The near-complete lion skeleton is exceptionally well-preserved due to its rapid deposition in the anaerobic sediments of a small lake. A calibrated radiocarbon age of ca. 50 ka obtained from the cave lion's femur agrees well with a directly dated woolly mammoth bone from the same context.

The remains from Einhornhöhle in Lower Saxony, Germany were unearthed during the 2019 excavation season. The three specimens were found some 30 m inside the cave in deposits dating between early MIS 6 and late MIS 7. Detailed information regarding the site, stratigraphy, chronology and material culture associated with the remains are provided in the SI.

All the specimens described in this study were analyzed upon obtaining permission from the appropriate authority/authorities.

### Methods

The taxonomic identification and taphonomic analysis of the faunal assemblages was performed following standard zooarchaeological methods (detailed descriptions SI section "Cave lion from Siegsdorf: killing and carcass butchery"). All specimens were analyzed using 10 × magnification. Bone surface modifications were determined and described according to standard diagnostic parameters established by Domínguez-Rodrigo et al.^111^ and Fernández-Jalvo & Andrews^55^.

Modifications on the specimens from Einhornhöhle were detected during taxonomic identification and initially analyzed using different magnification power (from 10 × up to 80 ×). The specimens 46999448_1384, 46999448_1397, NKM527/7, and NKM527/15 were first subjected to µCT scanning (phoenix V│tome│xm) performed by Waygate Technologies and acquired for 3D morphological analysis. The bone modifications of these four elements were examined alongside specimens’ ID NKM527/1, NKM527/6, NKM527/10, NKM527/18, NKM527/21, and NKM527/36 and quantitatively analyzed by digital 3D microscopy using the Keyence VHX-5000. Additionally, anatomical portions of specimens NKM527/6, NKM527/29, NKM527/31 were µCT scanned with the same resolution at the High-Resolution Computing Tomography Laboratory Eberhard-Karls-Universität Tübingen, for the same purpose. The 3D microscopy imaging was performed at different magnifications from 20 × to 1000 × with the VHX-ZST dual objective zoom lens to obtain panoramic images. Measurements of the surface modifications were obtained following Bello & Soligo^112^, Bello^113^, Bello et al.^114^ and Maté-González et al.^115^. We adapted the same methodology to measure and analyze quantitatively the hunting lesions. Detailed information about the methods used for the 3D surface analysis is described in Section "Cave lion from Einhornhöhle: hide exploitation" of the SI.

The partial puncture on ID NKM527/7 was compared metrically with published data tooth pits and punctures of large carnivores. Comparative metric data for hunting lesions were estimated by extracting metrics from scaled images using Fiji in the following publications of archaeological and experimental impacts (SI Sect. 2.3). The classification analysis (LDA) was performed after checking all assumptions and removing potential outliers (SI Sect. 2.3). Metric analysis and assumption inspection for the LDA were performed with RStudio 2021.09.1–372^116^. Classification analysis was performed in PAST 4.09 software^117^. The associated dataset and code are available as supplementary files.

The 3D reconstruction of the impact involved merging a 3D model of a lion (*P. leo*) with the rib III element CT scan and a schematic wooden spear using the 3D computer graphics application Autodesk Maya 2022. The angle of the spear in the reconstruction conforms to the anatomical position of the rib and the angle of the partial puncture on the rib surface and radial cracking (Fig. 3). Still images of the composite model of the lion in standing and recumbent position were then imported to Fiji to estimate IA and DoP (SI Sect. 2.3).

### Supplementary Information

Supplementary Information.

## Data Availability

The datasets generated and/or analyzed during the current study are available in the Supplementary Information. Supplementary materials not used during the analyses but provided for illustrative purposes can be accessed at the following links: Sketchfab (https://sketchfab.com/denkmalatlas) and Niedersachsen Cultural Heritage Conservation (https://denkmalpflege.niedersachsen.de/live/institution/mediadb/mand_45/psfile/bild/83/Video_digi63f4b6f2e14b8.mp4?1676982524).
