# Author Correction: First direct evidence of lion hunting and the early use of a lion pelt by Neanderthals

**DOI:** 10.1038/s41598-024-52963-y

**Published:** 2024-02-02

**Authors:** Gabriele Russo, Annemieke Milks, Dirk Leder, Tim Koddenberg, Britt M. Starkovich, M. Duval, J.‑X. Zhao, Robert Darga, Wilfried Rosendahl, Thomas Terberger

**Affiliations:** 1 Paleoanthropology, Senckenberg Centre for Human Evolution and Palaeoenvironment, Eberhard Karls University of Tübingen, 72070 Tübingen, Germany; 2 Lower Saxony State Office for Cultural Heritage, Niedersächsisches Landesamt Für Denkmalpflege, 30175 Hanover, Germany; 3 Department of Archaeology, University of Reading, Reading, RG6 6DW UK; 4 Department of Wood Biology and Wood Products, University of Göttingen, 37077 Göttingen, Germany; 5 Senckenberg Centre for Human Evolution and Palaeoenvironment, University of Tübingen, 72070 Tübingen, Germany; 6 Institute for Archaeological Sciences, University of Tübingen, Tübingen, Germany; 7 Centro Nacional de Investigación Sobre La Evolución Humana (CENIEH), 09002 Burgos, Spain; 8 Australian Research Centre for Human Evolution (ARCHE), Griffith University, Nathan, QLD 4111 Australia; 9 Palaeoscience Labs, Department Archaeology and History, La Trobe University, Melbourne Campus, Bundoora, VIC 3086 Australia; 10 Radiogenic Isotope Facility, School of Earth and Environmental Sciences, The University of Queensland, Brisbane, QLD 4072 Australia; 11 Südostbayerisches Naturkunde- Und Mammut-Museum, Siegsdorf, Germany; 12 Reiss-Engelhorn-Museen, Zeughaus C5, 68159 Manssnheim, Germany; 13 Curt-Engelhorn-Center of Archaeometrie, C4,8, 68159 Mannheim, Germany; 14 Seminar of Prehistoric Archaeology, University of Göttingen, 37073 Göttingen, Germany

Correction to: *Scientific Reports* 10.1038/s41598-023-42764-0, published online 12 October 2023

The Supplementary Information file published with this Article contained an error in Table S2. The original Supplementary Information file is provided below.

This error has now been corrected in the Supplementary Information file that accompanies the original Article.

### Supplementary Information

Supplementary Information.

