# Supplementary material for: Author Correction: First direct evidence of lion hunting and the early use of a lion pelt by Neanderthals
Source: Sci Rep. 2024 Feb 2;14:2772. doi: 10.1038/s41598-024-52963-y (PMC10837188; doi:10.1038/s41598-024-52963-y)
Supplement: Supplementary file 1 — Supplementary Information. [file 41598_2024_52963_MOESM1_ESM.pdf]

Supplementary Information for

First direct evidence of lion hunting and the early use of a lion pelt by Neanderthals

Gabriele Russo[1,2]*, Annemieke Milks[3], Dirk Leder[2], Tim Koddenberg[4], Britt M. Starkovich[5,6], M. Duval[7,8,9], J.-x. Zhao[10], Robert Darga[11], Wilfried Rosendahl[12,13], Thomas Terberger[2,14].

[1]Paleoanthropology, Senckenberg Centre for Human Evolution and Palaeoenvironment, Eberhard Karls University of Tübingen, 72070, Germany; [2]Lower Saxony State Office for Cultural Heritage, Niedersächsisches Landesamt für Denkmalpflege, Hanover 30175, Gemany; [3]University of Reading Department of Archaeology, Reading RG6 6DW; [4]Department of Wood Biology and Wood Products, University of Göttingen, Göttingen 37077, Germany; [5]Senckenberg Centre for Human Evolution and Palaeoenvironment, University of Tübingen, Tübingen 72070, Germany; [6]Institute for Archaeological Sciences, University of Tübingen, Germany; [7] Centro Nacional de Investigación sobre la Evolución Humana (CENIEH), Burgos, 09002, Spain; [8]Australian Research Centre for Human Evolution (ARCHE), Griffith University, Nathan, QLD 4111, Australia; [9]Palaeoscience Labs, Dept. Archaeology and History, La Trobe University, Melbourne Campus, Bundoora, 3086, Victoria, Australia; [10]Radiogenic Isotope Facility, School of Earth and Environmental Sciences, The University of Queensland, Brisbane, QLD 4072, Australia; [11]Südostbayerisches Naturkunde- und Mammut-Museum, Siegsdorf, Germany; [12]Reiss-Engelhorn-Museen, Zeughaus C5, 68159 Mannheim, Germany; [13]Curt-Engelhorn-Center of Archaeometrie, C4,8,68159 Mannheim, Germany; [14]Seminar of Prehistoric Archaeology, University of Göttingen, Göttingen, 37073, Germany.

.

Corresponding author: Gabriele Russo

Email: gabriele.russo@uni-tuebingen.de

This PDF includes:

1. Archaeological contexts and Materials: ... 3
   1.1 Siegsdorf ... 3
   1.2 Einhornhöhle (EHH) ... 4
2. Methods ... 5
   2.1 Zooarchaeology and Taphonomy ... 5
   2.2 3D surface analyses ... 6
   2.3 Impact fracture analysis ... 7
   2.4 U-series and combined US-ESR dating of a fossil tooth from Einhornhöhle ... 9
3. Results ... 10
   3.1 Zooarchaeology and Taphonomy ... 10
   3.2 3D surface analyses ... 14
   3.3 Impact fracture analysis ... 15
   3.4 U-series and combined US-ESR dating results on a fossil tooth from Einhornhöhle ... 16
4. Figures SI ... 18
5. Tables SI ... 36
6. References SI ... 53

1. Archaeological contexts and Materials:

1.1 Siegsdorf

The site of Siegsdorf is located at the northern edge of the Alps in the locality of Gerhartsreiter Graben, 1.5 km from the village of Siegsdorf, in south-eastern Germany (Fig. 1). In 1975, Bernard von Bredow, a 16-year-old schoolboy and a friend of his, discovered and partially excavated by hand the remains of a mammoth (*Mammuthus primigenius*).

Exactly ten years later, von Bredow sold the finds to the municipality of Siegsdorf, who thereupon hired him as excavation director to retrieve further finds from that site. Believing that Professor Heißig of the Bavarian State Collection would ensure excavation documentation, he was assigned to supervise the amateur bone-hunter. However, von Bredow did not follow the professor's guidelines and excavated the site with "a group of volunteers". This led Prof. Heißig to leave the excavation to the finder shortly after the fieldwork began (Per. comm. with Heißig, 1990). The cave lion remains were found by chance during agricultural activities in the same year of 1985 after the excavation, financed by the municipality of Siegsdorf, ended. Contrary to all agreements with the local authorities, von Bredow instructed the operator of the excavator to dig deeper into the excavation area. The lion bones were unearthed from this context.

Among the few documentation records is a video clip of the excavation evaluated by one of the authors of this study (R.D.). In the clip, the excavation machine spread the bone containing sediment in front of von Bredow's who is holding a shovel, so that he only had to pick up the bones. Every subsequent attempt to obtain detailed documentation from von Bredow was unsuccessful.
On the one hand, this means that the actual spatial distribution of the skeletal elements is unknown. On the other hand, missing bones like the front limbs have likely remained *in situ*. However, it is also possible that the finder kept the missing bones for himself, as was later the case with other findings.

Analysis of sediments from the 1985 excavation conducted by the Institute of Paleontology at the University of Munich revealed that the skeletal remains were embedded in a deposit of gray silt of an average 0.02 mm granular diameter (coarse mid silt), with small proportions of clay, carbonate stones, and scree. The geologic investigation of the Gerhartsreiter Graben area suggests that these deposits result from a stagnant water pool, fed by a minor tributary creek, which probably served as a watering hole for the late Pleistocene fauna of Siegsdorf[1]. During a new chronostratigraphic investigation of the faunal remains from Siegsdorf, a sample from the lion (KIA 14406) produced a radiocarbon age of ca. 47,600 cal BP[2]. This dating is also consistent with direct dating done on the mammoth and other species found a in similar deposit nearby[1]. The Siegsdorf cave lion remains were first described paleontologically in the doctoral dissertation of Gross in 1992. In her work, Gross assigns all remains to a single individual cave lion (*Panthera spelaea*) and provided detailed metric measurements of all skeletal elements alongside information on age[3]. Her brilliant metric analysis and data are still used in comparative paleontological analyses today (e.g.,[4–6]). Although Gross lacked training in recognizing anthropogenic bone surface modifications, she observed and briefly described some of the cut marks on the Siegsdorf remains, without delivering any information about the taphonomic history of the skeleton.

In 2004, a sample for aDNA analysis was taken from the right femur, the results of which confirmed the taxonomic identification of previous studies[7]. Another specimen was recently extracted from the root of a tooth for further genetic analysis[8]. Although the cave lion from Siegsdorf has been the subject of several scientific investigations, none of these have brought new data concerning the taphonomy of the remains.

1.2 Einhornhöhle (EHH)

Einhornhöhle (or Unicorn Cave) is a dolomitic karst cave located 1.5 km from the village of Scharzfeld, in the southwestern area of the Harz Mountains, Lower Saxony, Germany (Fig. 1). The site is positioned ca. 150 m above the floor of the Oder Valley and about 380 m above sea level, at the geographical coordinates of 51°38′06″N 10°24′15″E. The cave was already known since the 16th century when it was exploited to extract alleged unicorn bones, and to date, it has an extensive history of scientific investigation (see review in[9,10]). In 1985, during a paleontological excavation campaign, the first Middle Paleolithic lithic artifacts were found inside the cave in Area 1 (Fig. S1) of the Jacob-Friesen Gallery (JFG)[11,12]. Since 2014, the Lower Saxony State Office for Cultural Heritage (NLD) has focused its investigations in Area 1, inside the JFG, and Area 4, outside at the JFG entrance. These sectors are located approximately 30 meters apart with a significant descending gradient from the outside to the inside. The lion remains described in this study were found in Area 1 during the 2019 excavation campaign.

Area 1 is located at the far end of the JFG. Its stratigraphic sequence consists of three archaeologically sterile layers (A-C) that yielded mostly micromammal remains and that are deposited on top of six Middle Paleolithic layers (D-I) (Fig. S2). The lion remains from Area 1 (n= 3) come from layer H. The upper sediment unit (Ha) is composed of beige clayey silt atop a thin black manganese band (H(J)) that covers the lower unit (Hb). This lower unit is dark gray due to manganese enrichment, wood ash and charcoal (*Picea abies*, *Larix* sp.). Additionally, heated dolomite stones were recorded here[9,13]. With 273 lithics or 36.9% of the total, layer H is the richest artifact-bearing layer in JFG (Table S1.). The raw material used is of local origin and consists of greywacke, siliceous slate, and rhyolite (Fig. S3). Pebble cortex on some flakes (n= 3) suggests that local river beds were prospected by Neanderthals to collect raw nodules. The lithic assemblage of layer H is made up of chips and chunks (n= 249), flakes and blades (n= 21) and a single Levallois core. Diagnostic Levallois flakes, one Levallois core and one *éclat débordant* confirm the Middle Palaeolithic age of layer H. This is corroborated by the five superimposed layers (D-G) and layer I below layer H that equally contain diagnostic Middle Paleolithic artifacts. 13 artifacts have been retouched into tools, mostly notched/denticulated pieces (n= 6) and side scrapers (n= 3). Interestingly, only layer H has produced side scrapers.

All three lion bones (Fig. 4) come from collected sediments. Both third phalanges (Inv. Nos. 46999448_1397 and 46999448_1384) were retrieved from the same square meter (56/17c) and came from two successive spits at the sediment boundary of units Ha and Hb (Fig. S2). The two phalanges bear a dark brown to black patina staining due to post-depositional manganese enrichment that is typical of many bones in layers D to Ha. The sesamoid bone (Inv. No. 45453992_28) was also retrieved from collected sediments of an adjacent square meter (55/17c) in unit Hb, where it was deposited about 7 cm deeper than the phalanges. This small bone has a dark beige color and lacks the aforementioned manganese enrichment, which is typical of bones from layers Hb and I and clearly distinguishes them from bones of the younger layers D-Ha.

The age estimates of the deposits in JFG are based on seven radiocarbon and three ESR dates (Table S2). Six of eight recently submitted bone samples retained enough collagen to perform radiocarbon dating; only two samples from layer C (Lab-IDs: GrM-22167, GrM-22167, GrM-22170) did not preserve enough collagen. All radiocarbon dates were calibrated using the OxCal 4.4 software and the INtCal20 calibration curve. The single sample from the top of layer A (Lab-ID: GrM-22169) returned a Holocene age of 8,534 +/- 47 cal BP at the 2-sigma confidence interval, indicating that the JFG was filled with sediments during the Atlantic period. A Holocene age is also supported by the microfauna[14]. A single sample from the mixed context of layers A and B (GrM-22168) returned a calibrated minimum age > 47,000 cal BP while four samples from layer B (GrM-

22164, GrM-22165, GrM-22166, GrM-22232) equally returned calibrated minimum ages > 47,000 cal BP at the 2-sigma confidence interval. A previously obtained radiocarbon date on charcoal from layer B1 (atop layer B), at the end of the JFG (Area 3) returned a calibrated age of 45,361 +/-1,200 cal BP and thus corroborated the results[15]. Recently obtained age estimates from the cave entrance (Area 4) and sediment correlations between layer B (Area 1) and layer 4.5 (Area 4) support this chronological position[9]. Radiocarbon dates from layer 4.5 either provided calibrated minimum ages of >47,000 to > 49,000 cal BP, or a semi-finite age between 47,492 and > 55,000 cal BP (KIA-55192). These place units A/B, B1, and B of the JFG in a Marin Isotope Stage (MIS) 3 context.

A single ESR date already produced in the 1980s is available for layer D. The sample (167) was obtained from a cave bear tooth and returned an ESR age ranging from 53 (Early Uptake-EU) to 64 ka (Linear Uptake-LU)[14]. In the first instance, the EU-ESR estimate should be regarded as a minimum age for the tooth. This result should, however, be considered with caution, since the dose rate component from the sediment is based on a sample whose association with the fossil tooth is unclear (Grün, pers. com.). One small ungulate lower molar (excavation ID: EHH2019-1201; lab ID: #597) was collected in layer E2b – just above layer H – in 2019 (see position in Fig. S1 and S2) and was sampled for U-series and ESR dating. Two sediment samples were also taken from the surrounding of the tooth for dose rate evaluation. Methods and results are described below. The newly acquired combined U-series/ESR date from layer E2b yielded a more secure finite age constraint of 189 +16 -14 ka, positioning the deposits to around the MIS 6-7 boundary[16]. The erosional event connected to layer E2a might very well account for the time lapse between layer E1 and E2b. Accordingly, this new dating result provides a minimum age constraint for layer H located stratigraphically below and containing the lion bones, which may then be correlated to MIS 7 or older.

2. Methods

2.1 Zooarchaeology and Taphonomy

The lion remains from EHH were retrieved during water screening. Subsequently, the remains were subjected to mechanical cleaning at the restoration department of the Lower Saxony State Office for Cultural Heritage in Hanover. Mechanical cleaning was done through the use of brushes and wet cotton swabs. During the cleaning process, particular emphasis was applied to the cut marks in an attempt to remove as much sediment as possible from the grooves. Identification of the specimens occurred during the zooarchaeological analysis of the faunal assemblage. The taxonomic identification was performed using the comparative skeletal collection of the Institute of Archaeological Sciences, University of Tübingen, and with the aid of several osteological atlases (e.g.,[17,18]). The NISP (number of identified specimens) was used as a primary counting unit during the data collection as it requires few assumptions, is less susceptible to data aggregation, and is suitable for comparison between layers and assemblages[19–21]. The MNE (Minimum Number of Elements) of cave bear was calculated following Stiner[22] to test density-mediated attrition and derive the minimal animal units (MAU)[23]. The nomenclature of Homberger et al.[24] was adopted for the anatomical description of the distal phalanges.

Taxonomic diversity at Einhornhöhle. The taxonomic diversity was examined to assess shifts in species representation between layers. The species richness is the simple count of the number of identified taxa (NTAXA)[25]. The taxonomic evenness was tested to assess the distribution of taxa in the assemblages by calculating the inverse of Simpson's index[26]. This is expressed as 1/D and is

calculated as $1/\Sigma(\rho i)^2$, where ρ is the proportion of taxon *i* in an assemblage ([20]:196). Higher values of 1/D correspond to an even representation of species.

Bone surface modifications. Taphonomic observations on surface preservation and modification were made using a magnifying glass (mobilux LED x10) and a ZEISS Stemi 305 Greenough stereo microscope magnification range 0.8x 4.0x. The anatomical location of surface modifications was documented for each anatomical element. Anthropogenic and natural modifications were described following diagnostic criteria outlined in Domínguez-Rodrigo et al.[27] and Fernández-Jalvo & Andrews[28].

Bone survivorship and skeletal representation at Einhornhöhle. The effect of density-mediated attrition was assessed by calculating the ratio of teeth to cranial elements[29,30]. The general assumption is that teeth and cranial elements were simultaneously present at the site, so the MNE for bone and tooth is expected to have a ratio of 1:1. High tooth-based MNE ratios indicate that the assemblage was affected by attritional processes ([30]:83-84). The naturally high mortality rate of bears inside caves makes this taxon ideal for testing density-mediated attrition across the vertical stratigraphy of Area 1, as it is likely that their heads were complete at the time of death. In addition, the skeletal representation profiles of cave bear were constructed following Stiner[22] to evaluate the body part representation frequency of large carnivores in the assemblages. However, it is important to note that cave bear remains accumulated naturally, in contrast to the anthropogenically introduced cave lion remains. Consequently, the bones may have undergone different taphonomic processes, and therefore, the obtained results should be interpreted with caution.
The MNE was collapsed in 8 anatomical regions: head excluding teeth, neck, axial skeleton including the pelvis, upper front limbs, lower front limbs, upper hind limbs, lower hind limbs, and feet. Then, the MAU was derived from the observed MNE relative to the expected MNE and plotted. Equal values of MAU across the anatomical regions are expected if skeletal representation is complete.

Weathering. The degree of weathering is a function of its duration and local microclimate; generally, the longer the bones' exposure time from the animal's death to its complete burial, the higher the weathering damage. Weathering degree was recorded adopting the standard stage system from Behrensmeyer[31].

Bone fragmentation. A bone can fracture in several ways depending on the element and its state of freshness, fracture location, and causative agent. In this study, the extent of fragmentation was assessed by tallying the complete specimens in proportion to the total NISP following Lyman ([21]:333-334). The intensity of fragmentation was evaluated for fragmented specimens by calculating their MNE:NISP ratios for each layer (following [32]:151). This method provides standardized values from 0 to 1, in which low values correspond to a higher fragmentation rate.

2.2 3D surface analyses

The specimens 46999448_1384, 46999448_1397, NKM527/7, and NKM527/15 were subjected to µCT scanning to analyze the modifications also under the bone surface. Waygate Technologies GmbH performed this with the aid of a phoenix V|tome|xm µCT scanner. The scanning time was c. 1.25 h for each specimen. The acquired micro-CT data were processed in VGSTUDIO MAX 3.3.4 at the NLD. The 3D images of the right rib III of the Siegsdorf cave lion were obtained using the visualization tools of the Avizo software (FEI, Thermo Fisher Scientific, USA) at the Department of Wood Biology and Wood Products, University of Göttingen. The bone modifications of these four elements were examined alongside specimens ID NKM527/1, NKM527/6, NKM527/10, NKM527/18, NKM527/21, and NKM527/36, by the 3D reflected light microscope Keyence VHX-5000 (Keyence, Neu-Isenburg, Germany) at the Department of Wood Biology and Wood Products

at the University of Göttingen. This microscope enables non-destructive and high depth-of-field images in 2D and 3D. The imaging was performed at a magnification range from 20× to 1000× with the VHX-ZST dual objective zoom lens. Thereby, single images or panoramic images were captured. Panoramic images result from capturing multiple single images that are stitched by the microscope software.

For topographic 3D images, the observation field's lowest and highest focus planes were manually determined. Then, the microscope computes a high depth-of-field image of the observation field using automatically acquired images at different focal planes in the z-direction (i.e., height). The result is a 3D representation of the observation field, revealing the topographic profile. The color mapping of such 3D images indicates the height within the 3D representation. Thereby, blue codes the lowest focus point, whereas red designates the highest focus point. Apart from imaging, the cut marks were quantified through automated profile measurements. The morphometric analysis of the surface modifications was accomplished based on topographic 3D images. The images were captured by the 3D reflected light microscope and the images were examined with measuring tools in the microscope software. The cross-sectional profiles reveal the topography and morphology of the cut marks. A straight line perpendicular or parallel to the surface modifications was realized to measure the bone damage (e.g., breadth, depth, length) following the guidelines outlined by Bello & colleagues[33–35], and Maté-González et al.[36–38]. The opening angles were analyzed in the image analysis software Fiji[39]. The averaged values were presented with the min and max values in parentheses. We have adapted this methodology to quantitatively measure the partial perforation. The puncture's width at the surface and depth were determined by two cross-sectional profiles perpendicular to each other. The volume of the lesion was estimated from the bottom of the puncture to the opening on the bone surface by the microscope software based on the 3D images.

2.3 Impact fracture analysis

Several taphonomic agents can create cone-shape perforations in bones; however, most of these only produce small and shallow marks[28]. Large carnivores and humans both have the ability to produce wounds that can penetrate deeply into the bone surface. While recent advances in technology and methods have made identifying cut marks and bitemarks easier, identifying the agent responsible for deep, cone-shaped perforations on bones remains difficult. This issue was addressed by adopting both a qualitative assessment of the potential hunting lesions, alongside a metric comparison.

Qualitatively, the partial puncture can be compared with experimental and archaeological partial punctures. Images of partial punctures with or without embedded tip material (see [40–44]) show that the outline morphology of these types of hunting lesions tend to follow that of the weapon point. As current evidence stands, Neanderthal weaponry is confined to wooden spears and spears hafted with stone points, suggesting that one of these two types of weapons caused the hunting lesion.

Metric comparison is widely used in zooarcheology for the taxonomic identification of agents causing pits and punctures in bone assemblages (e.g.,[45–48]). This method utilizes linear data that are easily accessible, processable, and comparable. However, such approaches can be problematic in attempts to definitively distinguish between different carnivore taxa or even carnivore size categories. This is because a myriad of factors, including bone condition, presence/absence of soft tissue, bone element, and carnivore size and family (e.g., canid or felid) can influence the size, morphology and patterns of the bite marks (see [28,48]). Nevertheless, metric analysis has previously been employed in the developing field of the identification and analysis of hunting lesions[43,44] to explore metric differences between weapon damage from carnivore tooth marks. We further develop this approach here, through assembling additional published and unpublished metric data on the maximum and minimum length and width of pits and punctures of large

carnivores from actualistic studies, and metric data on hunting lesions from experimental and archaeological investigations.

As the actualistic literature provides a vast amount of data on tooth marks left by different taxa, we have selected data published in the form of descriptive statistics concerning tooth pits and punctures from large modern carnivores relative to those that roamed central Europe during the Late Pleistocene, namely wolf, bear, lion, and hyena. From these data, pits and puncture measurements were extracted from specific anatomical portions of different elements that have a bone density comparable to that of a medium to large mammalian rib. The summary of the linear measurements used is shown in Table S3. Published metric measurements of hunting lesions were more limited. To maximize the power of a quantitative analysis, raw data were collected from published images of experimental and archaeological hunting lesions. Archaeological hunting lesions are an under-recognised phenomenon[44,49], and relatively few experiments providing reference material have been conducted. Furthermore, existing publications have rarely included metric data for either archaeological or experimental lesions. Measurement data were collected from published scaled images of punctures and perforations, regardless of the anatomical portion or taxa, and inclusive of different weapon technologies. This metric analysis is primarily to follow previous work illustrating a clear metric separation in size between hunting lesions and carnivore tooth damage (see also [43,44]). Where entry and exit punctures and perforations of lesions were both illustrated the entry wound was selected for analysis. Using the image analysis software Fiji[39] the scale was set according to the photographic scale, then the measurement tool was used to estimate the length and breadth in mm of perforations and punctures, using the undamaged bone surface. The hunting lesion data and sources are in Table S4.

The tooth pit and hunting lesion data sets were statistically visually compared to the partial puncture measurements on specimen NKM527/7 using scatterplots with 95% confidence intervals calculated using the standard deviations on RStudio 2021.09.1- 372 software[50]. We conducted a Linear Discriminant Analysis (LDA) to test whether there would be demonstrable quantitative differences between different weapon points. As this approach is in its methodological infancy, we underscore that the results exploring different weapon impacts are to be treated with caution and represent only an additional means of assessing hunting lesions. New developments making use of artificial intelligence are a promising additional method which could be developed to further distinguish between carnivores and hunting lesions, and in theory would also hold potential for the identification of different weapon types (e.g.,[51]).

The LDA was conducted with PAST 4.09 software[52] using the hunting lesion dataset. The Siegsdorf lesion was treated as unknown against three defined groups, namely "Wooden Spear" (experimentally produced hunting lesions with wooden spears), "Composite Weapons" (experimentally produced hunting lesions with hafted lithic or osseous points), and "Archaeological Lesions" (hunting lesions identified in the archaeological record). We verified that the variables showed an approximately normal distribution on the basis of both histograms and box plots. When we checked for outliers we found that the hunting lesion from Noe-Nygaard[53] Plate 1d is an outlier that strongly affects normal distribution and variance of the data, potentially affecting the reliability of the analysis, and was therefore excluded. The covariance of the matrices was homogeneous for the sample used, as shown by Box's *M*-test (resulting *P* values = 0.06). When all assumptions were met, we ran the analysis.

Reconstructing the ballistics of Palaeolithic hunting lesions was facilitated merging a 3D model of a lion (*P. leo*) with the rib III fragment and a schematic wooden spear using the 3D computer graphics application Autodesk Maya 2022. The schematic spear was fitted into the partial puncture in accordance with the angle of the wound channel and radial cracking. The trajectory of the spear thus followed the lesion characteristics.

Impact angle (IA) is an important ballistic variable (e.g.,[54,55]), and its reconstruction can assist in evaluating potential delivery systems (e.g.,[44]. The IA was estimated in accordance with the horizontal plane (see also [56]), providing a fixed reference point to assess the trajectory irrespective of the positioning of the lion at time of impact. The 3D model of the lion skeleton with the schematic spear was positioned as standing and lying on its right side in MeshLab 2022.02 application using a gridding background. Still frames were then imported to Fiji, and impact angles of the spear for the two positions were estimated using the angle tool.

Depth of Penetration (DoP) is a further measure used in ballistics studies (e.g.,[57,58]). A depth of ca. 20 cm generally considered necessary for a fatal wound, with the qualification that this estimate is based upon the human body, and logically would vary considerably based on an animal's physiology and overall size as well as location of impact. An estimate of the DoP was again facilitated by the use of the 3D skeletal model and schematic spear in the same way as IA was calculated, using the published measurement of the shoulder height of the Siegsdorf lion as 1.2 m.

2.4 U-series and combined US-ESR dating of a fossil tooth from Einhornhöhle

Tooth location. Tooth #597 was collected from stratigraphic layer E2b in the area 1 of the JFG (Figs. S1 and S2).

Sample preparation. Tooth #597 was prepared as follows: the enamel layer was mechanically separated from the other dental tissues and both inner and outer surfaces were removed with a dentist drill to eliminate the volume that received an external alpha dose. The dentine attached to the enamel layer was kept aside for subsequent solution bulk U-series analyses. All dental tissues were ground and sieved <200 µm.

ESR dose evaluation. Dose evaluation utilized the multiple aliquot additive dose (MAAD) method. The enamel powder of each sample was split into ten aliquots. Ten of them were gamma irradiated at CENIEH (Spain) with a Gammacell 1000 Cs-137 gamma source (dose rate = 6.13 ± 0.14 Gy/min) to the following doses: 49.4, 98.9, 148.3, 197.6, 296.4, 395.3, 592.9, 889.5 and 1482.4 Gy. One aliquot was kept unirradiated (= naturel aliquot).

Room temperature ESR measurements were carried out at CENIEH with an EMXmicro 6/1 Bruker ESR spectrometer coupled to a standard rectangular ER 4102ST cavity. The following procedure was used to minimize the analytical uncertainties: (i) all aliquots of a given sample were carefully weighted into their corresponding tubes and a variation of <1 mg was tolerated between aliquots; (ii) ESR measurements were performed using a Teflon sample tube holder inserted from the bottom of the cavity to ensure that the vertical position of the tubes remains exactly the same for all aliquots. The following acquisition parameters were used: 1-25 scans, 1 mW microwave power, 1024 points resolution, 15 mT sweep width, 100 kHz modulation frequency, 0.1 mT modulation amplitude, 20 ms conversion time and 5 ms time constant. All aliquots of a given sample were measured within a short time interval (<1 h). This procedure was repeated three times over successive days without removing the enamel from the ESR tubes between measurements in order to evaluate measurement and equivalent dose (D_E) precisions (Table S5).

The ESR intensities were extracted from T1-B2 peak-to-peak amplitudes of the ESR signal[59], and then normalized to the corresponding number of scans and aliquot mass. DE values were obtained by fitting a single saturating exponential (SSE) through the mean ESR intensities derived from the repeated measurements. Fitting was performed with Microcal OriginPro 9.1 software, which is based on a Levenberg-Marquardt algorithm by chi-square minimization. Data were weighted by the inverse of the squared ESR intensity ($1/I^2$) and the inverse of the squared experimental errors ($1/s^2$). ESR dose response curves (DRCs) are shown in Fig. S4. Final 1-σ D_E error used for age calculation include both the fitting and gamma source dose rate errors (2.3%).

Solution U-series analyses by MC-ICPMS. Solution U-series analyses of powdered enamel and dentine were carried out using a Nu Plasma HR MC-ICP-MS in the Radiogenic Isotope Facility (RIF) at the School of Earth and Environmental Sciences, the University of Queensland (Australia), following chemical treatment procedures and MC-ICP-MS analytical protocols described elsewhere (e.g.,[60,61]) Powdered sub-samples weighing 1–5 mg were spiked with a mixed ^{229}Th-^{233}U tracer and then completely dissolved in concentrated HNO_3. After digestion, each sample was treated with H_2O_2 to decompose trace amounts of organic matters and to facilitate complete sample-tracer homogenization. U and Th were separated using conventional anion-exchange column chemistry using Bio-Rad AG 1-X8 resin. After stripping off the matrix from the column using double-distilled 7N HNO_3 as eluent, 3 ml of a 2% HNO_3 solution mixed with trace amount of HF was used to elute both U and Th into a 3.5-ml pre-cleaned test tube. After column chemistry, the U-Th mixed solution was injected into the MC-ICP-MS through a DSN-100 desolvation nebulizer system with an uptake rate of around 0.07 ml per minute. U-Th isotopic ratio measurement was performed on the MC-ICP-MS using a detector configuration to allow simultaneous measurements of both U and Th. Closed-system U-series ages were calculated using the Isoplot/Ex 3.75 Program[62]. Analytical results are given in Table S6.

Dose rate evaluation and age calculations. No in situ evaluation of the gamma dose rate associated to the tooth was performed. Consequently, both the beta and gamma dose rates analysis of the two bulk samples extracted from the surrounding sediment were derived from the laboratory. Inductively Coupled Plasma Mass Spectrometry (ICP-MS) analyses were performed by Genalysis Laboratory Services, following a four-acid digest preparation procedure. Radioelement concentrations (U, Th and K) are given in Table S7.

The following parameters were used for the dose rate calculations: an alpha efficiency of 0.13 ± 0.02[63], Monte-Carlo beta attenuation factors from Marsh[64], dose-rate conversion factors from Guérin et al.[65], and an assumed long-term water content value (% wet weight) of 0%, 5 ± 3 % and 20 ± 5 % in enamel, dentine and sediment, respectively. Post-Rn equilibrium was assumed in dental tissues and sediment. A cosmic dose rate of 40 µGy/a was considered based on an estimated depth of 15 ± 1 m, by taking into account the distance of the fossil specimen from the entrance (about 30 m) and the estimated thickness of the overburden cave sediment (~1.8 m), cave roof (~12.5 m) and sediment/soil cover (~1.25 m). Beta dose rate evaluation was performed using a tooth geometry sediment/enamel/dentine.

Age calculations were performed with DATA[66], a DOS-based program using the US and CSUS models defined by Grün et al.[67] and Grün[68], respectively. The CSUS model is based on the assumption that all of the uranium migrated into the sample at a time given by the closed system U-series age. The CSUS-ESR age is the maximum age that can be derived from a given U-series and ESR data set. Age calculations using the US and CSUS models encompass all possible uptake scenarios. Data inputs and outputs are given in Table S8. Age results are given at 1-σ confidence level.

3. Results

3.1 Zooarchaeology and Taphonomy

Siegsdorf. In light of the circumstances of the find, there is no direct information about the environmental background of the Siegsdorf cave lion. However, a faunal assemblage consistent with the Mammoth Steppe biome was unearthed in the same area and from a similar deposit[69]. The remains of the large carnivore were analyzed for the first time by Carin Gross for her doctoral thesis in 1992. It is a remarkably well-preserved and nearly complete skeleton often used in

comparative studies and, for this reason, is well known in Paleontology. In her thesis, Gross describes 62 elements, including a complete cranium and mandible with all teeth except the lower left third premolar, a total of 21 vertebrae, 23 ribs, entire pelvic girdle, and lower limb bones, including the keratin sheath of a claw[3]. In our study, 54 of the previously described elements were revisited (Fig. S4A), as the left foot bones (i.e., astragalus, calcaneus, three metatarsals, two *os tarsi*, and the keratin sheath) were not available for analysis.

Most elements are complete, except for the left distal fibula and the extremities of some vertebrae' spinous processes that exhibit modern fractures. The transverse fracture morphology and lighter coloration of the fracture outline on the incomplete elements speak to a modern fracture, probably due to the excavation methods (see above). The favorable preservation of the remains allowed for a thorough inspection of the bone surface and its modifications. A total of at least five bones exhibited clear evidence of anthropogenic modifications associated with carcass butchering. Other hominin-induced modifications relate to the individual's cause of death are referred to as hunting lesions and were detected for the first time during the present study. Hunting lesions described here are a partial puncture and potential drag marks (see [43,70]).

- Left rib III ID NKM527/21. Oblique cut marks were observed on the ventral side of the *corpus costae*, and a possible drag mark is located on the *collum costae* (Fig. S12A).
- Right rib II ID NKM527/6. A potential drag mark was recorded on the lateral side of the *collum costae* (Fig. S12B, S16).
- Right rib III ID NKM527/7. A partial puncture was observed on the caudal side of the *collum costae* (Fig. 3) (described in detail in SI 3.2). The shaft also displays some trampling.
- Right rib VI ID NKM527/10. This specimen displays several oblique cut marks along the ventral side of the *corpus costae* (Fig. 2D). The bone also exhibits trampling (or bird beak/talon damage).
- Right rib XI ID NKM527/15. A possible drag mark was recorded on the lateral side of the neck.
- Thoracic vertebra III or IV ID NKM527/45. This specimen shows oblique cut marks on the left side of the spinal process.
- Lumbar vertebra III ID NKM527/36. A notch on this specimen on the sinistral side of the spine process may be an anthropogenic drag mark (Fig. S4C).
- Innominate ID NKM527/18. Two long and parallel cut marks were observed on the lateral aspect of the right pubic bone (immediately underneath the acetabulum) (Fig. 2C). Other linear marks on the specimen are due to trampling.
- Right femur ID NKM527/1. On the distal-posterior view of this element, multiple cut marks transverse to the main axis are well visible. More linear marks on the proximal-lateral aspect and on the shaft are due to trampling (Fig. 2E).
- Left tibia ID NKM257/31. Potential cut marks parallel to the main axes were observed; other linear marks on the shaft are clearly from trampling.

Some of the skeletal elements bearing anthropogenic surface modification are shown in Fig. S4B.

Overall, anthropogenically made cut marks are well distinguishable from the trampling damage and are coherent across the skeleton; these linear marks all present a similar morphology (e.g., size, internal microstriations, orientation) and are oriented parallel to one another on a single element or within a single field of cuts.

Trampling damage can be observed particularly on long bones, and is represented by small and narrow shallow-bottom linear marks that are sometimes barely visible to the naked eye and with random orientation on the same element (Fig. S16). These scratches due to contact between sediment and the bone surface can easily be mistaken for anthropogenic cut marks as Gross did for the scores described on the right rib IV ([3]:70). Another type of linear mark observed on the

bones are wide and deep-bottomed grooves with internal microstriations. These scores are generally associated with trampling caused by friction between a coarse substrate and the osseous surface. However, the activity of scavenging birds such as vultures has been documented to cause striae very similar to this description[71], so their taphonomic agency should not be ruled out. This type of modification is observed, for example, on the posterior view of the midshaft of the right tibia and the latero-proximal view of the left femur (Fig. S5A,B). Finally, carnivore damage is only observable on the medial condyle of the distal epiphysis of the left femur (Fig. S5A,B,C) and on the latero-frontal aspect of the proximal epiphysis of the left tibia (Fig. S5E,F). This type of damage was just briefly mentioned in previous publications[72], and therefore we provide a detailed description of it here.

Punctures and pits found on the medial condyle of the femur and lateral aspect of the tibial tuberosity display crack-like edges with micro-crushing and a ragged edge with irregular morphology. The thin cortical bone is compressed inside the trabecular tissue. This damage corresponds to the negative impression of carnivore tooth pits, and it is associated with abrasion damage that can also result from gnawing. Similarly, the extensive crushing present on the distal epiphysis of the left femur display with irregular, ragged edge morphology and, therefore, is likely the result of the same process. Interestingly, the 3D μCT scan shows that the trabecular tissue around these lesions is not compressed but simply missing (Fig S18). It is also important to note that the deep lesion on the left aspect of the femoral condyle could not have been created before the complete disarticulation of the limb. Overall, all the evidence suggests that the remains were already dry when the carnivore damaged these elements.

The three wide U-shaped grooves on the medial condyle of the femur and several pits on the anterior tibial tuberosity present a morphology comparable to carnivore bite marks. This damage has much darker discoloration. This discoloration is absent in surface modifications that have occurred perimortem, such as cut marks or hunting lesions, but similar to the trampling marks examined by 3D microscopy in Figure S12C. Unexpectedly, the μCT scans revealed radio-opacity around these lesions (Fig. S18). In radiology, the radio-opacity of bone tissue can be due to several causes, most of which are attributable only to fresh tissue[73]. In our case, the radio-opacity can be explained mainly by the calcification of the bone tissue due to pathology or by the presence of metallic material, like metallic minerals, in the damaged areas. We ruled out the pathology hypothesis because of the precise concentration of the radio-opacity around the bone damage. We instead consider more plausible the possibility that naturally occurring metal minerals in the sediment infiltrated the trabecular tissue in correspondence with carnivore damage after this happened.

As suggested by Gross, given the severe condition of dental wear, it is very likely that the skeleton belonged to a very old individual. The advanced age of the animal would also account for the abnormal growth of bone tissue present on some skeletal element extremities and the mandible, often related to pathologies common in old animals that were already observed by Schouwenburg et al.[72]. Metric comparison of previous studies with other European specimens concluded that the Siegsdorf cave lion was a medium-sized male individual[6,72].

Einhornhöhle. The faunal assemblage of layer H (NISP = 277) is the richest at the site and is composed mainly of large carnivore remains (81.95%, NTAXA = 3) (Table S9). The taxonomic diversity of the assemblage is highly uneven (1/D = 1.05) as cave bear (*Ursus cf. spelaeus*) is overrepresented (79.78%), while cave lion is rare in comparison (NISP = 3, 1.08%). Anthropogenic modifications were observed on one cave lion phalanx (0.36% of NISP). Carnivore damage is rare (11.19%) and consists mainly of bite marks and crenulations. Overall, the bone surface

preservation is excellent. Thirteen percent of the remains suffered from weathering, which primarily caused open linear cracks on the cortical surface. Complete bones in layer H are uncommon (32%); however, the fragmentation index shows a moderate fragmentation intensity (0.49).

The test for bone survivorship in layer H illustrates that teeth are more frequent than bones, suggesting that this horizon might have suffered from density-mediated attrition (Table S10). However, the cave bear anatomical profile displays an even representation of the skeletal portions of this taxon in this deposit (Fig. S6). The available evidence indicates that density-mediated attrition likely have contributed to the disappearance of some remains from the fossil record, but it appears that such effects were not significant enough to result in the complete obliteration of a large carnivore skeleton, with the exception of only a few small foot elements.

The cave lion sample consists of two third phalanges and one sesamoid that likely belong to an adult individual. All three elements are well-preserved and show no evidence of carnivore or post-depositional modifications. Because it is not easy to identify with certainty the anatomical position of these elements within the same skeleton (i.e., digit, lateralization, anterior or posterior extremity), and given the significant variability in their size depending on this, it was not possible to perform biometric comparisons to infer the size and sex of the individual. However, given the size of the phalanges are comparatively similar to those of a modern adult lion, it is reasonable to assume that the archaeological specimens did not belong to an infant or juvenile individual at the age of death. The summary of recorded measurements is given in Table S11.

- Phalanx specimen 46999448_1384. This phalanx does not preserve the unguicular hood (Fig. 5A). Cut marks are observed on the central and distal part of the flexor tubercle.
- Phalanx specimen 46999448_1397. This specimen is slightly larger and has a distinct articular facet morphology than the other phalanx, indicating a different anatomical location in the paw (Fig. 5B). It does not preserve the unguicular hood, and no modifications have been detected.
- Sesamoid specimen 45453992_28. This bone is in an excellent state of preservation and does not show any modification (Fig. 5C).

The cut marks on phalanx III exhibit a straight trajectory, flat-bottom (_/-shape) to acute angle (V-shape) morphology, shouldering, asymmetric cross-section, presence of Hertzian cones, and microstriations along the bottom of the groove (Fig. 5A, Fig. S7, S8). However, it should be noted that the cleaning process used to remove sediment from the cut marks may have altered some of their qualitative features, such as microstriations, edge flaking, and coloration, which should be considered when analyzing and interpreting the marks[74].

Given the absence of similar linear marks on any other specimens from the H layer, it is improbable that they were caused by trampling, as a much higher frequency of the marks would be expected across the specimens in this case. Instead, the features described above are associated with striae produced by retouched stone tools[28,75–77]. In addition to the type of tool used (e.g., retouched vs. unretouched, flake vs. handaxe), the size and presence of certain morphological characteristics of the linear marks may also vary according to the coarseness of the lithic raw material as has been established through experimental studies[36]. The raw materials used at Einhornhöhle largely consist of locally available stone such as siliceous slate, hornfels, greywacke, and quartzite[9]. The flakes were retouched and made into tools, while handaxes and other bifacial tools were absent. Considering the morphological characteristics of the cut marks and the archaeological context in which the phalanx was found, we hypothesize that the damage was probably created using a retouched flake.

3.2 3D surface analyses

The micromorphometric values of the cut marks on the Einhornhöhle lion phalanx III are summarized in table S20. At least two groups of cut marks have been identified on the phalanx III specimen 46999448_1384 from EHH. In total, the larger cut mark has a length of 4.6 mm. Microscopically, this modification appeared to be interrupted by dividing it into two segments, as shown in Fig. S7A, B. The cross-sections reveal a morphology ranging from _/-shape to V-shape with an asymmetrical profile at lower magnification (Fig. S7B). The depressions of the cut mark could be clearly seen, especially at a higher magnification of 1000× (Fig. 7C). Composite images at 1000× magnification, as exemplified in Fig. S7C and Fig. S8B, depict a narrow _/-shaped profile featured with local ancillary grooves or ridges. The bottom of that _/-shaped profile is on average 65 µm (min-max 42-87 µm). Morphometric data of the cut mark also indicated that the breadth and the depth marginally varied along the length. Measurements showed a breadth at the surface of 185 µm (min-max: 122-227 µm) and a depth of 30 µm (min-max: 21-40 µm). The estimated opening angle is 100° (min-max: 76°-129°) for the cut mark.

The second set of cut marks is located on the anterior ridge of the tubercle, near the distal end of the larger cut mark described above (Fig. S7D, Fig. S8A). This consists of two parallel grooves of similar size that appear to form an X-shape. These smaller incisions were not connected with the other cut mark (Fig. S8A). However, cross-sectional analyses of the 3D models reflected a profile pattern (_/-shaped or V-shaped) similar to the previous cut mark exhibited in Fig. S7B. The breadth is 389 µm (min-max: 317-437 µm), and a depth is 43 µm (min-max: 37-50 µm). The opening angle is 99° (min-max: 72°-106°). The incision visible in the tomographic images of the bone appears to be a crack on the bone's surface as the microscopy image in Fig. S7E demonstrates.

For the 3D microscopic analysis of cut marks on the Siegsdorf lion, three elements were selected to compare the internal morphology of anthropogenic modifications on different anatomical portions of the same skeleton. The sample includes cut marks on the right distal femur, right pubic bone, and shaft of left rib III. Raw morphometric values are shown in Table S13. The cross-section analysis of the cut marks on the femur revealed a clear V-shape and the asymmetrical morphology of the scores (Fig. S9). The cut marks were well distinguishable from the natural U-shaped bone grooves, which run perpendicular to the cut marks, from the view of the profile lines (Fig. S9A). The morphometric analysis obtained an average depth of 26 µm width of 273 µm. Similar observations were made on the pelvic bone. Apart from single V-shaped cut marks, "two-lane" cut marks were also visible (Fig. S9B). The measured depth ranged from 20 to 45 µm. The width at the bone surface was 338 µm on average. Cut mark features were also found on the left rib III with the typical V-shape (Fig. S9C). The morphometric analysis of the grooves obtained a width at the bone surface averaging 87 µm and a depth of 7 µm.

It should be noted that overall the dimensions of Einhornhöhle's morphometric features (Table S20) compare well with those of Siegsdorf (Table S15). In general, the morphometric values of the cut marks on Einhornhöhle's phalanx are lower than the average of the cut marks associated with the skinning of large and medium animals reported by Bello et al.[78] and Wallduck and Bello[79] but are nevertheless well within the minimum limits of this category.

Hunting lesions were also analyzed using 3D microscopy. The impact fracture on the right rib III meets the description of a partial puncture. All raw measurements are shown in the Table S14. The partial puncture has an oval outline from the top view (Fig. S10). Cross-sectional analysis revealed the conical shape of the wound channel, which would reflect the shape of the projectile tip that penetrated the bone surface and pressed bone fragments into it. The maximum depth of the partial puncture is 4.2 mm, and its estimated volume of the puncture is roughly 80 mm^3. Another type of

hunting lesion consists of the potential drag marks were observed on left rib III, right rib II, right rib XI, and on the lumbar vertebra III (Fig. S11). Unfortunately, the bottoms of these potential drag marks are always coated by the consolidant used to preserve the bones, making the complete inspection of the qualities of these modifications impossible. Nevertheless, the hunting lesions on the right rib II and rib III were compared to the trampling (or bird beak/talon mark) damage on the right rib VI. The potential drag mark on the right rib XI is completely covered by the consolidant; therefore, it could not be analyzed using 3D microscopy. All measurements are summarized in Table S15. The outline of the potential drag marks forms an acute triangle with the shorter side always on the frontal ridge of the rib shafts with the point toward the caudal aspect, while the trampling has a more rectangular shape located on the curved surface of the medial aspect of the shaft. Based on the morphometric data, the left rib III revealed a width at the basis/edge of 6.9 mm while it was 5.3 mm on the right rib II. The length was 8.2 mm and 8.6 mm, respectively. Both marks display a regular V-shaped profile-line morphology with a sharp bottom basis despite the consolidant filling. Depths become noticeably shallower from the shorter side of the triangle to the point, as exemplified by the topographic 3D image in Fig. S12A, B. In the middle of the groove, the depth at the surface was 1.2 mm and 0.8 mm for left rib III and right rib II, respectively. The trampling score on rib VI consisted of two clearly discernible grooves with irregular W-shaped bottoms (Fig. S12C). The width and length are 1.1 mm and 4.6 mm, respectively, while the depth is roughly 0.1 mm. Overall, this type of modification differs considerably from the potential lesions on the other two ribs and the previously discussed cut marks. During the taphonomic analysis, a notch was observed located on the tip's right side of the spinous process, the lumbar vertebra L3. The 3D microscopy of the modification shows the V-shaped morphology of the notch supported by the morphometric analyses (Fig. S11). The width of the bone surface is 2.7 mm. The profile lines obtained a length of roughly 6.5 mm and a depth of 0.9 mm. One side of the notch shows a shallow descent compared to the other side, and the tissue seems to have been pressed in this shape. Furthermore, it should be noted that the notch is partially filled with white conservation material, which may have impacted the measurements. All measurements are shown in Table S15. Given the particular morphology that cannot be associated with the other modifications, this remains the most ambiguous bone surface modification.

3.3 Impact fracture analysis

Length and width of the partial puncture on rib NKM527/7 were compared against linear measures of length and width of pits and punctures produced by modern lions, spotted hyenas, bears, and wolves. Fig S13 illustrates the result of the comparison.

The size of the partial puncture on the Siegsdorf specimen clearly falls outside of the range of any carnivore tooth marks. This result is similar to the lesions from the later site of Neumark Nord 1, where hunting lesions identified on cervids also fall outside of the sizes of carnivore tooth marks[44]. The morphological features of the Siegsdorf specimen further support this observation, as the partial perforation exhibit multiple circumferential cracks and bone fragments (compacta) pressed into the wound channel, which are not typical signatures of bite marks[44]. Furthermore, other signs of carnivore modification on the same element or the rest of the skeleton would be expected if the agent that caused the damage was a non-human animal[28]. Taken together, these observations provide compelling evidence that the partial puncture on the Siegsdorf cave lion was most likely caused by a human rather than a carnivore.

When compared with experimentally produced or identified punctures in the archaeological record, the size of the partial puncture on the Siegsdorf lion lies in the middle of the data point cluster, fitting well within the range of all the groups (Fig S14).

The classification analysis supports this result, as shown in Fig S15. The damage on the Siegsdorf rib fits well within the Wooden Tip and Archaeological Hunting Lesion groups, and the analysis classifies this mark as belonging to the Wooden Tip category (Table S17). The Jackknifed classification has a success rate of 45.16%, and therefore this result should be interpreted with caution. Limitations of this result are likely due in part to a dearth of experimental reference material, and the fact that some of the hunting lesions grouped as archaeological might include those caused by wood-tipped weapons.

Unlike projectile weapons, such as arrows or javelins, which rapidly lose energy upon impact, a thrusting spear maintains its force as the hunter continues to exert pressure after making contact. The spear's penetrating force persists until the hunter encounters a surface through which the spear cannot penetrate or determines that the depth of penetration is sufficient[80]. This characteristic, along with the classification analysis and the ballistic analysis, supports the interpretation that the lesion was caused by a thrust from a wooden spear.

3.4 U-series and combined US-ESR dating results on a fossil tooth from Einhornhöhle

ESR dose evaluation. Given the limited amount of enamel extracted from tooth #597, ESR measurements were carried out with aliquots of ca. 12 mg (Table S5). Despite the small size of these aliquots, ESR measurement precision achieved is overall excellent, with a variation of only 0.6% of the ESR intensities over repeated measurements (Table S5). This results in a high D_E repeatability of 3.4%. D_E value obtained over the full irradiation dose range using SSE fitting function (and data weighting by $1/I^2$) is of ca. 253 Gy (D_{E1}; Table S5). The corresponding D_{max}/D_E ratio value (5.9) falls within the recommended range ($5 < D_{max}/D_E < 10$) by Duval and Grün [81] for D_E values of this magnitude (< 500 Gy). Additional dose response curve fitting was performed using a SSE function with data weighting by $1/s^2$, resulting in a slightly lower D_E value by <2 % (D_{E2} = 250 Gy; Table S5). These results illustrate the very limited impact of data weighting on the fitting outcome. Overall, there is a series of proxies (high measurement and D_E repeatability; no significant fitting bias) showing the robustness of the ESR data collected for sample #597.

Solution U-series analyses. Solution U-series analyses of the bulk powdered dental tissues from #597 show that (i) the enamel tissue have relatively low uranium concentration (<1 ppm), and (ii) there is no evidence of uranium leaching in any of the samples (i.e., finite age can be obtained for all samples). These two observations indicate that the samples are suitable for ESR dating.

Apparent U-series age estimates of ca. 150 and 128 ka were obtained for the dentine and enamel, respectively (Table S6). They should be regarded as minimum age constraints for the fossil, since dental tissues behave as open systems for U-series elements. No significant detrital Th contamination is overall observed in the samples, resulting thus in very limited corrections of the apparent ages (<0.5 ka). The younger apparent U-series age result obtained for the enamel most likely results from the slowest diffusion rate of uranium into that tissue due to its lower porosity and higher crystallinity compared with dentine (e.g.,[82]). To sum up, in first instance the apparent U-series age results indicate that the age of the tooth is > 150 ka.

Combined U-series and ESR age calculations. Combined U-series and ESR age calculations carried out with the US model return an estimate of 189 +16 -14 ka for sample #597 (Table 7). The total rate is dominated by the dental tissues, with the enamel and dentine contributing by 20% and 42%, respectively. Calculated *p*-values are close to -1, indicating a relatively early uranium uptake in both tissues. We identify two main sources of uncertainty that may potentially impact the calculated ages. These are related to (i) the uranium uptake modelling in dental tissues and (ii) the evaluation of the gamma dose rate.

First, assuming that dental tissues behaved as closed system after incorporating the uranium (CSUS model) leads to the calculation of a CSUS-ESR age of 234 ± 22 ka. This age is about 24%

older than the US-ESR estimate initially calculated and should be regarded as a maximum possible age for #597 based on the assumption of an extreme uranium uptake scenario.

The absence of in situ dosimetry may result in a hardly quantifiable uncertainty on the gamma dose rate. However, sediment heterogeneity may reasonably be considered as limited in first instance given the small variability of radioelement concentration values (7-15%, Table S8) measured the two sediment samples collected from the surrounding of the tooth. Deriving beta and gamma dose rates from samples #597sedA and #597sedB returned age estimates of ca. 180 and 198 ka, respectively. These results remain within 1σ error (± 9 ka) compared with the US-ESR age initially calculated. Additionally, field observations indicate that the distance of the cave wall from the tooth was >30 cm, suggesting thus that its influence on the gamma dose rate is most likely null, or at least negligible. Finally, the impact of the long-term WC was assessed by considering two extreme values of 10% and 30%, which resulted in combined US-ESR age estimates of ca. 177 and 203 ka, respectively. Again, the results remain within 1σ error with the US-ESR age initially calculated, indicating that the variability of long-term WC has limited influence on the calculated age.

To sum up, combined U-series/ESR calculations return an age result of 189 +16 -14 ka for sample #597. While we do acknowledge the existing uncertainty associated to some aspects of the dose rate evaluation (e.g., U-uptake modelling, gamma dose rate evaluation), our sensitivity tests return age estimates within the range of 177-234 ka, providing the sample either an early MIS6 or a MIS7 age[16].

4. Figures SI

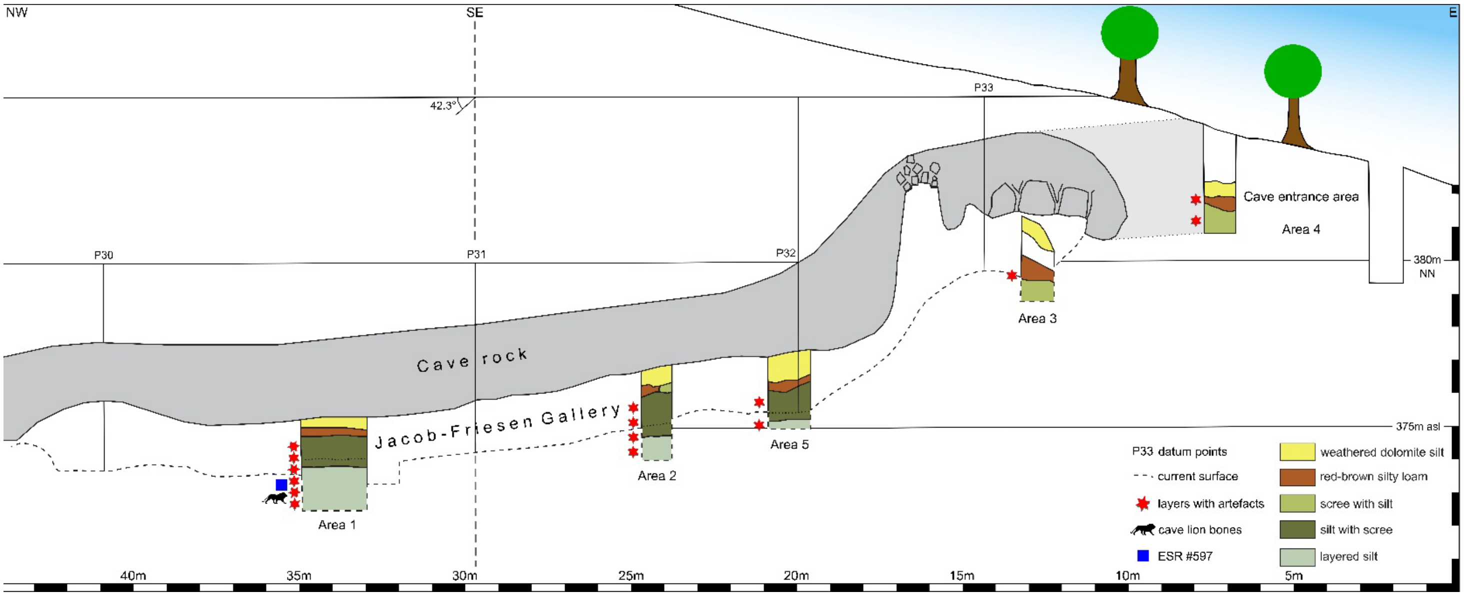

Fig. S1. Einhornhöhle. Profile of the Jacob-Friesen Gallery with the different excavation areas.

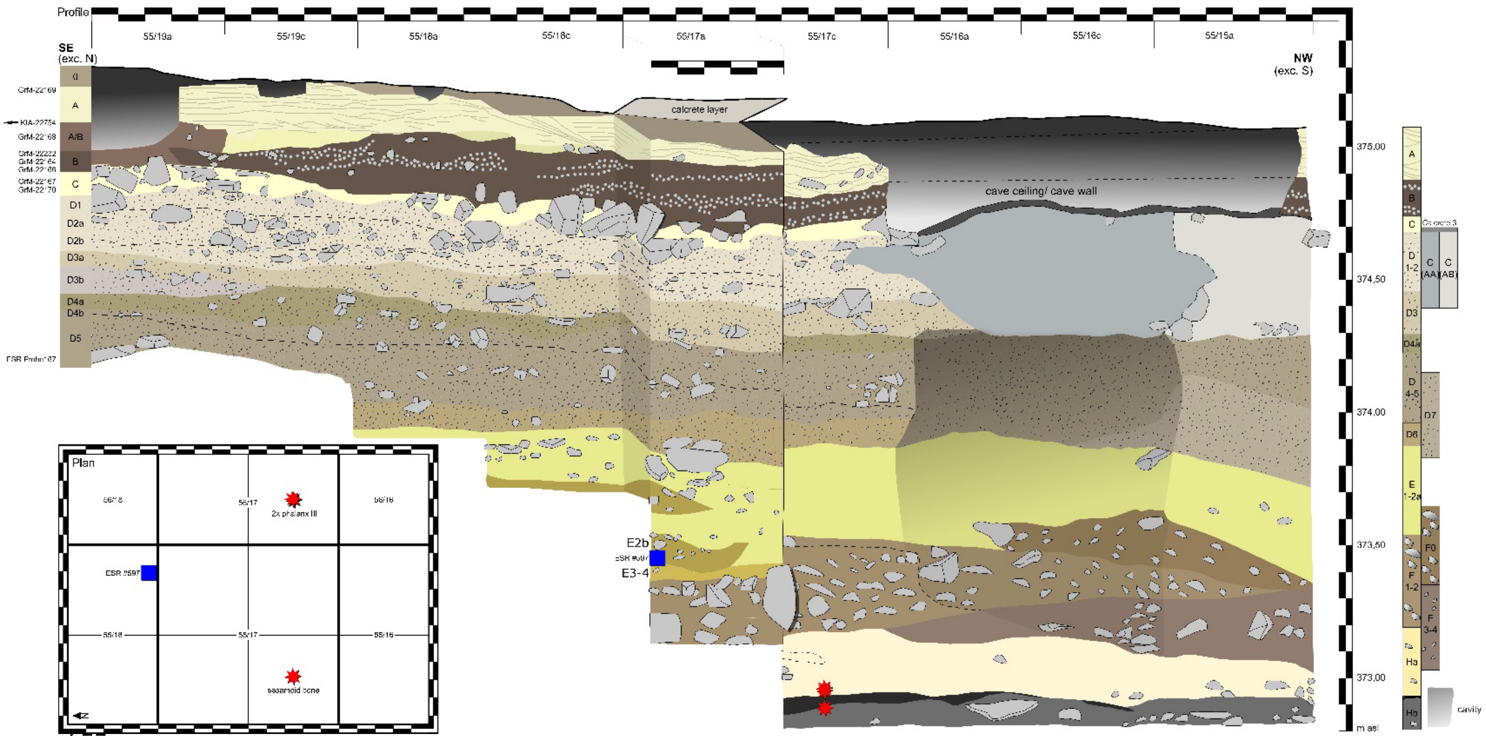

Fig. S2. Einhornhöhle. Area 1, Profile 3 stratigraphic sequence. Bottom-left the excavation grid on the horizontal plane. Red stars = lion bones; Blue square = ESR sample.

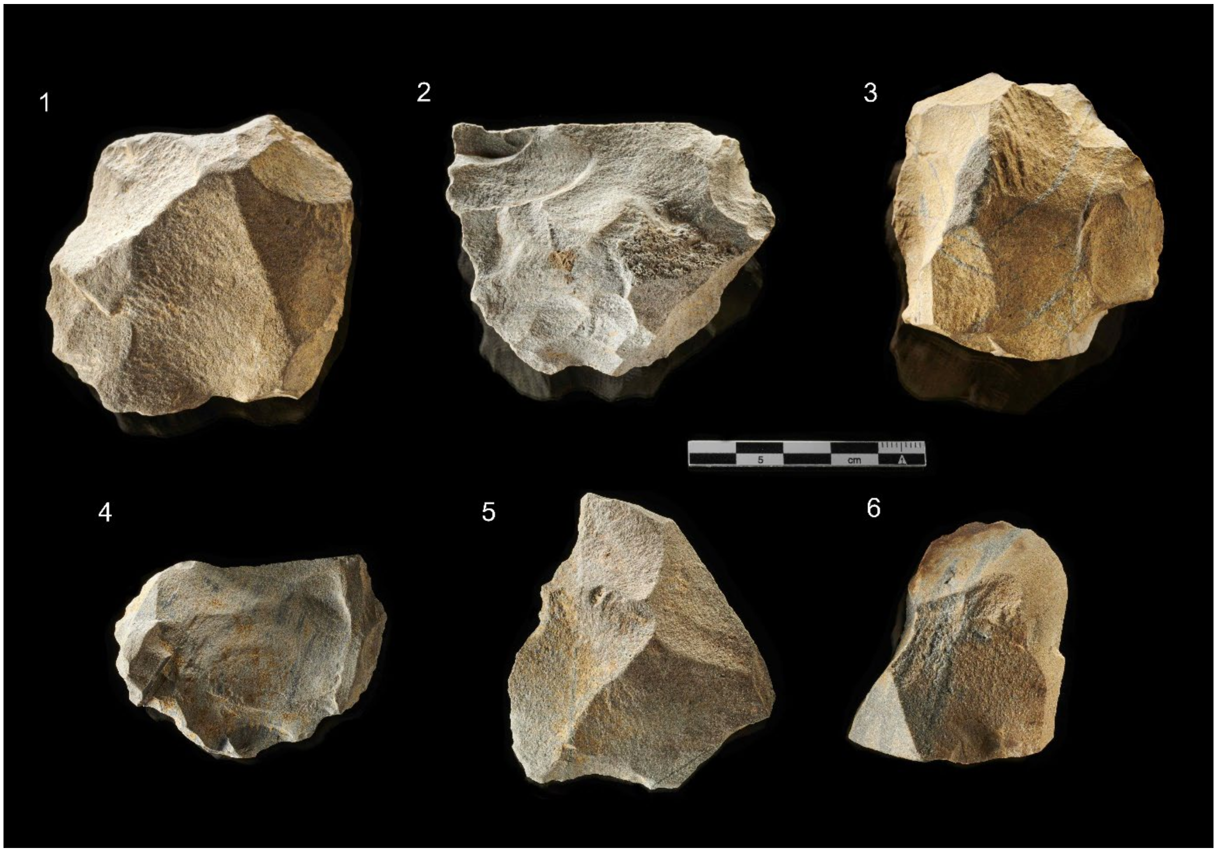

Fig. S3. Einhornhöhle. Lithic artifacts from the JFG: **1**, **3** Levallois core, **2** Proximal fragment of a biface, **4** Levallois flake, **5** notched Levallois flake with dorso-ventral retouch, **6** Flake with distal use-wear, Photo: V. Minkus, © NLD.

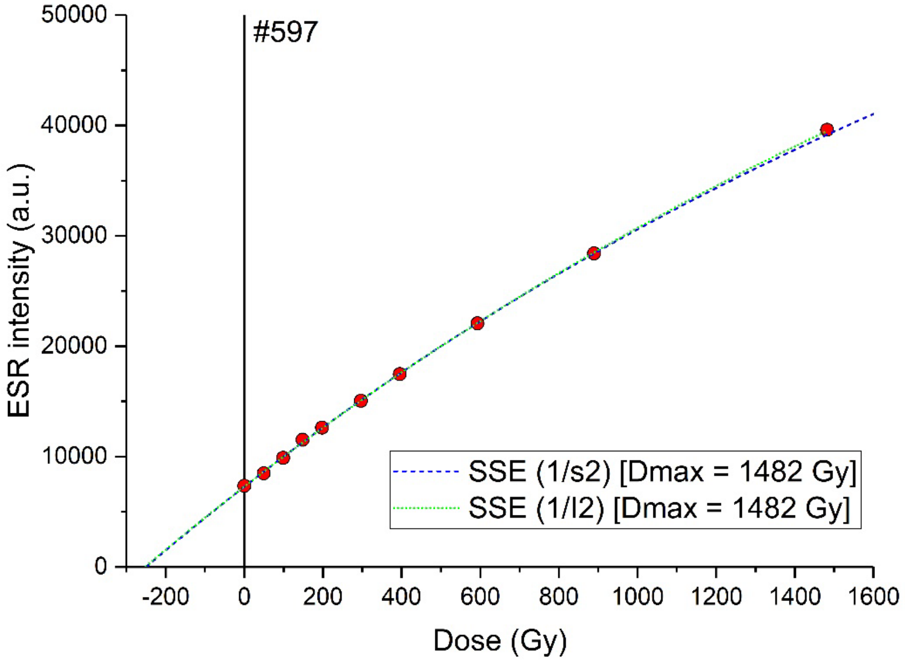

Fig S4. ESR dose response curve obtained for tooth enamel sample #597. The ESR intensity of each aliquot correspond to the mean value derived from the repeated ESR measurements. Fitting was performed using an SSE with data weighting by $1/I^2$ (D_{E1}, Table S5) and $1/s^2$ (D_{E2}, Table S5).

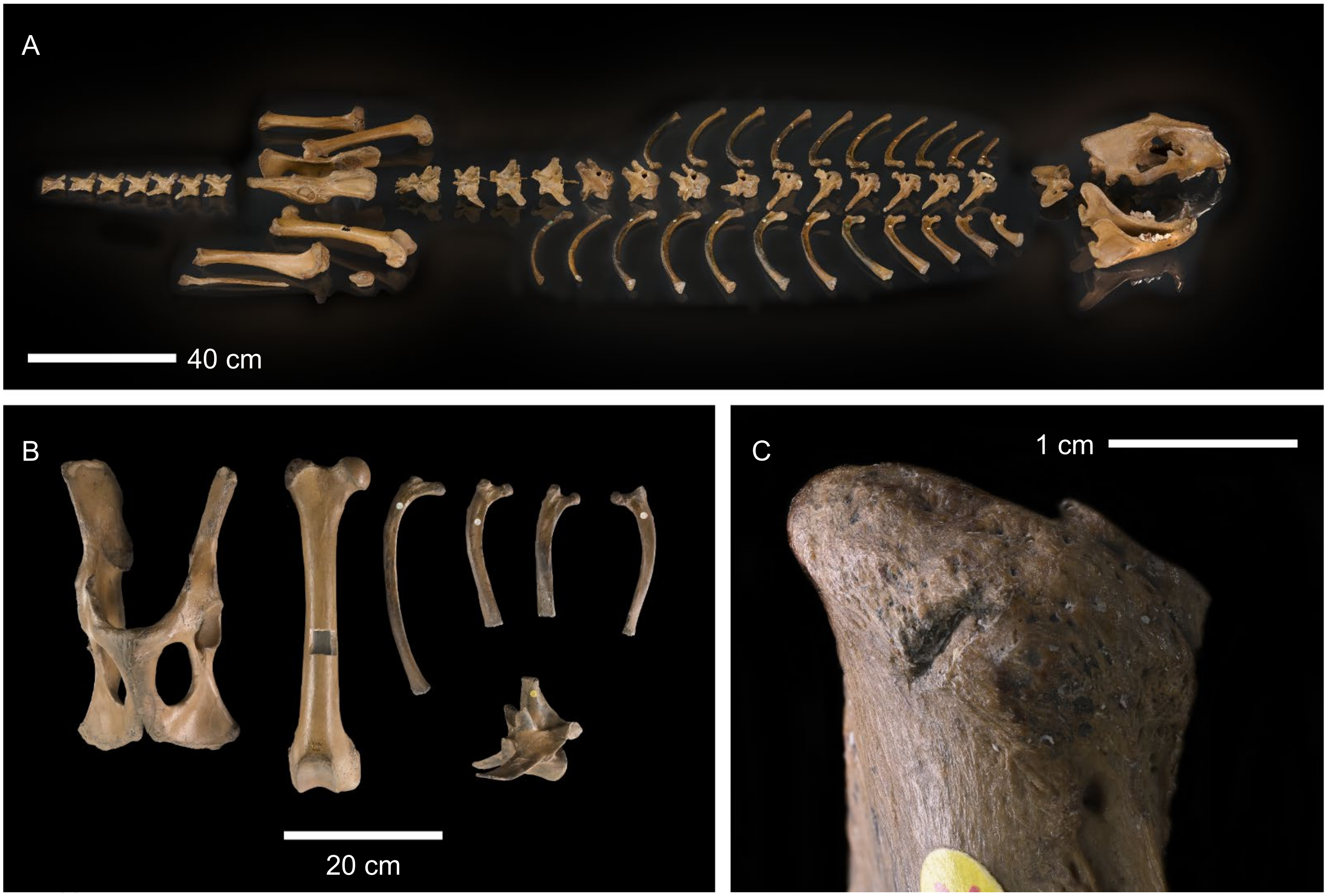

Fig S4. A. All skeletal elements of the Siegsdorf lion analyzed in this study in anatomical position; **B**. skeletal elements of Siegsdorf lion with cut marks (innominate, femur and right rib VI) and hunting injuries (right rib **II**, right rib **III**, left rib **III**, right rib VI, lumbar vertebra **III**); **C**. Close-up of the potential drag mark on the spinal process of the lumbar vertebra **III**. Photo: V. Minkus, © NLD.

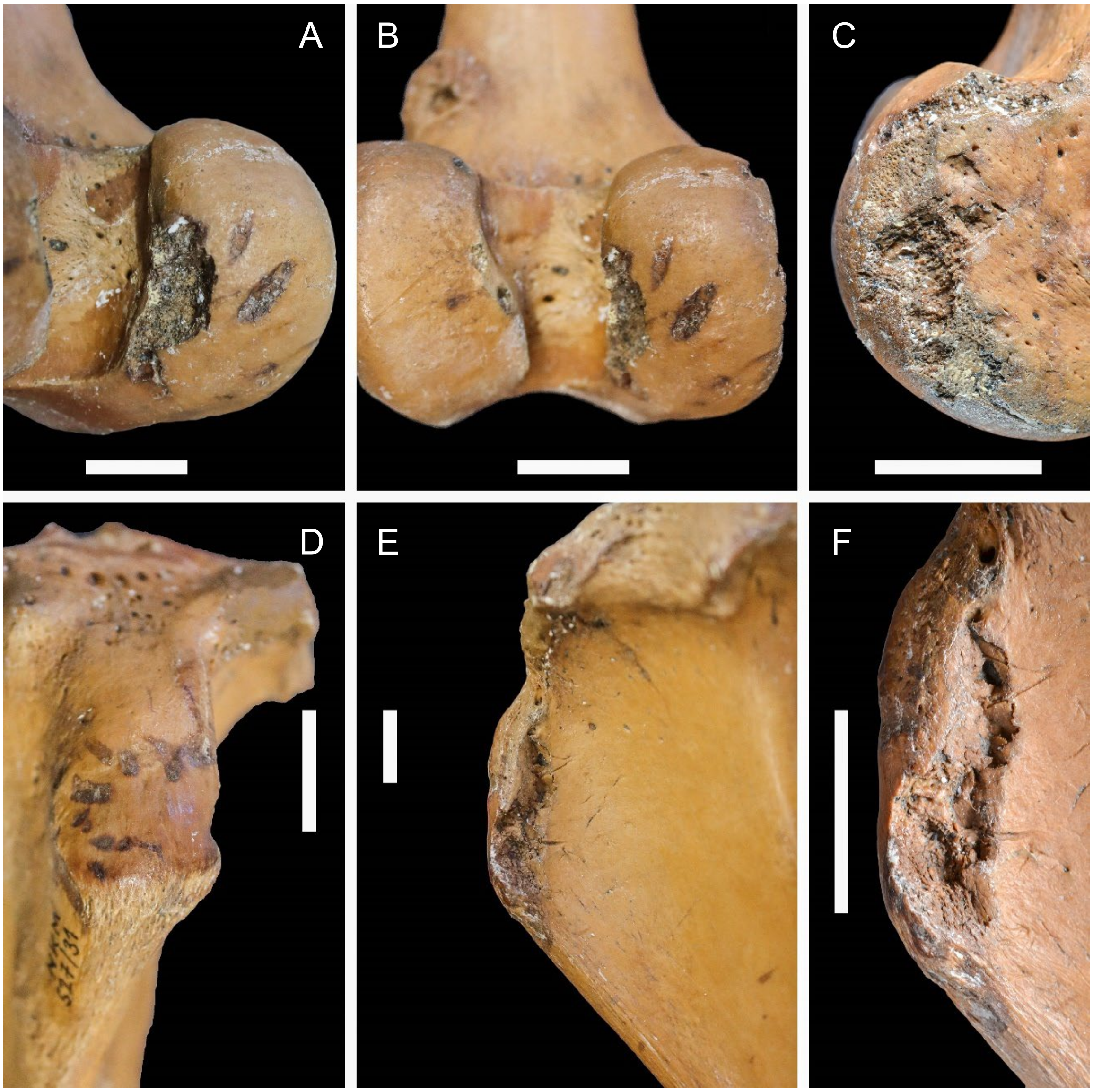

Fig. S5. Cave Lion from Siegsdorf: **A, B:** Different views of the distal epiphysis of the left femur. Note the different types of carnivore damage such as extreme crushing, broad and shallow grooves, and tooth pits; **C.** Close-up of the medial condyle of the left femur displaying abrasion and carnivore punctures; **D.** Close-up of the proximal epiphysis of the left tibia. Note the shallow and darker pits with different size and orientation on the tibial tuberosity; **E, F:** Different views of the proximal epiphysis of the left tibia. Note the micro-crushing and abrasion damage on the lateral side of the tibial tuberosity. Scale 2 cm.

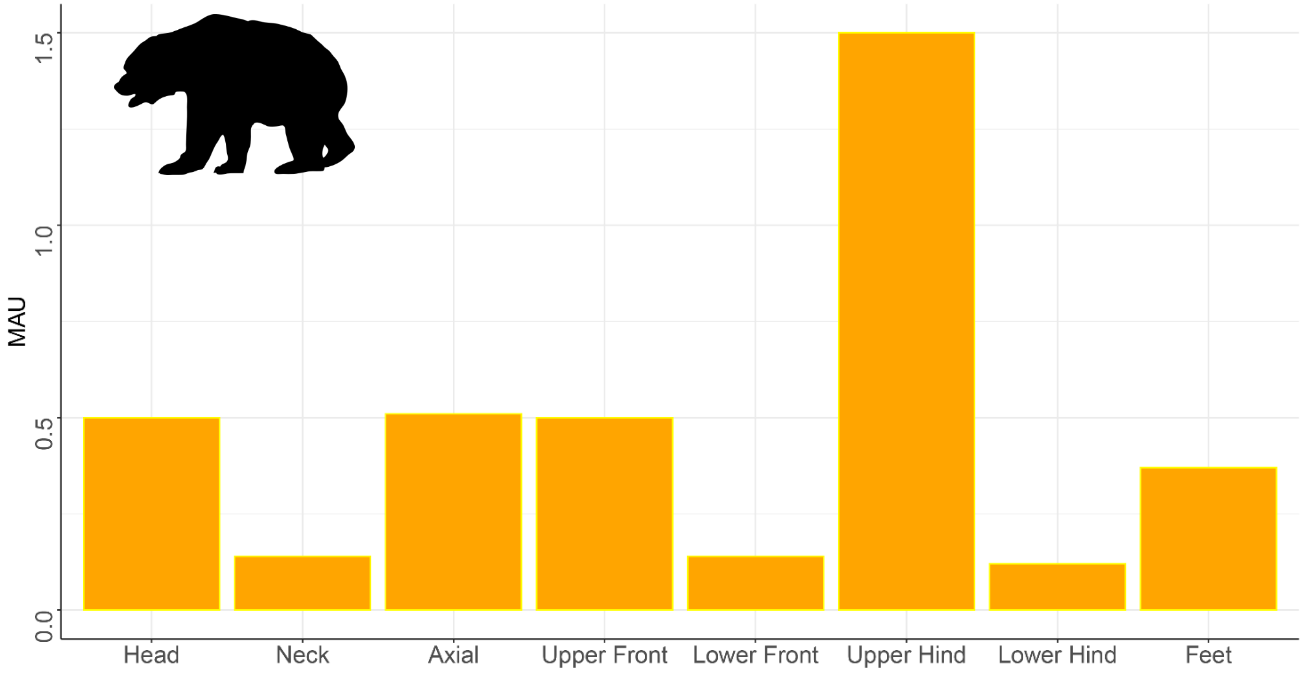

Fig. S6. Einhornhöhle. Skeletal element representation of bear remains in layer H.

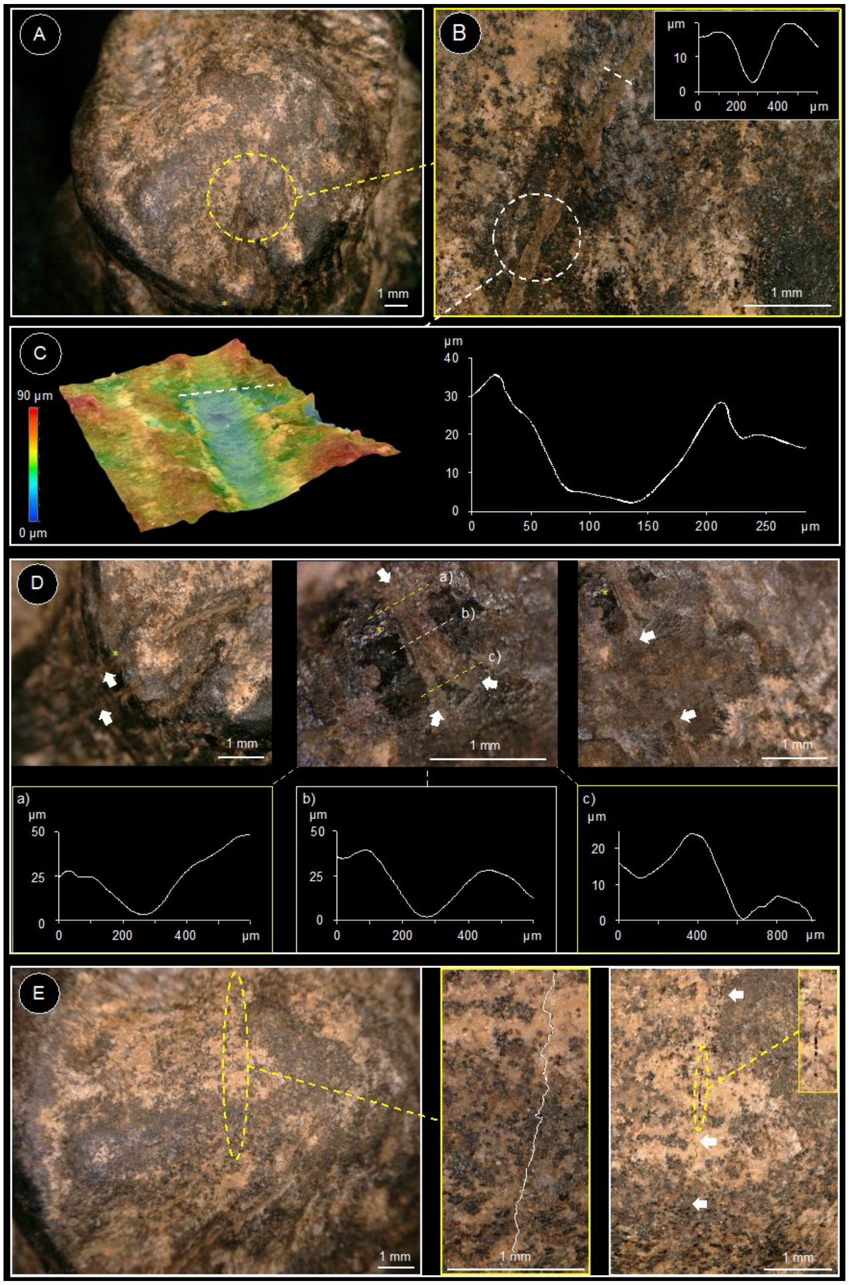

Fig. S7. Einhornhöhle. Digital 3D reflected light microscopy images of the cut mark. The yellow star is for orientation within the images. Scale bars: 1 mm. **A**. Overview image of the cut mark on the bone. Magnification of 20×; **B**. Composite image of the center of cut mark captured with a magnification of 100×. The dashed line indicated the topographic profile measurement; **C**. Composite image captured with a magnification of 1000×. Topographic profile at the position as indicated in B and profile line measurement at the dashed line in the profile left; **D**. Cut mark traces at the edge of the bone at different views of perspective. The dashed line in the middle image represents profile line measurements shown in the three graphs a)-b). Magnification of 150× (left and middle) and 50× (right); **E**. Composite images of a surface crack imaged at magnifications 30× (left), 100× (middle), and 200× (right). In the middle image, the crack was highlighted in white.

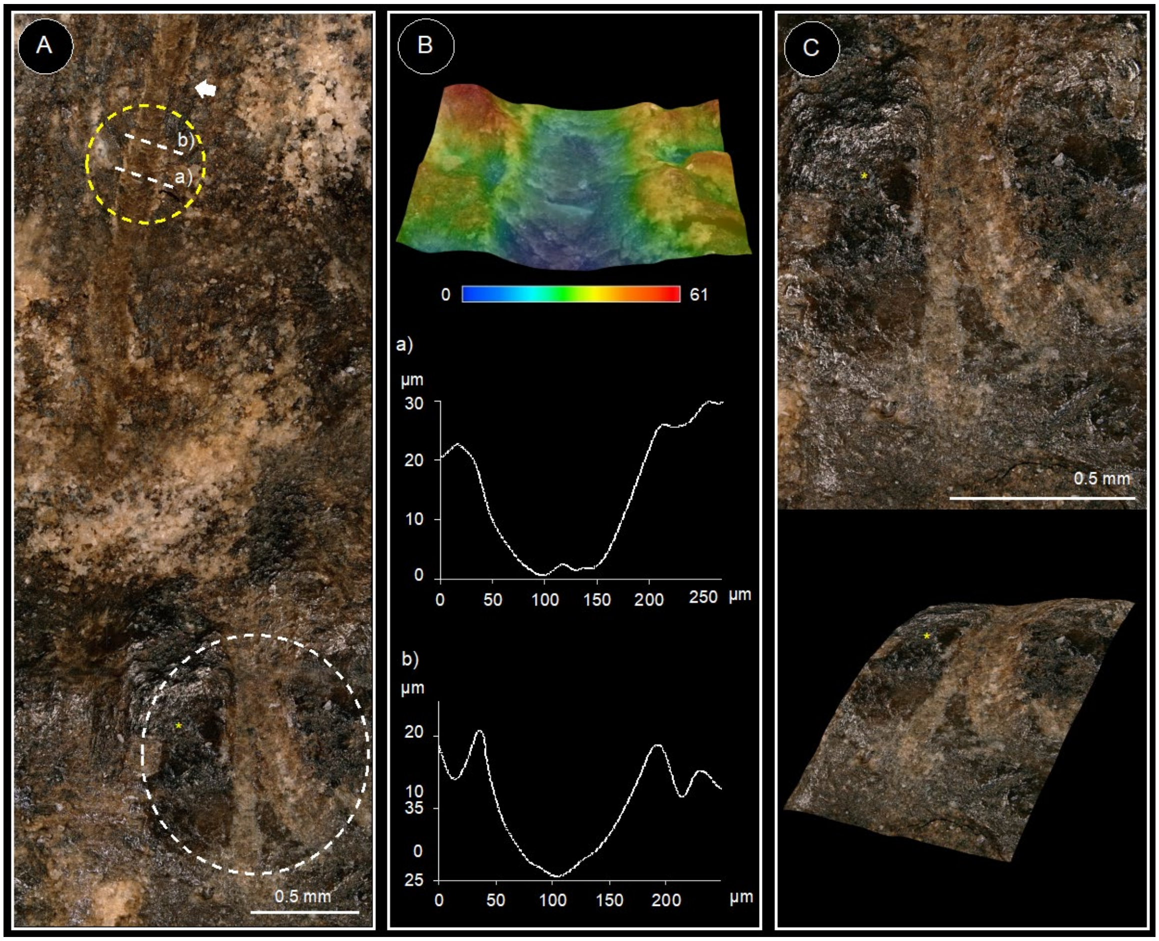

Fig. S8. Einhornhöhle. Digital 3D reflected light microscopy images of the cut mark and the incision at the edge. The yellow star is for orientation within the images. Scale bars: 0.5 mm. **A**. Composite overview image of the incisions on the bone. It should be noted that the entire cut mark, indicated by an arrow, is not depicted. Magnification of 200×; **B**. 3D image of the cut mark captured with a magnification of 1000×. The position is highlighted with a dashed yellow circle in A. The dashed white line indicated the topographic profile measurements (a, b); **C**. Composite image of the incision at the edge captured with a magnification of 200×. The position is highlighted with a dashed white circle in A.

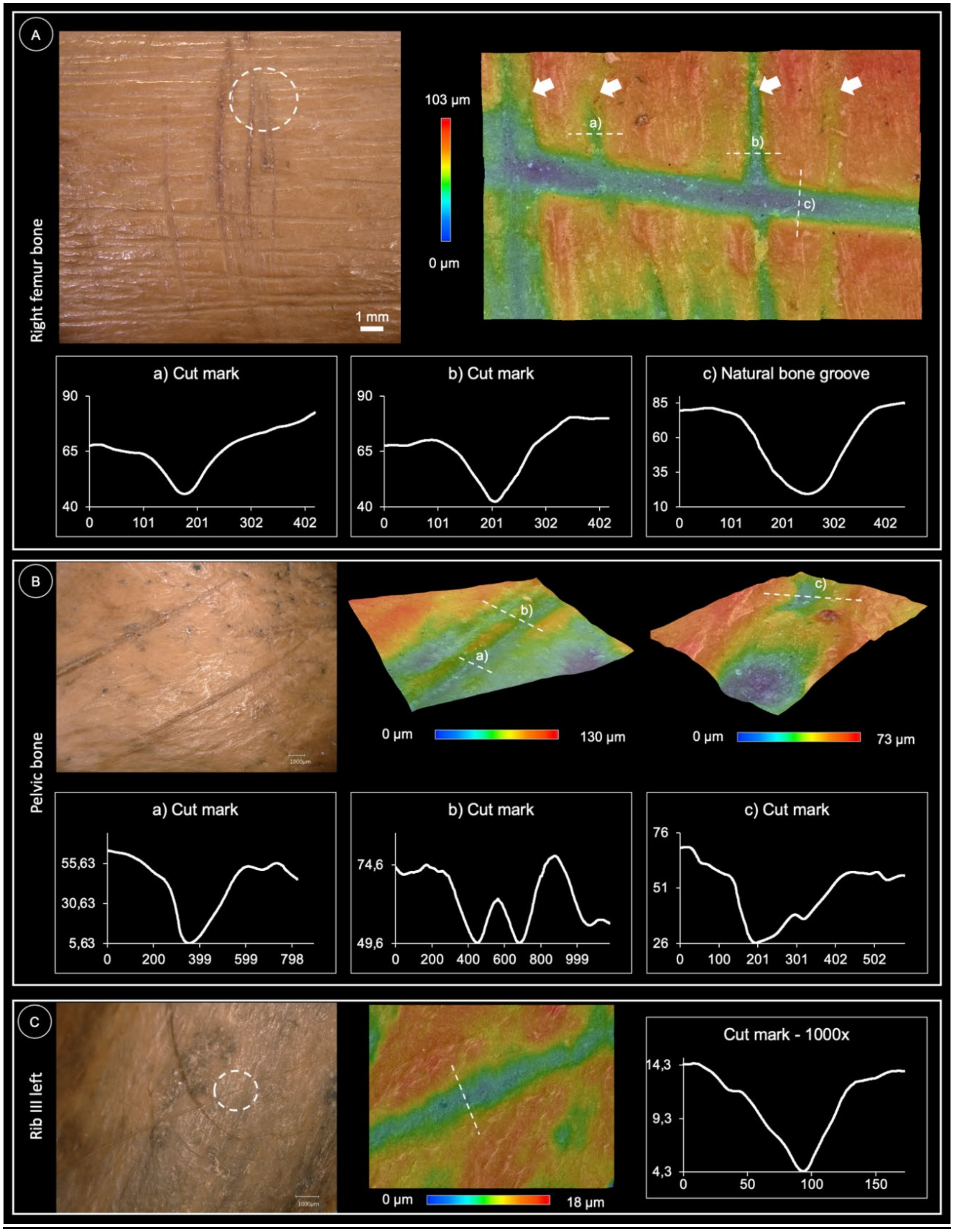

Fig. S9. Cave lion from Siegsdorf, cut marks. **A**. Microscopic examination of the cut mark on the right femur bone at a magnification 500×; **B**. Cut marks on the pelvic bone. Images captured at magnifications of 150× to 500×; **C**. Cut marks on the left rib III. Image were captured with 30× and 1000× magnification.

.

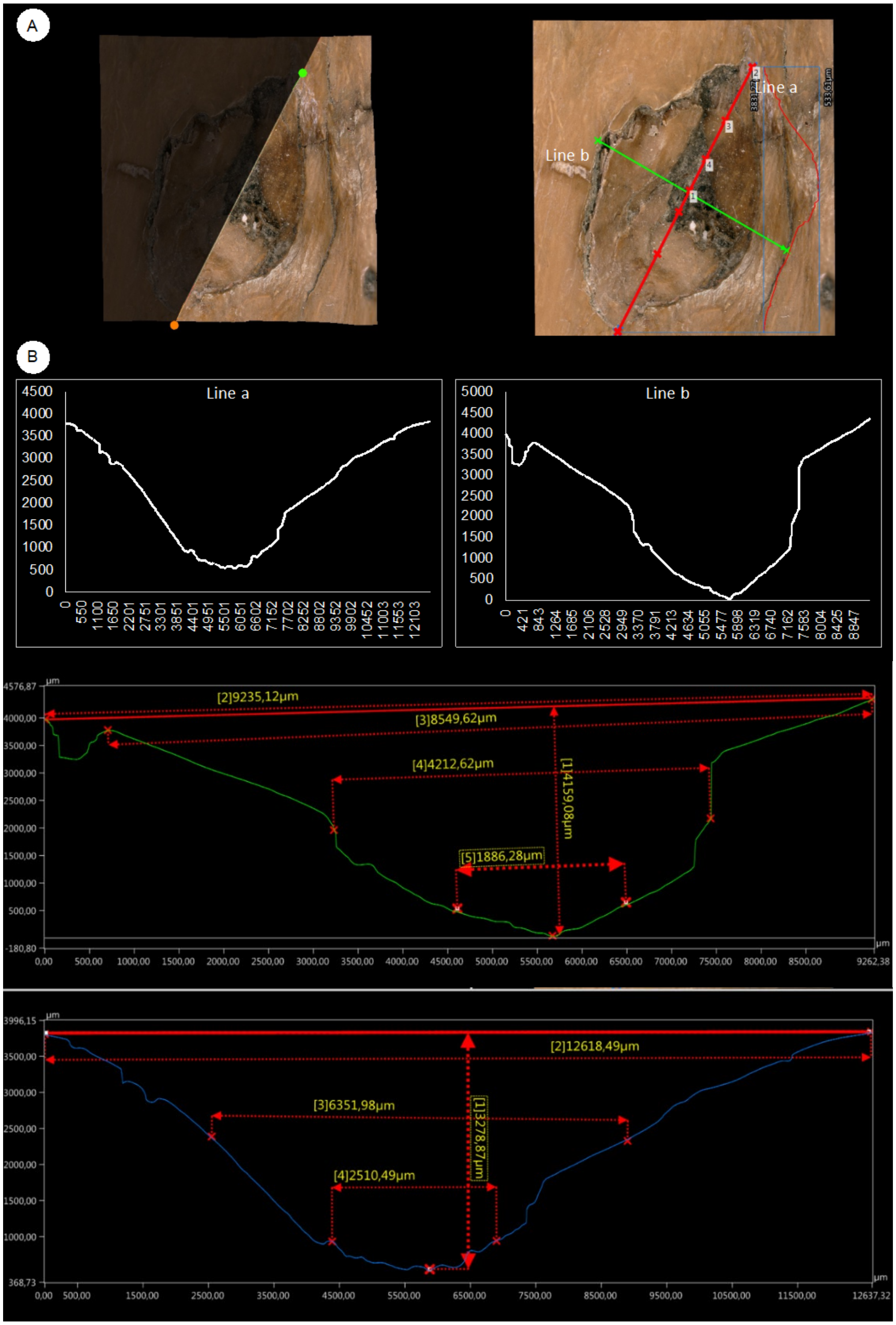

Fig. S10. Cave lion from Siegsdorf, profiles of length (line a) and breadth (line b) of the impact fracture. **A**. VHX imaging highlights the position of the analyzed profile lines (a+b); **B**. Profile lines for line a and line b.

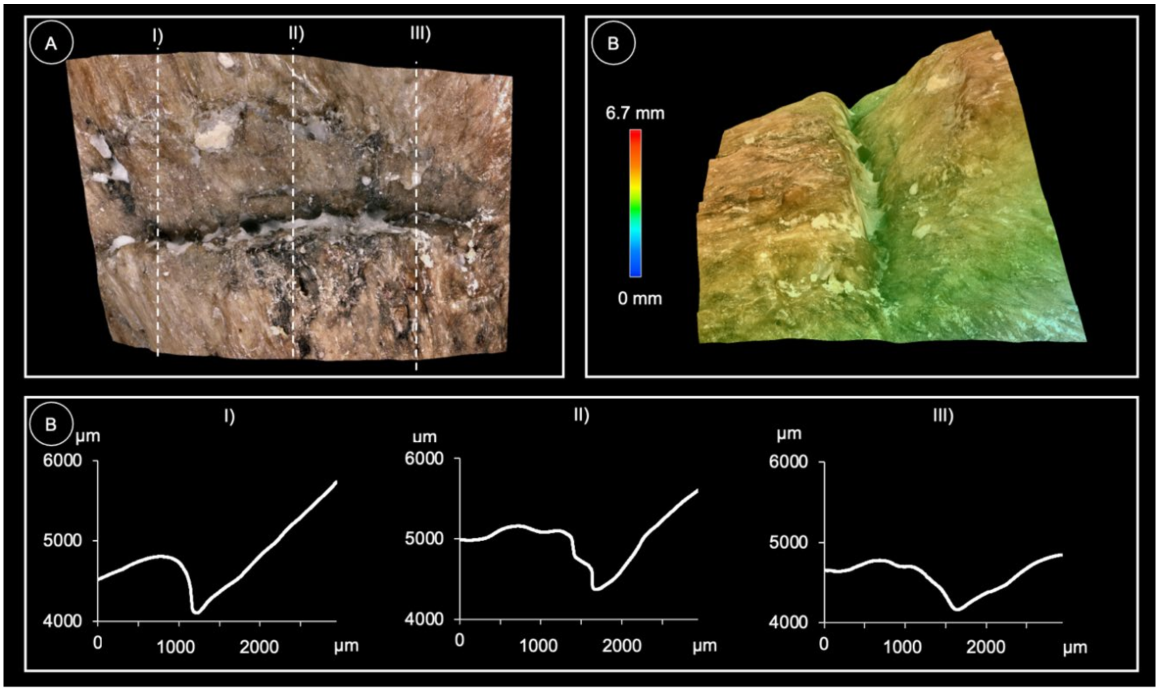

Fig. S11. Cave lion from Siegsdorf, notch on the lumbar vertebra III at magnification of 100×. **A**. 3D composite image of the notch; **B**. Topographic 3D image; **C**. Profile-line measurements captured perpendicular to the notch at three positions.

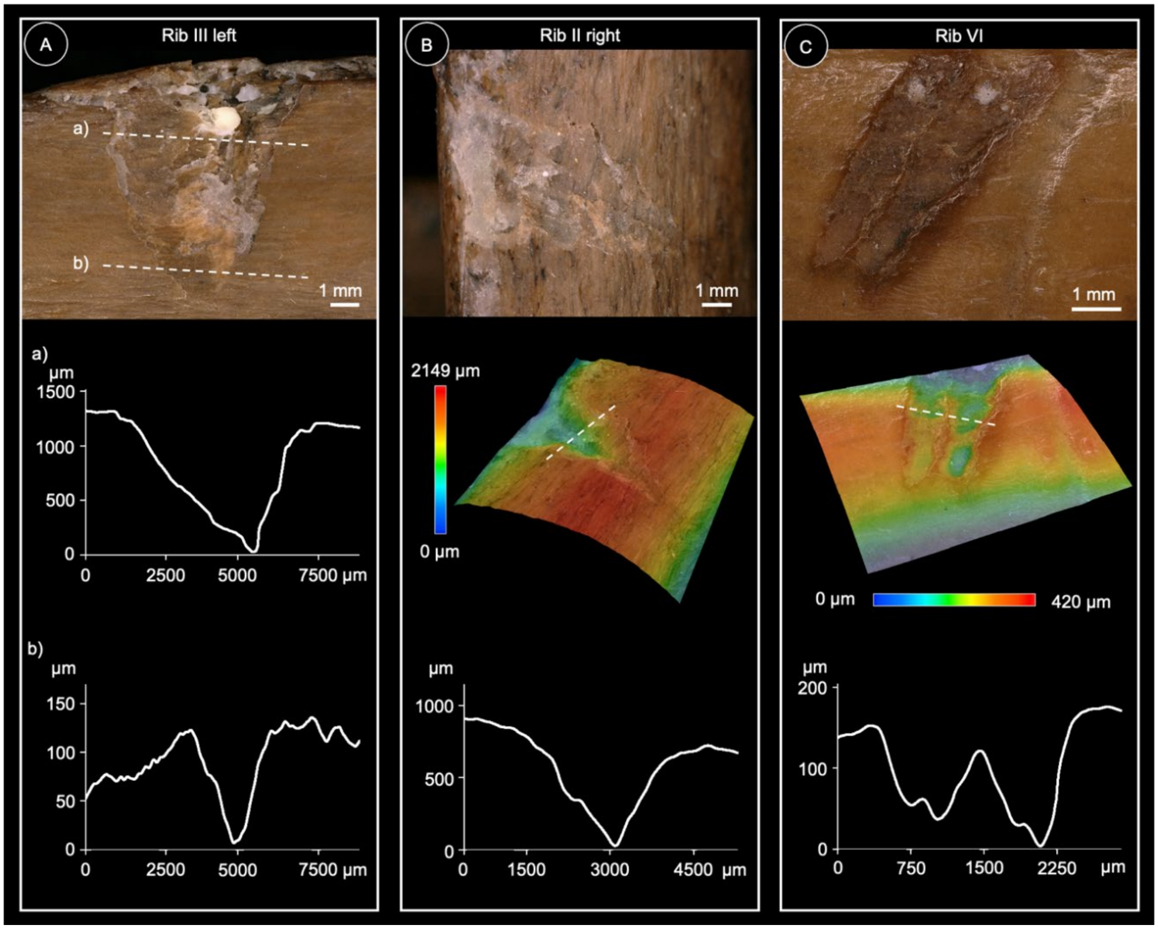

Fig. S12. Cave lion from Siegsdorf, potential drag marks found on ribs. **A**. Mark on left rib III. Overview composite image at a magnification of 50×. The dashed lines (a, b) represent the displayed profile lines below; **B**. Mark on right rib II. Overview composite image captured with a magnification of 100× with a topographic 3D image at a magnification of 100×. The dashed line in the 3D image shows the position of the profile line below; **C**. Marks on the rib VI. Overview composite image and corresponding 3D image captured with a magnification of 100×. The dashed line in the 3D image shows the position of the profile line below

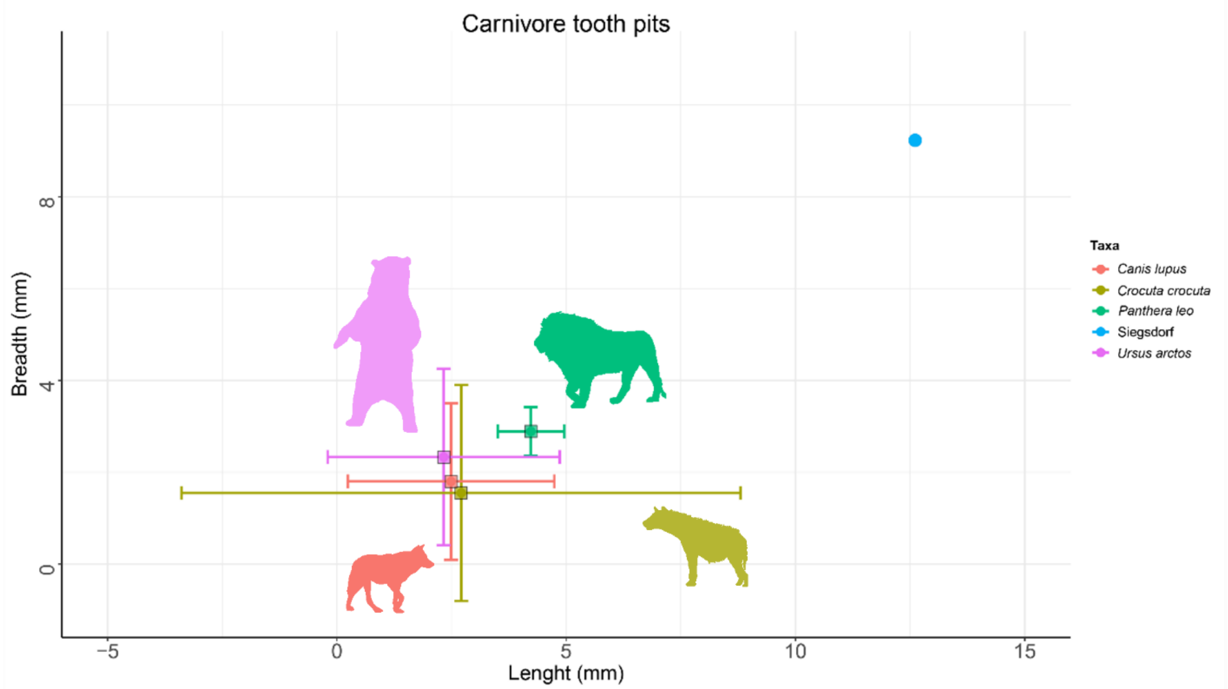

Fig S13. Maximum length and breadth of carnivore tooth pits corrected with their standard deviation against the partial puncture on the cave lion from Siegsdorf.

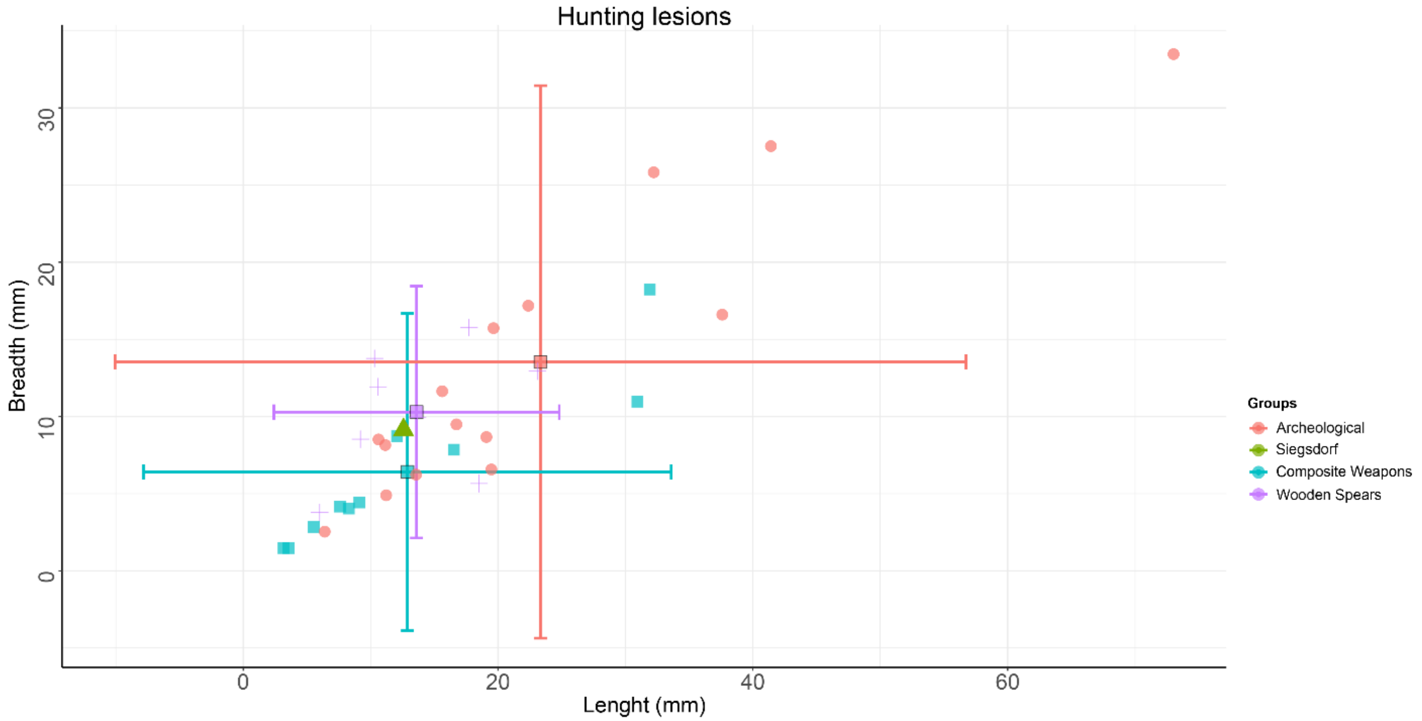

Fig. S14. Metric comparison of hunting lesions from the literature against the partial puncture on the cave lion from Siegsdorf using maximum length and width with standard deviation.

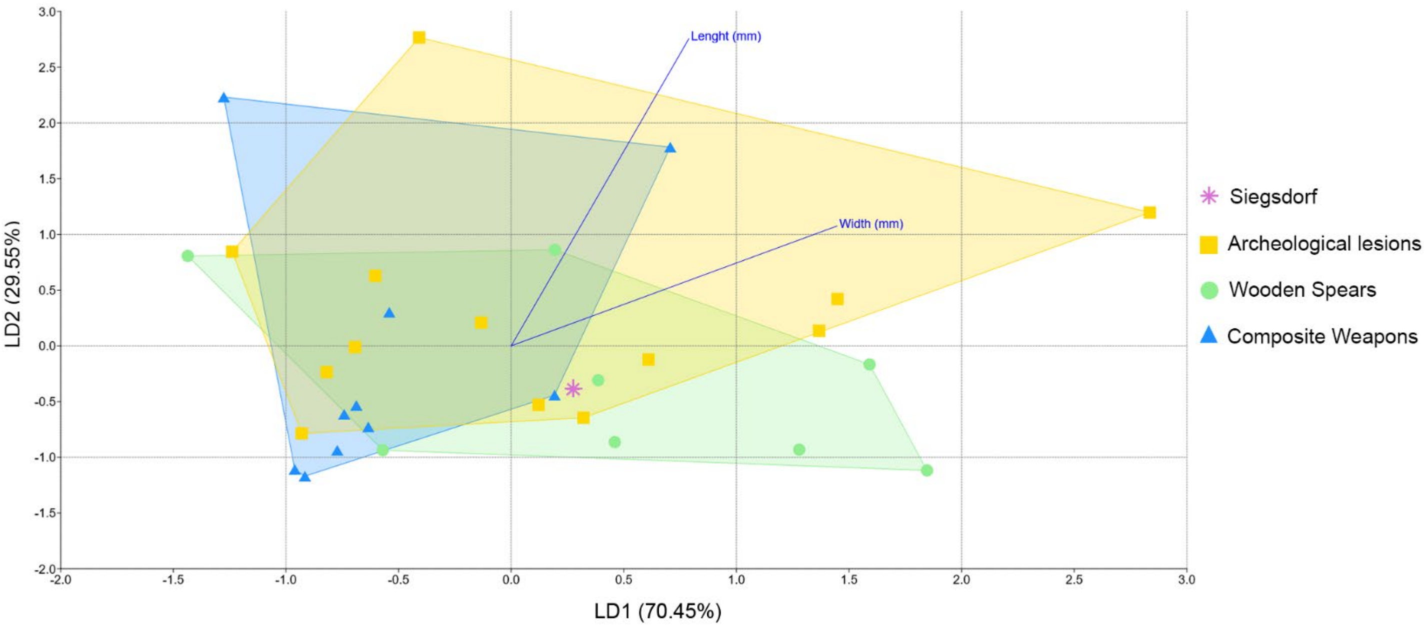

Fig. S15. Plot of linear discriminant analysis. Partial puncture on the Siegsdorf cave lion was treated as unknown against the dataset groups.

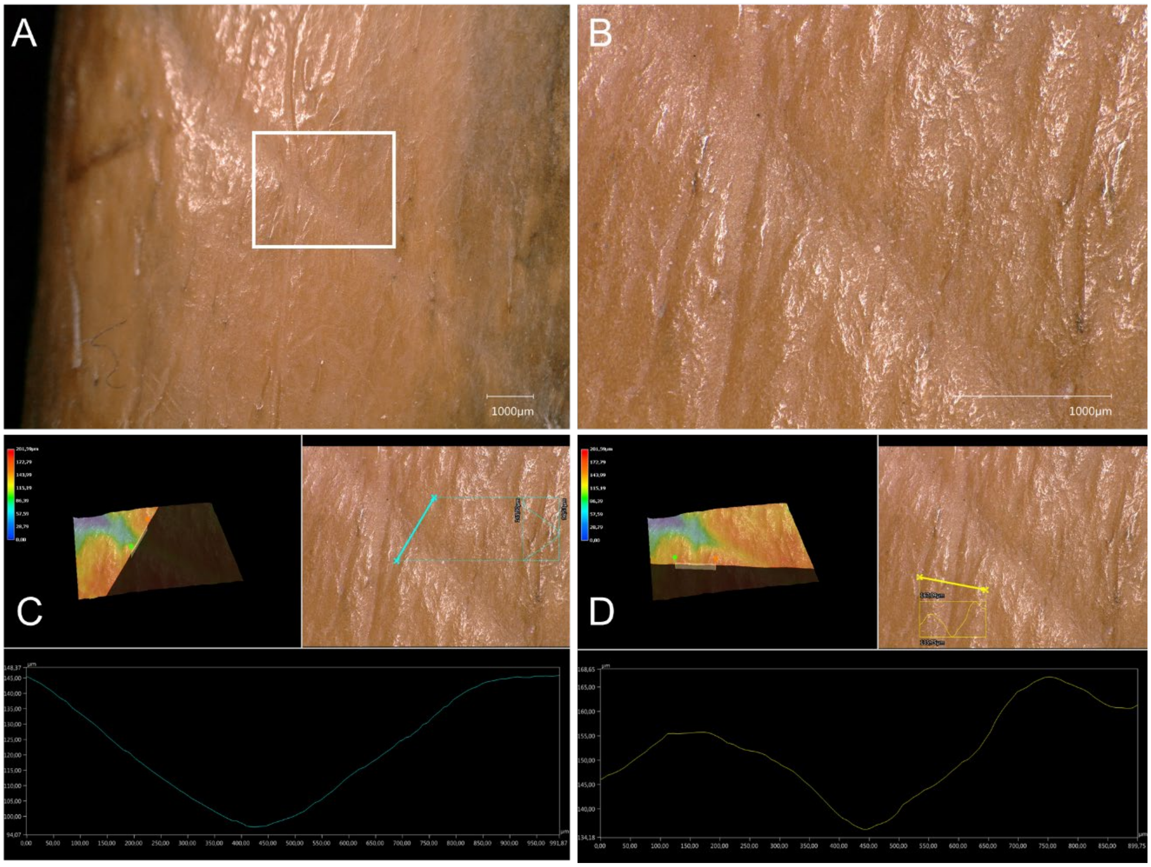

Fig. S16. Cave lion from Siegsdorf, Examples of linear marks due to trampling on the left rib III: Note the two shallow V-shape grooves of different size and orientation that overlap.

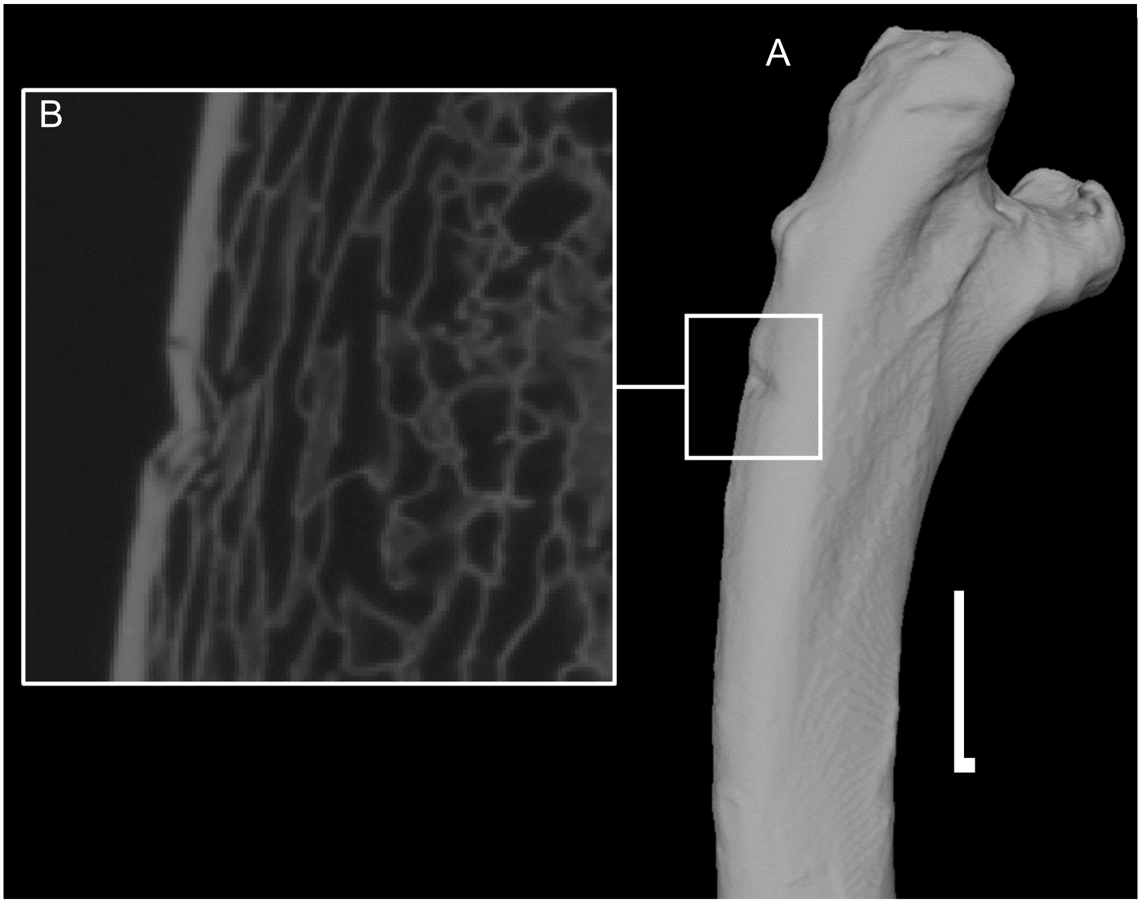

Fig. S17. Cave lion from Siegsdorf. **A.** 3D model of the right rib II exhibiting a potential drag mark on the later aspect of the *collum costae*. Scale 2 cm. **B.** CT Longitudinal slide of the *collum costae*. Note the trabecular tissue within the lesion is compressed inwards suggesting that the tissue was still fresh when the damage occurred.

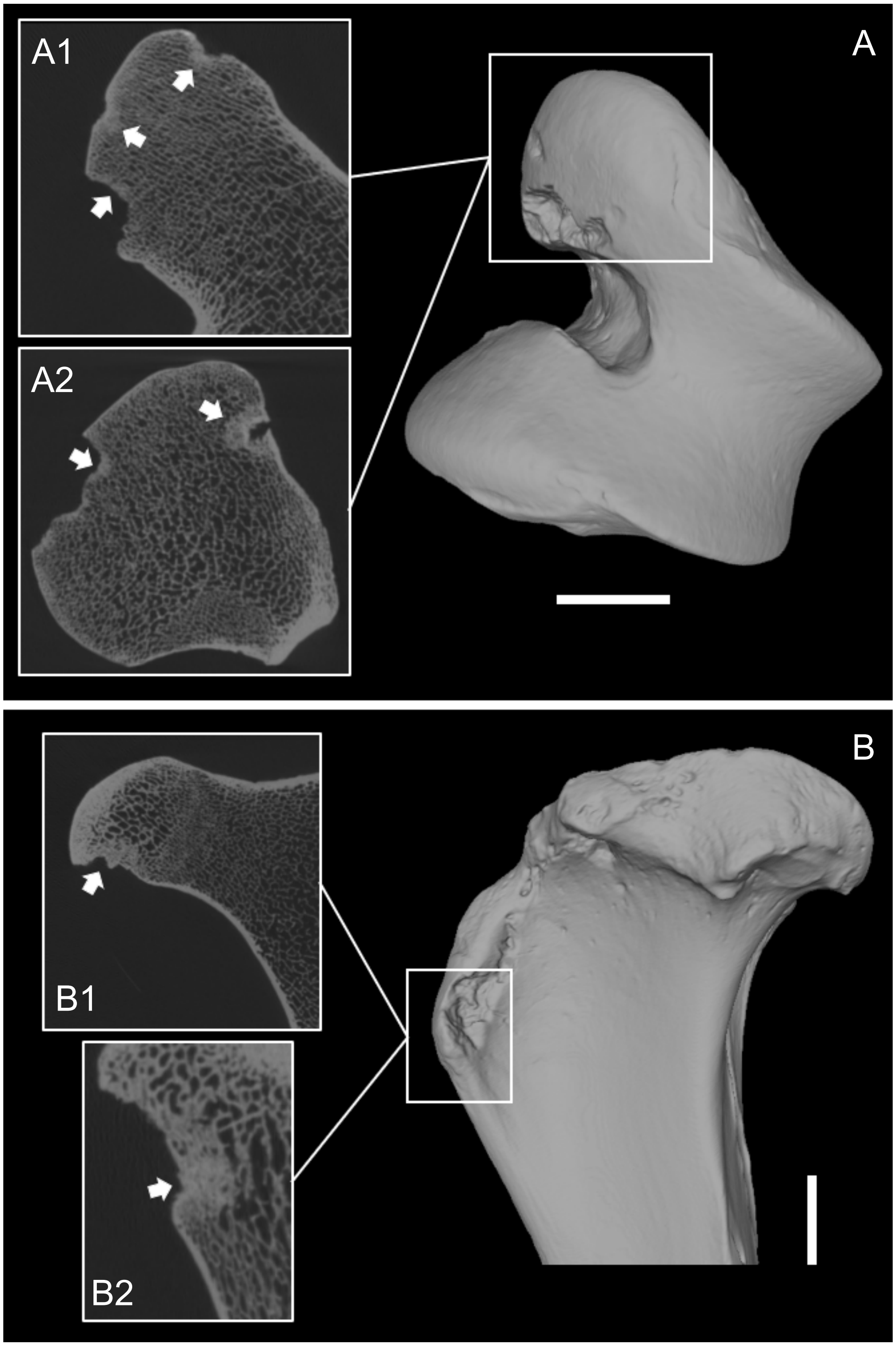

Fig. S18. Cave lion from Siegsdorf. **A.** 3D model of the left femur distal epiphysis. Scale 2 cm. **A1**; CT cross-section of the medial condyle of the left femur. Note the absence of fracture of the trabecular tissue around the lesion, and the radio-opacity around the damaged areas; **A2** CT tangential-section of the medial condyle of the left femur. The radio-opacity around the lesions is well visible also in this view; **B.** 3D model of the left tibia proximal epiphysis. Scale 2 cm; **B1.** CT cross-section of the tibial tuberosity showing radio-opacity around the damage; **B2.** Longitudinal section of the tibial tuberosity showing the radio-opacity and the absence of trabecula tissue crushing around the abraded area.

5. Tables SI

Table S1. JFG: artifact classes by layer.

Blanks/cores	D	E	F	G	H	I	other	Area 4	Total	%
chip	27	111	61	20	127	22	11	2	381	51.6
chunk	4	45	41	14	122	16	12	3	257	34.8
flake	7	22	11	6	19	3	4	1	73	9.9
blade/let	2	2	1	1	2	0	2	2	12	1.6
core	0	5	2	1	1	0	0	0	9	1.2
other	1	0	2	1	2	0	1	0	7	0.9
Total	41	185	118	43	273	41	30	8	739	100.0
%	5.5	25.0	16.0	5.8	36.9	5.5	4.1	1.1	100.0	

Table S2. Einhornhöhle. Summary of the radiometric dates of the JFG.

^{14}C															
**LabID**	**FindID**	**layer**	**material**	**δ13C AMS [‰]**	**C [%]**	**Collagen [%]**	**C:N**	**pMC**	**^{14}C-age**	**standard deviation**	**calBP 2-sigma**	**standard deviation**	**age range cal BP**	**comment**	**Reference**
GrM-22169	46999448-1434	A	bone	-24.54	14.6	5.0	3.2		7760	35	8534	47	8598-8430	top of layer A	this study
KIA-22754	-	B1	charcoal	-22.23 +/-0.09	-	-	-	0.50+/-0.07	42,520	+1200/-1040	45,361*	1200*	47,936-43,232*	Area 3, above layer B	[15]
GrM-22168	46999448-1432	A/B	bone	-21.70	12.8	1.3	3.1		>45,000	-	-	-		mix of layers A and B, minimum age	this study
GrM-22165	46999448-1119	B	bone	-21.00	13.4	2.1	3.1		>45,000	-	-	-		minimum age	this study
GrM-22232	46999448-1055	B	bone	-20.88	42.4	8.5	3.2		>45,000	-	-	-		minimum age	this study
GrM-22164	46999448-1069	B	bone	-21.31	10.1	0.9	3.1		>45,000	-	-	-		minimum age	this study
GrM-22166	46999448-1127	B	bone	-21.93	14.2	4.1	3.1		>45,000	-	-	-		minimum age	this study
GrM-22167	46999448-1238	C	bone	-	-	-	-		-	-	-	-		no collagen	this study
GrM-22170	46999448-1542	C	bone	-	-	-	-		-	-	-	-		no collagen	this study
*Computed with SD +/- 1200 ka															
all calibrated with OxCal 4.4 and IntCal20 calibration curve															

**ESR**							
**LabID**	**FindID**	**Layer**	**material**	**US-ESR age (ka)**	**CSUS-ESR age (ka)**	**age range (ka)**	**Reference**
Probe167	-	D	bone			64,000-53,000	[14]
	-	D	sediment			131,000-88,000	-
#597	46999448-1201	E2b	tooth	189 +16/-14	234 ± 22	205,000-175,000	this study

Table S3. Linear measurements of tooth pits and puncture from large carnivores on axial and near-epiphysis bone portions. measurements are given in mm.

Taxa	n	Mean Length	Sd Length	Min Length	Max Length	Mean Breadth	Sd Breadth	Min Breadth	Max Breadth	Source
Crocuta crocuta	46	2.71	3.11	0.33	9.1	1.55	1.2	0.21	8.7	[46]
Canis lupus	236	2.49	1.15	0.4	9.41	1.8	0.87	0.16	7.48	[46]
Panthera leo	53	4.23	0.37	1.08	11.9	2.89	0.27	0.7	8.24	[83]
Ursus arctos	152	2.33	1.29	0.1	7.7	2.33	0.98	0.08	6.8	[84]

Table S4. Summary of measurements of experimental (grouped into experiments using wooden tips and stone tips) and archaeological hunting lesions measures taken from the literature used for the metric analysis and LDA.

Groups	Length	Width	Area	Source	Taxa
Wooden Spear	6	3.7	NA	Smith 2003, Fig 5 Left	*Ovis aries*
Wooden Spear	18.6	5.6	33.5	- Fig 5 Right	*Ovis aries*
Wooden Spear	23	13	159	- Fig 4 Middle	*Ovis aries*
Wooden Spear	17.7	15.8	NA	Gaudzinski-Windheuser et al. 2018, Sample 107 entry	*Cervus elaphus*
Wooden Spear	9.1	8.5	NA	- Sample 108 entry	*Cervus elaphus*
Wooden Spear	10.5	11.9	NA	- Sample 110 entry	*Cervus elaphus*
Wooden Spear	10.3	13.8	NA	- Sample 111 entry	*Cervus elaphus*
Wooden Spear	13.6	10	NA	- Sample 113 entry	*Cervus elaphus*
Composite Weapon	31	11	203	Castel 2008, Plate 2. photo 5	*Equus*
Composite Weapon	5.5	2.8	11.4	Letourneux and Pétillon 2008, Fig 5 top	*Bos taurus*
Composite Weapon	3.2	1.4	4.1	- Fig 5 mid	*Bos taurus*
Composite Weapon	3.6	1.4	4.7	- Fig5 bottom R	*Bos taurus*
Composite Weapon	8.3	4	26	Parsons and Badenhorst 2004, Fig 1. left	*Connochaetes taurinus*
Composite Weapon	16.5	7.9	100	- Fig 1. upper middle	*Connochaetes taurinus*
Composite Weapon	7.6	4.1	25.5	- Fig 1. lower middle	*Connochaetes taurinus*
Composite Weapon	32	18.3	328.8	- Fig 1. right	*Connochaetes taurinus*
Composite Weapon	12	8.7	75.1	Duches et al. 2016, Figure 8d	*Ovis musimon/Capra hircus*
Composite Weapon	9.1	4.5	30.1	- Figure 8f	*Ovis musimon/Capra hircus*
Archaeological	15.7	11.6	144.4	Letourneux and Pétillon 2008, Fig10	*Cervus*
Archaeological	11.3	4.9	39	- Fig 3 A-B	*Mammuthus primigenius*
Archaeological	22.3	17.1	262.2	- Fig 3 C-D	*Mammuthus primigenius*
Archaeological	37.6	16.6	616.9	- Fig 4 D	*Mammuthus primigenius*
Archaeological	32.2	25.8	684.8	Leduc 2014, Fig 3	*Alces alces*
Archaeological	19.4	6.6	96.7	- Fig 9 Left	*Sus scrofa*
Archaeological	6.4	2.6	17.6	- Fig 9 Right	*Sus scrofa*
Archaeological	16.7	9.4	121.6	Noe-Nygaard 1974, Fig 20	*Capreolus capreolus*
Archaeological	13.5	6.2	59	- Fig 4	*Cervus elaphus*
Archaeological	19.7	15.8	243.3	- Fig 5	*Cervus elaphus*
Archaeological	41.4	27.6	945.5	- Plate 1c	*Bos primigenius*
Archaeological	73	33.5	2015	- Plate 1d	*Bos primigenius*
Archaeological	19.1	8.7	114	- Plate 3a	*Sus scrofa*
Archaeological	11.1	8.1	77.7	- SI. Figure 10	*Dama dama geiselana*
Archaeological	10.6	8.6	65.6	- SI Fig. 11	*Dama dama geiselana*

Table S5. Detailed ESR results obtained for tooth enamel sample #597. Measurement precision is expressed as the mean coefficient of variation obtained for all the aliquots of a given sample after the three repeated measurements. DE precision is the variation of the DE values derived from the repeated measurements of a given sample.

**Sample**	**#597**
Average weight per aliquot (mg)	12.1 ± 0.2
Number of repeated measurements	3
Measurement precision (%)	0.6
**SSE fitting (data weighting by 1/I2)**	
Non-corrected ESR intensities	
DE precision (%)	3.4
Adj. r-Square	0.999
DE1 (Gy)	252.8 ± 7.48
Dmax (Gy)	1482
Dmax/DE	5.9
**SSE fitting (data weighting by 1/s2)**	
Non-corrected ESR intensities	
Adj. r-Square	1.000
DE2 (Gy)	250.0 ± 8.23
Dmax (Gy)	1482
Dmax/DE5	5.9
DE ratios	
DE2/DE1 ratio	0.99

Table S6. U-series data obtained from solution ICP-MS analyses of the powdered dental tissues. Corrected ^{230}Th age are calculated by applying non-radiogenic ^{230}Th correction, assuming non-radiogenic ^{230}Th/^{232}Th = 0.825+/-50% (bulk-Earth value), with ^{238}U, ^{234}U, ^{232}Th and ^{230}Th in secular equilibrium. Errors are 2σ.

Sample ID	Tissue	U (ppm)	230Th/ 232Th	230Th/238U	234U/ 238U	uncorr. 230Th age (ka)	corr. 230Th age (ka)	corr. Initial (234U/ 238U)
#597	Dentine	50.234 ± 0.098	21352 ± 185	0.9484 ± 0.0024	1.2267 ± 0.0016	150.0 ± 0.9	150.0 ± 0.9	1.3462 ± 0.0022
	Enamel	0.952 ± 0.000	492 ± 1	0.9396 ± 0.0025	1.3117 ± 0.0022	127.6 ± 0.7	127.5 ± 0.7	1.4473 ± 0.0028

Table S7. ICP-MS analytical results for the two sediment samples collected from the surrounding of tooth #597. Associated error to the mean values corresponds to the mean of the individual errors, while the coefficient of variation (%) is the standard deviation to the mean.

Sample ID	U (ppm)	Th (ppm)	K (%)
#597sedA	3.33 ± 0.14	2.17 ± 0.10	0.28 ± 0.01
#597sedB	2.69 ± 0.12	1.81 ± 0.09	0.25 ± 0.01
Mean	3.01 ± 0.13 (15.0%)	1.99 ± 0.10 (12.8 %)	0.26 ± 0.01 (6.9 %)

Table S8. Data inputs and outputs associated to the ESR age calculations. All errors are given at a 1σ confidence level. Key: (^{1}) from Grün and Katzenberger [63]; (^{2}) A 10% error was assumed.

SAMPLE	#597
**Enamel**	
Dose (Gy)	253 ± 9
U (ppm), 234U/238U, 230Th/234U	See Table S6
Alpha Efficiency (1)	0.13 ± 0.02
Water content (%)	0
Initial enamel thickness (µm) (2)	913 ± 91
**Dentine**	
U (ppm), 234U/238U, 230Th/234U	See Table S6
Water (%) (3)	5 ± 3
Removed enamel thickness (µm) (2)	143 ± 14
**Sediment**	
U (ppm), Th (ppm), K (ppm)	See Table S7
Water (%)	20 ± 5
Removed thickness (µm) (2)	70 ± 7
**US-ESR age calculations**	
internal dose rate (µGy/a)	262 ± 45
beta dose rate, dentine (µGy/a)	566 ± 82
beta dose rate, cement (µGy/a)	77 ± 8
Gamma + cosmic dose rate (µGy/a)	428 ± 29
Total dose rate (µGy/a)	1334 ± 98
p enamel	-0.69
p dentine	-0.84
US-ESR age (ka)	189 +16 -14
CSUS-ESR age (ka)	234 ± 22

Table S9. Einhornhöhle. Faunal assemblage of layer H.

	Layer H					
**Taxa**	**NISP**	**%**	**Cm**	**%**	**Bm**	**%**
Canis lupus	3	1.08	0	0.00	0	0.00
Panthera spelaea	3	1.08	1	0.36	0	0.00
Ursus cf. spelaeus	221	79.78	0	0.00	17	6.14
Indet. large carnivore	20	7.22	0	0.00	9	3.25
Indet. ungulate	3	1.08	0	0.00	1	0.36
Indet. mammal	27	9.75	0	0.00	4	1.44
Total	277		1	0.36	31	11.19

Table S10. Einhornhöhle. MNE values of teeth and crania and the ratio of tooth MNE to cranial MNE for *Ursus* (*cf.*) *spelaeus* in layer H.

Layer	Tooth MNE	Bone MNE	Tooth:Bone MNE
H	13	2	6.5

Table S11. Einhornhöhle. Measurements of the cave lion remains from layer H in mm. L = Length; B = Breadth.

ID	Layer	Element	Surface Modifications	L	B
46999448_1397	H	Phalanx III		-	16.7
46999448_1384	H	Phalanx III	C	-	18.7
45453992_28	H	Sesamoid		30.7	-

Table S12. Einhornhöhle. Cave bear MAU value layer H

Bear Skeleton	MNE in Skeleton	MNE	MAU
Head	4	2	0.5
Neck	7	1	0.14
Axial	49	25	0.51
Upper Front	4	2	0.5
Lower Front	14	2	0.14
Upper Hind	2	3	1.5
Lower Hind	16	2	0.12
Feet	60	22	0.37
Total	156	69	

Table S13. Cave lion from Siegsdorf. List of the microscopically examined cave-lion bones and the main incisions found.

Specimen	ID	Modifications
Phalanx III	46999448_1384	Cut marks
Rib II (right)	NKM527/6	Drag mark, cut marks
Rib III (left)	NKM527/21	Drag mark, cut marks
Rib III (right)	NKM527/7	Projectile impact fracture
Rib VI (right)	NKM527/10	Cut marks, trampling
Femur (right)	NKM527/1	Cut marks
Lumbar vertebra III	NKM527/36	Notch
Pelvic bone	NKM527/18	Cut marks

Table S14. Cave lion from Siegsdorf. Raw morphometric values for each cross-sectional profile measured on the cave-lion bone incisions. M = magnification; WIS = width at bone surface; WIM = width at the mean; WIB = width at the bottom (only in the case of single incisions, otherwise the depth is featured with an asterisk); D = depth of the incision; OA = opening angle. WIS, WIB, and D are given in µm, while OA is given in degree. Categories after Bello et al.[35]. If = Impact fracture; Cm = cut mark; Dm = drag mark; Nt = Notch. The area lettering indicates different incisions. In the case the same incision was examined multiple times from different position, then the letter was additionally featured with a number. For each bone and type of incision, the averaged value is given with the standard deviation.

Bone	Type	Area	M	WIS	WIM	WIB	D	OA
Femur (right)	Cm	A	500×	195.4	106.1	42.5	28.3	66.5
Femur (right)	Cm	B	500×	248.5	95.0	34.9	43.9	64.5
Femur (right)	Cm	C	500×	291.7	96.6	38.7	30.9	86.3
Femur (right)	Cm	D	500×	169.5	77.2	43.6	14.0	70.3
Femur (right)	Cm	E	500×	174.1	77.2	40.2	20.7	97.1
Femur (right)	Cm	F.1	1000×	198.8	95.6	36.4	36.3	97.4
Femur (right)	Cm	F.2	1000×	176.0	79.7	33.5	19.6	96.8
Femur (right)	Cm	G	1000×	242.8	115.1	46.4	35.1	70.8
Lumbar	Nt	A.1	100×	3276.3	1347.8	385.3	1068.6	61.7
Lumbar	Nt	A.2	100×	3162.5	1408.4	415.2	1122,4	71.5
Lumbar	Nt	A.3	100×	1635.9	870.0	386.7	479.4	105.4
Lumbar	Nt	B.1	100×	3059.6	1482.5	-	1026.1	60.7
Lumbar	Nt	B.2	100×	3019.8	1763.7	472.9	1071.2	76.7
Lumbar	Nt	B.3	100×	2245.9	1020.8	256.3	586.4	100.7
Pelvis	Cm	A.1	200×	316.9	208.5	83.2	27.1	70.6
Pelvis	Cm	A.2	200×	399.6	218.8	115.3	52.1	80.4
Pelvis	Cm	A.3	200×	378.9	207.2	101.2	44.5	81.1
Pelvis	Cm	B.1*	200×	323.4	196.3	106.9	19.9	91.0
Pelvis	Cm	B.1*	200×	282.7	153.8	80.4	22.02	87.4
Pelvis	Cm	B.2*	200×	401.1	273.1	212.6	17.0	-
Pelvis	Cm	B.2*	200×	266.3	142.4	79.0	22.4	61.8
Rib II (right)	Dm	A.1	100×	3665.9	2438.5	790.1	714.7	113.7
Rib II (right)	Dm	A.2	100×	3326.6	1707.9	539.4	556.2	91.6
Rib II (right)	Dm	A.3	100×	2240.2	1031.0	312.4	218.7	63.9
Rib II (right)	Dm	A.1	200×	3935.8	2148.7	699.9	832.4	109.1
Rib II (right)	Dm	A.2	200×	2876.5	1499.8	373.6	602.5	90.5
Rib II (right)	Dm	A.3	200×	2566.4	1380.0	618.0	312.7	82.5
Rib II (right)	Cm	A.1	500×	204.4	99.5	49.6	19.5	106.1
Rib II (right)	Cm	A.2	500×	169.5	95.6	42.9	20.0	101.7
Rib II (right)	Cm	B	500×	120.5	58.8	39.2	12.6	107.3
Rib II (right)	Cm	C	500×	125.4	71.0	41.8	12.6	84.9
Rib II (right)	Cm	D	500×	173.1	92.9	41.2	22.4	105.8
Rib III (left)	Dm	A.1	150×	5629.6	2641.4	472.7	1205.6	80.4
Rib III (left)	Dm	A.2	150×	3723.7	2270.8	648.8	760.2	91.7
Rib III (left)	Dm	A.3	150×	2236.6	1058.6	466.7	118.5	52.0
Rib III (left)	Dm	A.4	150×	5217.9	2722.2	441.5	162.3	100.4
Rib III (left)	Dm	A.5	150×	4776.1	2237.1	1091.2	608.7	111.7
Rib III (left)	Dm	A.6	150×	2198.7	1124.9	509.8	114.0	127.4
Rib III (left)	Cm	A.1	1000×	76.0	37.6	17.4	6.36	98.7
Rib III (left)	Cm	A.2	1000×	98.0	47.6	19.7	8.2	98.0
Rib III (right)	If	Max	100×	12159.5	6736.0	3320.9		104.3
Rib III (right)	If	Min	100×	7484.8	4030.2	2176.6	3291.8	101.9
Rib III (right)	If	Max	200×	11246.7	4886.8	2357.3	3222.1	110.4

Rib III (right)	If	Min	200×	7469.0	3923.3	1846.6	3468.7	97.6
Rib III (right)	If	Vert	200×	9355.1	5215.6	2487.3	3413.7	102.9
Rib III (right)	If	Hor	200×	7417.0	3890.3	1765.0	3471.1	100.6
Rib III (right)	If	Max	50×	11826.1	4976.7	2310.1	2510.8	98.2
Rib III (right)	If	Min	50×	8080.9	3799.7	1695.9	3342.9	105.5
Rib III (right)	If	Vert	50×	9578.7	4680.7	2257.4	3032.4	100.3
Rib III (right)	If	Hor	50×	8040.1	4668.2	1972.3	3318.3	105.1
Rib III (right)	If	Max	100×				3811.0	
Rib III (right)	If	Min	100×	11739.6	5446.3	2510,7	3142.6	100.8
Rib III (right)	If	Max	100×	7635.3	4295.3	1519.6	3480.9	92.0
Rib III (right)	IF	Max	200×	12618.49	6351.98	2510.49	3278.87	113.21
Rib III (right)	IF	Min	200×	9235.12	4212.62	1886.28	4159.08	104.52
Rib VI (right)	Dm	A.1*	200×	717.8	486.3	327.2	92.6	80.9
Rib VI (right)	Dm	A.1*	200×	886.9	440.7	225.8	140.7	72.1
Rib VI (right)	Dm	A.2*	200×	1068.9	731.3	490.2	98.4	84.9
Rib VI (right)	Dm	A.2*	200×	1029.7	556	199.6	140.5	60.0

Table S15. Cave lion from Siegsdorf. Averaged values of the morphometric data based on profile lines. Raw data are listed in Table S14. WIS = width at bone surface; WIM = width at the mean; WIB = width at the bottom; D = depth of the incision; OA = opening angle. WIS, WIB, and D are given in μm, while OA is given in degree. Categories after Bello et al.[35]. If = Impact fracture. Cm = cut mark; Dm = drag mark; Nt = Notch.

Bone	Type	WIS	WIM	WIB	D	OA
Femur	Cm	212	93	40	28	81
Pelvis	Cm	338	200	112	29	79
Rib III (left)	Cm	87	43	19	7	98
Rib II (right)	Dm	3102	1701	556	540	92
Rib III (left)	Dm	3964	2009	605	496	93
Rib VI (right)	Dm	926	554	311	118	103
Lumbar	Nt	2733	1316	383	892	80
Rib III (right)	If min	11743	5511	2625	3172	104
	If max	7668	4012	1810	3368	102

Table S16. Linear measurements of pits and punctures on ribs created by lions, and on the whole skeleton created by spotted hyenas. Measurements are given in mm.

Taxa	n	Mean Depth	Sd Depth	Min Depth	Max Depth	Source
Panthera leo	36	1.2	0.9	0.1	3.7	Pobiner 2007
Crocuta crocuta	9	1.91	0.93	0.2	2.83	Pobiner 2007

Table S17. Jackknifed classifier provided by LDA analysis. Siegsdorf hunting lesion is classified as Wooden Spear.

Point	Given group	Classification	Jackknifed
Smith 2003, Fig 5 (left)	Wooden Spear	Composite Weapon	Composite Weapon
Smith 2003, Fig 5 (right)	Wooden Spear	Composite Weapon	Composite Weapon
Smith 2003, Fig 4 Middle	Wooden Spear	Archaeological Lesion	Archaeological Lesion
Gaudzinski-Windheuser et al 2018, Sample 107 entry	Wooden Spear	Wooden Spear	Wooden Spear
Gaudzinski-Windheuser et al 2018, Sample 108 entry	Wooden Spear	Wooden Spear	Wooden Spear
Gaudzinski-Windheuser et al 2018, Sample 110 entry	Wooden Spear	Wooden Spear	Wooden Spear
Gaudzinski-Windheuser et al 2018, Sample 111 entry	Wooden Spear	Wooden Spear	Wooden Spear
Gaudzinski-Windheuser et al 2018, Sample 113 entry	Wooden Spear	Wooden Spear	Wooden Spear
Castel 2008 Plate 2, photo 5	Composite Weapon	Archaeological Lesion	Archaeological Lesion
Letourneux & Pétillon 2008, Fig5 top	Composite Weapon	Composite Weapon	Composite Weapon
Letourneux & Pétillon 2008, Fig5 mid	Composite Weapon	Composite Weapon	Composite Weapon
Letourneux & Pétillon 2008, Fig5 bot R	Composite Weapon	Composite Weapon	Composite Weapon
Parsons & Badenhorst 2004, Fig 1, (left)	Composite Weapon	Composite Weapon	Composite Weapon
Parsons & Badenhorst 2004, Fig 1, upper middle	Composite Weapon	Composite Weapon	Composite Weapon
Parsons & Badenhorst 2004, Fig 1, lower middle	Composite Weapon	Composite Weapon	Composite Weapon
Parsons & Badenhorst 2004, Fig 1, (right)	Composite Weapon	Archaeological Lesion	Archaeological Lesion
Duches et al. 2016, Fig 8d	Composite Weapon	Wooden Spear	Wooden Spear
Duches et al. 2016, Fig 8f	Composite Weapon	Composite Weapon	Composite Weapon
Letourneux & Pétillon 2008, Fig10	Archaeological Lesion	Wooden Spear	Wooden Spear
Nikolskiy & Pitulko 2013, Fig 3 A-B	Archaeological Lesion	Composite Weapon	Composite Weapon
Nikolskiy & Pitulko 2013, Fig 3 C-D	Archaeological Lesion	Wooden Spear	Wooden Spear
Nikolskiy & Pitulko 2013, Fig 4 D	Archaeological Lesion	Archaeological Lesion	Archaeological Lesion
Leduc 2012, Fig 3	Archaeological Lesion	Wooden Spear	Wooden Spear
Leduc 2012, Fig 9 (left)	Archaeological Lesion	Composite Weapon	Composite Weapon
Leduc 2012, Fig 9 (right)	Archaeological Lesion	Composite Weapon	Composite Weapon
Noe-Nygaard 1974, Fig 20	Archaeological Lesion	Archaeological Lesion	Archaeological Lesion
Noe-Nygaard 1974, Fig 4	Archaeological Lesion	Composite Weapon	Composite Weapon
Noe-Nygaard 1974, Fig 5	Archaeological Lesion	Wooden Spear	Wooden Spear
Noe-Nygaard 1974, Plate 3a	Archaeological Lesion	Composite Weapon	Composite Weapon
Gaudzinski-Windheuser et al. 2018, SI, Figure 10	Archaeological Lesion	Wooden Spear	Wooden Spear
Gaudzinski-Winheuser et al. 2018, SI Fig. 11	Archaeological Lesion	Wooden Spear	Wooden Spear
Siegsdorf	?	Wooden Spear	

Table S18. Classification dataset PIMs**.** Jackknifed classification matrix. Overall jackknifed classification success: 45.16%.

	Wooden Spear	Composite Weapon	Archaeological Lesion	Total
**Wooden Spear**	5	2	1	8
**Composite Weapon**	1	7	2	10
**Archaeological Lesion**	6	5	2	13
**Total**	12	14	5	31

Table S19. Einhornhöhle. Summary of the taphonomic data of layer H. Values are given in NISP and NISP%.

Layer H	Cut marks	Cut mark%	Carnivore gnawing marks	Carnivore gnawing marks%	Weathering	Weathering%	Root etching	Abrasion/polishing/corrosion
277	1	0,36	31	11,19	36	13,00	0	0

Table S20. Einhornhöhle. Averaged values of the morphometric data based on profile lines. WIS = width at bone surface; WIM = width at the mean; WIB = width at the bottom; D = depth of the incision; OA = opening angle. WIS, WIB, and D are given in µm, while OA is given in degree. Categories after Bello et al.[35]. Lcm = longer cut mark; Scm = shorter cut mark.

Bone	Type	WIS	WIM	WIB	D	OA
Phalanx III	Lcm	185	148	65	30	100
	Lcm max	215	179	87	40	76
	Lcm min	122	114	42	21	129
	Scm	389			43	99
	Scm max	437			50	72
	Scm min	317			37	106

6. References SI

1. Rosendahl, W., Darga, R. & Döppes, D. Mammuthus-Coelodonta Faunal Complex from Siegsdorf (Chiemgau, Germany) – Overview and New Analyses. in *The Beef behind all Possible Pasts: The Tandem Festschrift in Honour of Elaine Turner and Martin Street* (eds. Gaudzinski-Windheuser, S. & Jöris, O.) 173–183 (Monographien des RGZM, 2021). doi:https://doi.org/10.11588/propylaeum.868.c11312.
2. Rosendahl, W. & Darga, R. Klima, Umwelt und Mensch im Oberpleistozan des Chiemgaus - neue Daten und Befunde. *Terra Nostra* **6**, 305–309 (2002).
3. Gross, C. Das Skelett des Höhlenöwen (Panthera leo spelaea Goldfuss 1810) aus Siegsdorf/ Ldkr. Traunstein im Vergleich mit anderen Funden aus Deutschland und den Niederlanden. (Univ. Munich, 1992).
4. Argant, A. & Brugal, J.-P. The cave lion Panthera (Leo) spelaea and its evolution: Panthera spelaea intermedia nov. subspecies . *Acta Zool. Cracoviensia* **60**, 58–103 (2018).
5. Marciszak, A. *et al.* The Pleistocene lion Panthera spelaea (Goldfuss, 1810) from Poland – A review. *Quat. Int.* **605**–**606**, 213–240 (2021).
6. Marciszak, A., Schouwenburg, C. & Darga, R. Decreasing size process in the cave (Pleistocene) lion Panthera spelaea (Goldfuss, 1810) evolution – A review. *Quat. Int.* **339**–**340**, 245–257 (2014).
7. Burger, J. *et al.* Molecular phylogeny of the extinct cave lion Panthera leo spelaea. *Mol. Phylogenet. Evol.* **30**, 841–849 (2004).
8. Stanton, D. W. G. *et al.* Early Pleistocene origin and extensive intra-species diversity of the extinct cave lion. *Sci. Reports 2020 101* **10**, 1–7 (2020).
9. Leder, D. *et al.* A 51,000-year-old engraved bone reveals Neanderthals' capacity for symbolic behaviour. *Nat. Ecol. Evol. 2021 59* **5**, 1273–1282 (2021).
10. Kotula, A. *et al.* Eiszeitliche Besiedlung in Niedersachsens Höhlen: Neue Forschungen an der Einhornhöhle im Harz. Ldkr. Göttingen. *Nachr. Nieders. Urgesch.* **88**, 211–229 (2019).
11. Scheer, A. Mittelpaläolithische Funde in der Einhornhöhle bei Scharzfeld. *Nachr. Nieders. Urgesch.* **55**, 1–39 (1988).
12. Veil, S. Die archäologisch-geowissenschaftlichen Ausgrabungen 1987/88 in der Einhornhöhle bei Scharzfeld. Ldkr. Osterode am Harz. *Archäolog. Korr.-Blatt.* **19**, (1989).
13. Leder, D. *et al.* Neandertaler und Symbole. Neue Forschungen zur Einhornhöhle im Harz, Ldkr. Göttingen. *Nachr. Nieders. Urgesch.* 11–42 (2021).
14. Nielbock, R. Holozäne und jungpleistozäne Wirbeltierfaunen der Einhornhöhle im Harz - Paläontologisch-biostratigraphische Untersuchungsergebnisse der Höhlengrabung. (University Clausthal, 1987).
15. Baier, S. Frühweichselzeitliche feinlaminierte Sedimente der Einhornhöhle bei Scharzfeld / Harz. (Johannes-Gutenberg-Universität Mainz, 2004).
16. Lisiecki, L. E. & Raymo, M. E. A Pliocene-Pleistocene stack of 57 globally distributed benthic δ18O records. *Paleoceanography* **20**, 1–17 (2005).
17. Hillson, S. *Teeth, second edition*. *Teeth, Second Edition* (Cambridge University Press, 2005). doi:10.1017/CBO9780511614477.
18. France, D. L. *Human and Nonhuman Bone Identification : A Color Atlas*. *Human and Nonhuman Bone Identification* (CRC Press, 2008). doi:10.1201/9781420062878.
19. Grayson, D. K. *Quantitative Zooarchaeology*. (Elsevier, 1984). doi:10.1016/C2009-0-

21855-1.

20. Lyman, R. L. *Quantitative paleozoology*. *Quantitative Paleozoology* (Cambridge University Press, 2008). doi:10.1017/CBO9780511813863.

21. Lyman, R. L. *Vertebrate taphonomy*. *Vertebrate Taphonomy* (Cambridge University Press, 2014). doi:10.1017/CBO9781139878302.

22. Stiner, M. C. Food procurement and transport by human and non-human predators. *J. Archaeol. Sci.* **18**, 455–482 (1991).

23. Binford, L. R. *Nunamiut Ethnoarchaeology*. (Academic Press, 1978).

24. Homberger, D. G. *et al.* The structure of the cornified claw sheath in the domesticated cat (Felis catus): implications for the claw-shedding mechanism and the evolution of cornified digital end organs. *J. Anat.* **214**, 620–643 (2009).

25. Gaston, K. J. *Species richness: measure and measurement*. *Biodiversity: a biology of numbers and difference* (Blackwell Science, 1996).

26. Simpson, E. H. Measurement of Diversity. *Nat. 1949 1634148* **163**, 688–688 (1949).

27. Domínguez-Rodrigo, M., de Juana, S., Galán, A. B. & Rodríguez, M. A new protocol to differentiate trampling marks from butchery cut marks. *J. Archaeol. Sci.* **36**, 2643–2654 (2009).

28. Fernández-Jalvo, Y. & Andrews, P. *Atlas of taphonomic identifications*. *Vertebrate Paleobiology and Paleoanthropology* (2016). doi:10.1007/978-94-017-7432-1.

29. Stiner, M. C. *Honor among thieves: A zooarchaeological study of neandertal ecology.* (Princeton: Princeton University Press, 1994).

30. Stiner, M. C. *The faunas of Hayonim Cave (Israel) : a 200,000-year record of Paleolithic diet, demography, and society*. (Peabody Museum of Archaeology and Ethnology, Harvard University, 2005).

31. Behrensmeyer, A. K. Taphonomic and ecologic information from bone weathering. *Paleobiology* **4**, 150–162 (1978).

32. Starkovich, B. M. Trends in Subsistence from the Middle Paleolithic through Mesolithic at Klissoura Cave 1 (Peloponnese, Greece). (The University of Arizona., 2011).

33. Bello, S. M. & Soligo, C. A new method for the quantitative analysis of cutmark micromorphology. *J. Archaeol. Sci.* **35**, 1542–1552 (2008).

34. Bello, S. M. *New Results from the Examination of Cut-Marks Using Three-Dimensional Imaging*. *Developments in Quaternary Science* vol. 14 (Elsevier, 2011).

35. Bello, S. M., de Groote, I. & Delbarre, G. Application of 3-dimensional microscopy and micro-CT scanning to the analysis of Magdalenian portable art on bone and antler. *J. Archaeol. Sci.* **40**, 2464–2476 (2013).

36. Maté-González, M. Á. *et al.* Assessment of statistical agreement of three techniques for the study of cut marks: 3D digital microscope, laser scanning confocal microscopy and micro-photogrammetry. *J. Microsc.* **267**, 356–370 (2017).

37. Maté-González, M. Á., Palomeque-González, J. F., Yravedra, J., González-Aguilera, D. & Domínguez-Rodrigo, M. Micro-photogrammetric and morphometric differentiation of cut marks on bones using metal knives, quartzite, and flint flakes. *Archaeol. Anthropol. Sci.* **10**, 805–816 (2018).

38. Maté-González, M. Á. *et al.* Flint and Quartzite: Distinguishing Raw Material Through Bone Cut Marks. *Archaeometry* **60**, 437–452 (2018).

39. Schindelin, J. *et al.* Fiji: an open-source platform for biological-image analysis. *Nat.*

Methods 2012 97 **9**, 676–682 (2012).

40. Nikolskiy, P. & Pitulko, V. Evidence from the Yana Palaeolithic site, Arctic Siberia, yields clues to the riddle of mammoth hunting. *J. Archaeol. Sci.* **40**, 4189–4197 (2013).

41. Smith, G. M. *et al.* When Lithics Hit Bones: Evaluating the Potential of a Multifaceted Experimental Protocol to Illuminate Middle Palaeolithic Weapon Technology. *J. Paleolit. Archaeol. 2020 32* **3**, 126–156 (2020).

42. Letourneux, C. & Pétillon, J. M. Hunting lesions caused by osseous projectile points: experimental results and archaeological implications. *J. Archaeol. Sci.* **35**, 2849–2862 (2008).

43. Duches, R. *et al.* Identification of Late Epigravettian hunting injuries: Descriptive and 3D analysis of experimental projectile impact marks on bone. *J. Archaeol. Sci.* **66**, 88–102 (2016).

44. Gaudzinski-Windheuser, S. *et al.* Evidence for close-range hunting by last interglacial Neanderthals. *Nat. Ecol. Evol.* **2**, 1087–1092 (2018).

45. Domínguez-Rodrigo, M. & Piqueras, A. The use of tooth pits to identify carnivore taxa in tooth-marked archaeofaunas and their relevance to reconstruct hominid carcass processing behaviours. *J. Archaeol. Sci.* **30**, 1385–1391 (2003).

46. Andrés, M., Gidna, A. O., Yravedra, J. & Domínguez-Rodrigo, M. A study of dimensional differences of tooth marks (pits and scores) on bones modified by small and large carnivores. *Archaeol. Anthropol. Sci.* **4**, 209–219 (2012).

47. Camarós, E., Münzel, S. C., Cueto, M., Rivals, F. & Conard, N. J. The evolution of Paleolithic hominin-carnivore interaction written in teeth: Stories from the Swabian Jura (Germany). *J. Archaeol. Sci. Reports* **6**, 798–809 (2016).

48. Delaney-Rivera, C. *et al.* Pits and pitfalls: taxonomic variability and patterning in tooth mark dimensions. *J. Archaeol. Sci.* **36**, 2597–2608 (2009).

49. Gaudzinski-Windheuser, S. Hunting lesions in Pleistocene and early Holocene European bone assemblages and their implications for our knowledge on the use and timing of lithic projectile technology. in *Multidisciplinary Approaches to the Study of Stone Age Weaponry* (eds. Iovita, R. & Sano, K.) 77–100 (Springer, 2016). doi:10.1007/978-94-017-7602-8_6/COVER.

50. RStudio_Team. Integrated Development fo R. RStudio. (2020).

51. Domínguez-Rodrigo, M. *et al.* Artificial intelligence provides greater accuracy in the classification of modern and ancient bone surface modifications. *Sci. Reports 2020 101* **10**, 1–11 (2020).

52. Hammer, Ø., Harper, D. A. T. & Ryan, P. D. PAST: paleontological statistics software package for education and data analysis. *Palaeontol. Electron.* **4**, 1–9 (2001).

53. Noe-Nygaard, N. Mesolithic hunting in Denmark illustrated by bone injuries caused by human weapons. *J. Archaeol. Sci.* **1**, 217–248 (1974).

54. Coppe, J., Lepers, C. & Rots, · Veerle. Projectiles Under a New Angle: a Ballistic Analysis Provides an Important Building Block to Grasp Paleolithic Weapon Technology. *J. Archaeol. Method Theory 2022* 1–27 (2022) doi:10.1007/S10816-022-09551-Z.

55. Iovita, R., Schönekeß, H., Gaudzinski-Windheuser, S. & Jäger, F. Projectile impact fractures and launching mechanisms: results of a controlled ballistic experiment using replica Levallois points. *J. Archaeol. Sci.* **48**, 73–83 (2014).

56. Münzel, S. C. & Conard, N. J. Cave bear hunting in the Hohle Fels, a cave site in the Ach valley, Swabian Jura. *Rev. Paléobiologie* **23**, 877–885 (2004).

57. Carr, D. J. & Wainwright, A. Variability of simulants used in recreating stab events. *Forensic Sci. Int.* **210**, 42–46 (2011).

58. Wilkins, J., Schoville, B. J. & Brown, K. S. An Experimental Investigation of the Functional Hypothesis and Evolutionary Advantage of Stone-Tipped Spears. *PLoS One* **9**, e104514 (2014).

59. Grün, R. Methods of dose determination using ESR spectra of tooth enamel. *Radiat. Meas.* **32**, 767–772 (2000).

60. Zhao, J. X., Hu, K., Collerson, K. D. & Xu, H. K. Thermal ionization mass spectrometry U-series dating of a hominid site near Nanjing, China. *Geology* **29**, 27–30 (2001).

61. Clark, T. R. *et al.* Discerning the timing and cause of historical mortality events in modern Porites from the Great Barrier Reef. *Geochim. Cosmochim. Acta* **138**, 57–80 (2014).

62. Ludwig, K. Isoplot/Ex, v. 3.75, A Geochronological Toolkit for Microsoft Excel. *Berkeley Geochronol. Cent. Spec. Publ.* **5**, 75 (2012).

63. Grün, R. & Katzenberger-Apel, O. An alpha irradiator for ESR dating. *Anc. TL* **12**, 35–38 (1994).

64. Marsh, R. E. Beta-gradient Isochrons Using Electron Paramagnetic Resonance: Towards a New Dating Method in Archaeology. (McMaster University, 1999).

65. Guérin, G., Mercier, N. & G., A. Dose-rate conversion factors: update. *Anc. TL* **29**, 5–8 (2011).

66. Grün, R. The DATA program for the calculation of ESR age estimates on tooth enamel. *Quat. Geochronol.* **4**, 231–232 (2009).

67. Grün, R., Schwarcz, H. P. & Chadam, J. ESR dating of tooth enamel: Coupled correction for U-uptake and U-series disequilibrium. *Int. J. Radiat. Appl. Instrumentation. Part D. Nucl. Tracks Radiat. Meas.* **14**, 237–241 (1988).

68. Grün, R. Open Research: An alternative model for open system U-series/ESR age calculations: (closed system U-series)-ESR, CSUS-ESR. *Anc. TL* **18**, 1–4 (2000).

69. Ziegler, R. *Das Mammut (Mammuthus primigenius Blumenbach) von Siegsdorf bei Traunstein (Bayern) und seine Begleitfauna*. (Pfeil, 1994).

70. O'Driscoll, C. A. & Thompson, J. C. Experimental projectile impact marks on bone: implications for identifying the origins of projectile technology. *J. Archaeol. Sci.* **49**, 398–413 (2014).

71. Domínguez-Solera, S. & Domínguez-Rodrigo, M. A taphonomic study of a carcass consumed by griffon vultures (Gyps fulvus) and its relevance for the interpretation of bone surface modifications. *Archaeol. Anthropol. Sci.* **3**, 385–392 (2011).

72. Schouwenburg, C., Darga, R. & Rosendahl, W. De grottenleeuw, Panthera leo spelaea (Goldfuss 1810), uit Siegsdorf, Duitsland. *Cranium* **26**, 31–40 (2009).

73. Chapman, S. *Chapman & Nakielny's aids to radiological differential diagnosis*. (2019).

74. Valtierra, N., Courtenay, L. A. & López-Polín, L. Microscopic analyses of the effects of mechanical cleaning interventions on cut marks. *Archaeol. Anthropol. Sci.* **12**, 1–18 (2020).

75. Domínguez-Rodrigo, M., de Juana, S., Galán, A. B. & Rodríguez, M. A new protocol to differentiate trampling marks from butchery cut marks. *J. Archaeol. Sci.* **36**, 2643–2654 (2009).

76. Bello, S. M., Parfitt, S. A. & Stringer, C. Quantitative micromorphological analyses of cut marks produced by ancient and modern handaxes. *J. Archaeol. Sci.* **36**, 1869–1880

(2009).

77. Domínguez-Rodrigo, M., Pickering, T. R. & Bunn, H. T. Experimental study of cut marks made with rocks unmodified by human flaking and its bearing on claims of ∼3.4-million-year-old butchery evidence from Dikika, Ethiopia. (2012) doi:10.1016/j.jas.2011.03.010.

78. Bello, S. M., Wallduck, R., Dimitrijević, V., Živaljević, I. & Stringer, C. B. Cannibalism versus funerary defleshing and disarticulation after a period of decay: comparisons of bone modifications from four prehistoric sites. *Am. J. Phys. Anthropol.* **161**, 722–743 (2016).

79. Wallduck, R. & Bello, S. M. Cutting decaying bodies: Micro-morphometric analysis of cut-marks on Mesolithic-Neolithic human remains from Lepenski Vir and Vlasac, Serbia. *J. Archaeol. Sci. Reports* **10**, 703–714 (2016).

80. Milks, A., Champion, S., Cowper, E., Pope, M. & Carr, D. Early spears as thrusting weapons: Isolating force and impact velocities in human performance trials. *J. Archaeol. Sci. Reports* **10**, 191–203 (2016).

81. Duval, M. & Grün, R. Are published ESR dose assessments on fossil tooth enamel reliable? *Quat. Geochronol.* **31**, 19–27 (2016).

82. Elliott, J. C. Calcium Phosphate Biominerals. *Rev. Mineral. Geochemistry* **48**, 427–453 (2002).

83. Pobiner, B. L. Hominin-carnivore interactions: evidence from modern carnivore bone modification and early Pleistocene archaeofaunas (Koobi Fora, Kenya; Olduvai Gorge, Tanzania). (State University of New Jersey, 2007).

84. Arilla, M., Rosell, J. & Blasco, R. A neo-taphonomic approach to human campsites modified by carnivores. *Sci. Reports 2020 101* **10**, 1–15 (2020).

85. Smith, G. M. Damage inflicted upon animal bone by wooden projectiles: Experimental results and archaeological implications. *J. Taphon.* (2003).

86. Castel, J.-C. Identification des impacts de projectiles sur le squelette des grands ongulés. *Ann. paléontologie* **94**, 103–118 (2008).

87. Parsons, I. & Badenhorst, S. Analysis of lesions generated by replicated Middle Stone Age lithic points on selected skeletal elements. doi:10.10520/EJC96275.

88. Leduc, C. New Mesolithic Hunting Evidence from Bone Injuries at Danish Maglemosian Sites: Lundby Mose and Mullerup (Sjælland). *Int. J. Osteoarchaeol.* **24**, 476–491 (2014).
